# microRNA evolution in a human transcription factor and microRNA regulatory network

**DOI:** 10.1186/1752-0509-4-90

**Published:** 2010-06-29

**Authors:** Chengxiang Qiu, Juan Wang, Pengying Yao, Edwin Wang, Qinghua Cui

**Affiliations:** 1 Department of Biomedical Informatics, Peking University Health Science Center, 38 Xueyuan Rd, Beijing 100191, China; 2 Computational Chemistry and Biology Group, Biotechnology Research Institute, National Research Council Canada, 6100 Royalmont Avenue, Montreal, Quebec, H4P2R2, Canada

## Abstract

**Background:**

microRNAs (miRNAs) are important cellular components. The understanding of their evolution is of critical importance for the understanding of their function. Although some specific evolutionary rules of miRNAs have been revealed, the rules of miRNA evolution in cellular networks remain largely unexplored. According to knowledge from protein-coding genes, the investigations of gene evolution in the context of biological networks often generate valuable observations that cannot be obtained by traditional approaches.

**Results:**

Here, we conducted the first systems-level analysis of miRNA evolution in a human transcription factor (TF)-miRNA regulatory network that describes the regulatory relations among TFs, miRNAs, and target genes. We found that the architectural structure of the network provides constraints and functional innovations for miRNA evolution and that miRNAs showed different and even opposite evolutionary patterns from TFs and other protein-coding genes. For example, miRNAs preferentially coevolved with their activators but not with their inhibitors. During transcription, rapidly evolving TFs frequently activated but rarely repressed miRNAs. In addition, conserved miRNAs tended to regulate rapidly evolving targets, and upstream miRNAs evolved more rapidly than downstream miRNAs.

**Conclusions:**

In this study, we performed the first systems level analysis of miRNA evolution. The findings suggest that miRNAs have a unique evolution process and thus may have unique functions and roles in various biological processes and diseases. Additionally, the network presented here is the first TF-miRNA regulatory network, which will be a valuable platform of systems biology.

## Background

microRNAs (miRNAs) are a class of endogenous and small non-coding regulatory RNAs, which regulate genes at the post-transcriptional level [1]. In the past few years, studies of miRNAs have ranged from their biological functions to their evolution. Understanding the evolution of miRNAs is very important to the study of their function, genomic organization, human disease, and medicine [2,3]. Studies of miRNA evolution have focused on the molecular level. For example, the majority of miRNAs are conserved during evolution [1,4,5]. The structure of miRNA precursor stem loops exhibits significantly increased mutational robustness in comparison with random RNA sequences with the same stem-loop structure[6]. It was revealed that the genetic robustness observed in miRNA sequences is the byproduct of selection for environmental robustness [7]. Vazquez et al. found that recently evolved miRNAs consistently give rise to long-miRNAs, while ancient miRNAs give rise predominantly to canonical miRNAs in Arabidopsis [8]. An Alu-mediated rapid expansion of miRNA genes in primate-specific miRNAs [9] and a rapid evolution of an X-linked miRNA cluster in primates were observed [10]. Besides the fact that miRNAs are evolutionary conserved, it was observed that some miRNA genes are evolutionarily young [11]. In addition, transposable elements (TE)-derived human miRNAs are less conserved, on average, than non-TE-derived miRNA s[12]. The sequence diversification of duplicated miRNA genes to be accompanied by a change in spatial and temporal expression patterns [13]. Host-virus coevolution may affect miRNA regulatory function [14]. We previously found that miRNAs tend to buffer gene expression variation between closely related species [15] and human-specific miRNAs tend to evolve rapidly and found indications that some human miRNAs seem to be under recent positive selection [3]. Lowly expressed human microRNA genes evolve rapidly [16]. Recently, de Wit et al. revealed a novel mode of miRNA evolution, hairpin shifting [17]. The above cited studies have uncovered some important evolutionary insights, but have, however, not considered the regulatory context of miRNAs. That is, as the primary two classes of gene regulators, miRNAs and transcription factors (TFs) regulate each other and co-regulate other genes. Therefore, considering the regulatory network of miRNAs in such evolutionary studies is increasingly important for an integrated understanding of the subject.

Cells typically change physiologically in response to signals received from their changing internal and external environments [18]. To do this they must activate or repress the production of various gene products and tune these products to the proper level for different situations. Hence, the deregulation of genes may result in phenotypic variations that can contribute to diseases. For example, removing just one molecule of the transcription factor protein, *c-myb*, from the cellular milieu can result in developmental defects in the B cell lineage [19]. The current research perspective is that the level of gene expression is primarily regulated by TFs at the transcriptional level and by miRNAs at the post-transcriptional level. Moreover, TFs and miRNAs can also regulate each other, and therefore they, together with their target genes, form a complex TF-miRNA regulatory network. Recent research has investigated the regulatory rules between miRNAs and genes, and found, for example, that miRNAs preferentially regulate genes that have a high transcriptional regulation complexity [20] and that preferentially target downstream genes in cellular signaling flows [21]. These results support the concept that miRNA, TFs and their target genes form a complex network that enables the cell to conduct a wide range of biological functions. In light of this, studying miRNA evolution within the framework of cellular networks is essential.

At the molecular level, the topology of cellular networks places constraints on protein evolution and introduces functional innovations that open the door for protein evolution. The evolution of the protein-coding genes has been extensively studied in gene transcription [18,22,23], protein interaction [24], cell signaling [25], and metabolic [26] networks. These studies have led to several major conclusions: (1) Hub proteins, that is, proteins that have many interacting links, tend to be more conserved [18,24,25]. (2) Proteins in the network periphery tend to evolve more rapidly whereas those in the network center are more conserved [25]. (3) Network proteins appear to have coevolved with their neighbors in a signaling network [25]; whereas transcription factors tended to evolve independently of their targets in prokaryotic transcriptional regulatory networks [23]; and so on. However, whether and how the architectural structure of cellular networks places constraints on and provides functional innovations for miRNA evolution is unknown. Did TFs coevolve with their target miRNAs and their target protein-coding genes? How does the evolution of miRNA-target pairs occur? Are the evolutionary rates of upstream regulators and downstream regulators different? Each of these questions is of critical importance not only for understanding evolution itself but also for related areas, such as the prediction of miRNA and TF targets.

To address these questions, we compared the evolution of miRNA and protein-coding genes in a manually curated TF-miRNA regulatory network. We used experimentally determined regulatory relations among TFs, miRNAs, and their targets to construct a human TF-miRNA regulatory network, which contains 2,273 nodes, of which 425 and 150 are TF and miRNA nodes, respectively. The network contains 4298 regulatory relations, including 2655 TF-gene regulatory relations, 210 TF-miRNA regulatory relations, and 1433 miRNA-target regulatory relations. We then performed a systems-level analysis to compare the evolutionary patterns of miRNAs and protein-coding genes in the network.

## Results and Discussion

### miRNAs preferentially coevolve with their activators but not with their inhibitors

**Figure 1:**
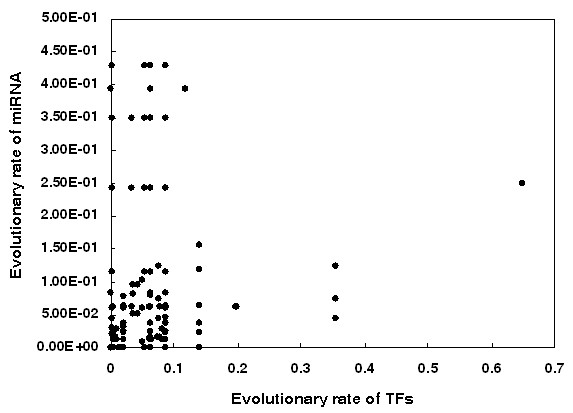
**Coevolutionary analysis of TF-activating-miRNA pairs**. Each dot in the scatter plot represents one TF-activating-miRNA pair. The X axis and Y axis represent the evolutionary rate of TFs and miRNAs, respectively.

**Figure 2:**
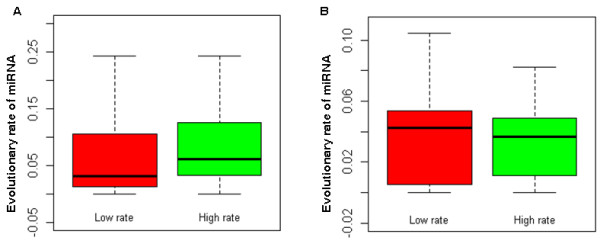
**Coevolutionary patterns of TFs and miRNAs**. (A) For TF-miRNA regulatory pairs connected by transcriptional activation signals, TFs with a low evolutionary rate preferentially regulate miRNAs with a low evolutionary rate, and vice versa. (B) For TF-miRNA regulatory pairs connected by transcriptional repression signals, no difference was found between the evolutionary rates of miRNAs regulated by TFs with a low evolutionary rate and those with a high evolutionary rate.

**Figure 3:**
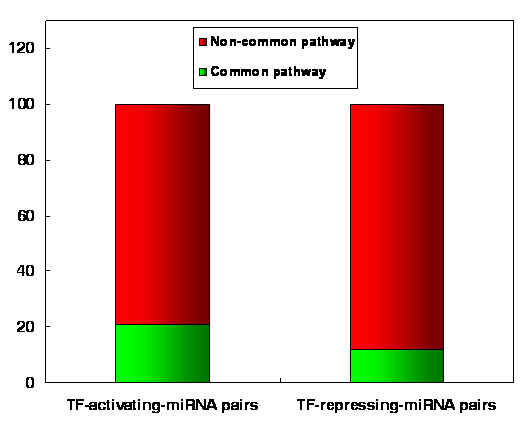
**Distribution of common signaling pathways and non-common signaling pathways regulated by TF-miRNA pairs**. TF-activating-miRNAs pairs prefer to regulate common signaling pathways compared with TF-repressing-miRNA pairs.

In the cellular signaling network two genes which interact tend to coevolve during evolution, in order to adapt to each other [25]. That is, for two genes that interact, if one evolves fast, the other will also evolve fast, and vice versa. The reason might be that if changes occur in one gene, a high probability of their interaction remaining unchanged exists only if the other gene has a corresponding change. Furthermore, different signal types (i.e., activation, inhibition, and physical interaction) in the human cellular signaling network contribute differently to the coevolution of two interacting genes [25]. In light of this, we explored the coevolutionary rules of miRNAs and TFs in the human TF-miRNA regulatory network. We obtained evolutionary rate data of TFs from H-InvDB database and calculated the evolutionary rates of miRNAs based on the pairwise alignment data for humans (hg18) and Rhesus monkeys (rheMac2) from UCSC [27] using Liang et al.'s method [16]. We found evidence of coevolution between TFs and miRNAs (R = 0.19, P = 0.004, Spearman's correlation; Figure 1A). Furthermore, surprisingly, we found that this coevolution between TFs and miRNAs only exists in TF-miRNA pairs that are connected by transcriptional activation signals (R = 0.18, P = 0.026) but not in pairs that are connected by transcriptional repression signals (R = -0.08, P = 0.609). We further classified the TF-miRNA pairs connected by transcriptional activation signals into two equal groups based on the evolutionary rate of their TFs: a low evolutionary rate group and a high evolutionary rate group. We found that the evolutionary rate of miRNAs in the low evolutionary rate group is lower than that of miRNAs in the high evolutionary rate group (median miRNA evolutionary rate: 0.031 vs. 0.061, P = 0.05, Wilcoxon test, Figure 2A). However, we did not find a significant result for the TF-miRNA pairs that are connected by transcriptional repression signals (median miRNA evolutionary rate: 0.042 vs. 0.037, P = 0.5, Wilcoxon test, Figure 2B). This finding indicates that transcriptional activation signals and repression signals contribute differently to the coevolution of TFs and miRNAs. TFs which are activators of miRNAs would trigger miRNA expression and then the TFs and the activated miRNAs could work together to regulate common pathways. We confirmed this by analyzing the signaling pathways that regulated by these TFs and miRNAs. We first obtained 183 human signaling pathways that we previously used in our various studies [21,28,29]. We classified the TF-miRNA pairs into two groups according the signal type: activating group and repressing group. As expected, the TF-miRNA pairs in the activating group have greater probability to regulate common signaling pathways than the TF-miRNA pairs in the repressing group (P = 1.28 × 10^-9^, Fisher's exact test; Odds Ratio (OR) = 1.94). For example, 21% (349/1676) of the TF-miRNA pairs in the activating group regulate the common signaling pathways; whereas only 12% (121/1014) of the TF-miRNA pairs in the repressing group regulate the common signaling pathways (Figure 3). On the other hand, if a miRNA is repressed by a TF, this miRNA would not function along with that TF. Therefore, it is reasonable to think that miRNAs would coevolve with their activators to adapt and response to stimuli. Furthermore, these results indicate that the specific genes involved in gene regulations at the transcriptional level and post-transcriptional level are closely synchronized, specifically they collaborate to have coevolved and have adapted together.

We next investigated whether the signal type in the network contributes differently to the coevolution of TFs and protein-coding genes. Because the types of signal data of regulatory relationship between TFs and protein-coding genes are not available, we used the correlation coefficients of expression profiles of TFs and their target genes as an estimate of the signal type. A positive correlation may suggest an activating regulation; whereas a negative correlation may suggest a repressing regulation. As a result, the evolutionary rates of TF-gene pairs that have positive correlated expression profiles show positive correlation (R = 0.09, P = 0.017, Spearman's correlation), suggesting that TF-activating-gene pairs tend to show coevolution. As a comparison, TF-gene pairs that have negative correlated expression profiles do not show correlated evolutionary rates (R = -0.06, P = 0.249, Spearman's correlation).

### Rapidly evolving TFs frequently activate but rarely repress miRNAs

We next asked whether there is a signal preference when TFs regulate miRNAs. We first classified TF-miRNA regulatory signals into two groups according to the evolutionary rates of their TFs and counted the numbers of activation signals and repression signals in these two groups. We found that 85.4% (76/89) of the signals in the high evolutionary rate group are activation signals, whereas this percentage decreases to 71.9% (82/114) in the low evolutionary rate group (P = 0.016, Fisher's exact test), suggesting that the signals are significantly unevenly distributed in these two groups. These findings indicate that rapidly evolving TFs are preferentially involved with transcriptional activation signals in TF-miRNA regulations.

We wondered whether a similar trend exists for the transcription regulation of protein-coding genes. Shinar et al. reported that protein-coding genes that are frequently needed in the natural environment tend to be activated but rarely needed genes tend to be repressed [30]. In addition, broadly expressed protein-coding genes are more conserved than those with a narrow expression profile [31]. Taken together, these findings indicate that conserved protein-coding genes tend to be activated by TFs and rapidly evolving protein-coding genes tend to be repressed. Because the signal type data of the protein-coding genes is not available, we were unable to test this trend in the human TF-miRNA regulatory network. When we used the estimated TF-gene regulatory signal type data from expression profile to perform this analysis, we did not obtained significant result (data not shown). However, it will be interesting to confirm this hypothesis when regulatory signal type data of human TF-gene regulations becomes available in the future.

### Rapidly evolving and slowly evolving miRNAs tend to regulate slowly evolving and rapidly evolving protein-coding genes, respectively

For miRNA-gene regulatory pairs, we found that conserved miRNAs seem to regulate rapidly evolving protein-coding genes, whereas rapidly evolving miRNAs seem to regulate conserved protein-coding genes. We classified miRNA-target pairs into two equal groups according to the evolutionary rate of the miRNAs. We found that targets in the low rate group had a higher evolutionary rate than those in the high rate group (median dN: 0.052 vs. 0.046, P = 0.02, Wilcoxon test). To understand this phenomenon, we took into account the expression of miRNAs and their target genes. More conserved protein-coding genes tend to have a higher expression level and a larger breadth of expression [31]. Recently Liang and Li reported a similar rule for miRNAs [16]. Because miRNAs negatively regulate their target genes, broadly expressed (conserved) genes cannot be regulated by broadly expressed (conserved) miRNAs. If broadly expressed genes (that is, genes that are expressed in many tissues) are repressed by broadly expressed miRNAs, the broadly expressed protein-coding genes could be repressed. Thus, these broadly expressed genes could not continue to be broadly expressed. Therefore, broadly expressed genes tend to be regulated by tissue-specific miRNAs, which tend to evolve more rapidly.

In contrast, the coevolution between TF-protein-coding gene regulatory pairs is not significant (data not shown), which is consistent with the result of Madan Babu et al. [23].

### Upstream miRNAs evolve more rapidly than downstream miRNAs, whereas upstream TFs are more conserved than downstream TFs

**Figure 4:**
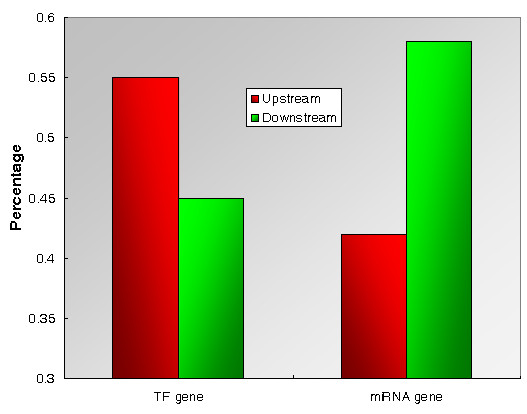
**Distribution of evolutionary rates of TFs and miRNAs along the gene regulatory cascade**. The Y axis represents the percentage of upstream regulators (TF genes or miRNA genes) that are more conserved than downstream regulators (red bar) and the percentage of downstream regulators that are more conserved than upstream regulators (green bar).

We previously reported that in the cellular signaling flow the upstream nodes are less likely to be conserved whereas downstream nodes are more likely to be conserved [25]. In light of this, we investigated the evolutionary patterns of TFs and miRNAs, the two classes of gene regulators in the TF-miRNA regulatory network, along the regulatory cascade. We found that the upstream TFs were more conserved than the downstream TFs; whereas the upstream miRNAs evolved more rapidly than the downstream miRNAs. Of the 4688 TF pairs, 60% have a lower evolutionary rate in the upstream nodes; whereas only 42% (431/1045) of the miRNA pairs have a lower rate in the upstream nodes (Figure 4, P = 5.3×10^-26^, Fisher's exact test).

This result indicates that the two classes of gene regulators, TFs and miRNAs, show opposite patterns in evolutionary rates in the upstream and downstream of the regulatory cascade. The upstream TFs would seem to play more critical roles in the gene regulatory network than do downstream TFs because they not only regulate genes but also regulate other regulators (TFs and miRNAs). They tend to be more essential and therefore are more conserved than the downstream TFs because essential genes tend to more conserved, as Pal indicated [31].

On the other hand, miRNAs regulate genes by repressing them. The repressing function may have a systems-level function of buffering gene expression noise. For example, we previously showed that miRNAs buffer gene expression noise between species and thus buffer the evolution of the species [15]. Wu and colleagues confirmed this function [32]. In biological systems such as cell signaling it is desirable to filtering out noisy signals in the upstream region where genes are responding to a broad range of extracellular stimuli. An integrative analysis of the regulation of a human signaling network by miRNAs suggests that miRNAs could filter noisy signals in the upstream region of the signaling network [21]. Furthermore, Legewie et al. suggested that negative feedbacks may serve as major regulatory loops in the upstream region of the signaling networks [33]. Considering all of these aspects of miRNAs as native regulators, it is reasonable that upstream miRNAs evolve more rapidly than downstream miRNAs. Rapidly evolving miRNAs in the upstream of a regulatory cascade could allow adapt the cell to environmental changes and tone down the signaling process, as suggested by experimental studies in which signaling persisted if transcriptional feedback by proteins was blocked by protein biosynthesis inhibitors [34]. More importantly, rapidly acting post-translational feedbacks may frequently be important for initial signal processing and specificity [29,35]. Therefore, rapidly evolving miRNAs in the upstream of regulatory cascades allow the system to adapt in ways that allow for filtering out noisy signals and controlling the processing and specificity of the original signal.

### Sensitivity analysis

A common limitation of biological network analysis is that currently all reported biological networks are far from completeness. Therefore, the observations especially the observations that are not very significant from biological network analysis may be resulted from data incompleteness. In this case, sensitivity analysis is often used to solid the findings[25]. In this study, the constructed human TF-miRNA regulatory is also far from completeness. To solid this study, we performed sensitivity analysis for results that are not very significant. For each analysis, we randomly removed 5% true links, added 5% false links at the same time, and repeated the analysis. As a result, the main results remain unchanged (Table 1).

**Table 1:** Results of sensitivity analysis

Analysis	Results
Coevolution of TF-miRNA	R = 0.19, P = 0.003, Spearman's correlation

Coevolution of TF-activating-miRNA	R = 0.20, P = 0.01, Spearman's correlation

Coevolution of TF-repressing-miRNA	R = -0.04, P = 0.77, Spearman's correlation

Coevolution of TF-activating-gene	R = 0.08, P = 0.05, Spearman's correlation

Coevolution of TF-repressing-gene	R = -0.06, P = 0.27, Spearman's correlation

Evolutionary rates of targets regulated by rapidly evolving and slowly evolving miRNAs, respectively	Median dN 0.047 vs. 0.051, P = 0.05, Wilcoxon test

## Discussion

In summary, we have uncovered that the architectural structure of the TF-miRNA regulatory network provides constraints and functional innovations for miRNA evolution. Moreover, miRNAs have unique and even opposite evolutionary rules comparing with TFs and other protein-coding genes, suggesting that miRNAs may have unique functions and roles in various biological processes and diseases.

The most interesting discovery from this study is that the evolutionary patterns of miRNAs are different from those of the TFs and other protein-coding genes at the network level. For example, in the regulatory cascades, upstream miRNAs evolve more rapidly than downstream miRNAs, whereas upstream TFs are more conserved than downstream TFs. Most of the miRNAs are negative regulators. Taking both of these facts into consideration, we concluded that rapidly-evolving miRNAs in the upstream of the regulatory cascades allow the system to adapt in ways that allow the cell to filter out noisy signals and control the processing and specificity of the original signal. This conclusion supports the concept that miRNAs have buffers on their expression at the systems-level [32]. Similarly, rapidly evolving and slowly evolving miRNAs tend to regulate slowly evolving and rapidly evolving protein-coding genes, respectively In addition, rapidly evolving TFs tend to activate miRNAs but tend to repress protein-coding genes. On the other hand, TFs and miRNAs have not evolved independently. For example, miRNAs preferentially coevolve with their activators (TFs), and rapidly evolving TFs preferentially activate miRNAs.

In addition, our findings will also be valuable for other fields, such as miRNA target prediction. We revealed that conserved miRNAs tend to avoid regulating conserved targets. This observation is helpful in designing better principles for the prediction of miRNA targets. Finally, the TF-miRNA regulatory network we presented in this study represents the first TF-miRNA regulatory network and will be a valuable platform of systems biology.

## Conclusions

In this study, we performed an analysis of miRNA evolution in a human TF-miRNA regulatory network, which integrated the experimentally supported regulatory relations of TF-miRNA, TF-target, and miRNA-target. This network represents the first large-scale human TF-miRNA regulatory network. As a result, some principles and patterns of miRNA evolution in the human TF-miRNA regulatory network have been uncovered. These results are helpful for not only the understanding of miRNA origin, evolution, and function but also the development of novel methods for miRNA bioinformatics, for example the prediction of miRNA targets.

## Methods

### Construction and analysis of the human TF-miRNA regulatory network

We constructed a human TF-miRNA regulatory network based on experimentally supported regulatory relations between TFs and genes, between TFs and miRNAs, and between miRNAs and targets. We obtained the experimentally supported human TF-gene regulatory relations from TransFac (TRANSFAC Professional version, January 2009, http://www.gene-regulation.com) and the experimentally supported human miRNA-target regulatory relations from TarBase [36]. In order to obtain experimentally supported TF-miRNA regulatory relations, we manually curated ~5000 papers published before April 2009 and obtained experimentally supported TF-miRNA regulatory relations http://cmbi.bjmu.edu.cn/transmir[37]. We next constructed a human TF-miRNA regulatory network using the above three types of regulatory relations among TFs, miRNAs, and target genes (Additional file 1). Of the TF-miRNA links, activating links is greatly more than, repressing links. This result indicates that, for the transcription of miRNAs, activation interactions are more common than repression interactions, a finding which is consistent with the observation for the transcription of protein-coding genes [38]. Normally miRNAs act as negative gene regulators and all of the miRNA-target links identified in this study are indeed negative. The network nodes have a skewed degree distribution. Specifically, most of the TFs regulate just a few miRNAs, but some TFs regulate many miRNAs. For example, the TF that regulats the largest number of miRNAs in this network, MYC, regulates 26 miRNAs.

We implemented a Java program to identify the components of this network (Additional file 2). The resulting network contains 29 network components, of which the largest network component contains 97% of the network nodes. This finding suggests that TFs, miRNAs, and target genes intereact in a single, closely interconnected TF-miRNA-target regulatory network. We identified upstream and downstream TFs/miRNAs using shortest paths, which is obtained by Dijkstra's algorithm.

### Evolutionary rate data of human genes and miRNAs

In this study, we used dN as a measure of the evolutionary rate of protein-coding genes [39] and used the miRNA sequence divergence as the measure of the evolutionary rate of miRNAs [16]. We downloaded the human-mouse protein dN data (Additional file 3) from the H-InvDB database http://jbirc.jbic.or.jp/hinv/dataset/download.cgi. In order to calculate the evolutionary rates for human miRNAs, we first downloaded the pairwise alignment data for humans (hg18) and Rhesus monkeys (rheMac2) from UCSC [27]. We next obtained the genome coordinates data for known human miRNAs from miRBase. We then calculated the sequence divergence for human miRNAs (Additional file 4) using the method presented by Liang et al [16].

### Statistical computing

We performed a Spearman's correlation test, Wilcoxon test, and Fisher's exact test using R, a statistical computing language http://www.r-project.org/.

## Authors' contributions

QC designed the study. CQ, JW, and PY performed the analysis. QC and EW wrote the manuscript. All authors read and approved the final version of the manuscript.

## Supplementary Material

Additional file 1 **The human TF-miRNA regulatory network This file contains the data of the human TF-miRNA regulatory network**. Three types of regulatory relationships are presented. They are the TF-gene regulatory links, the TF-miRNA regulatory links, and the miRNA-target regulatory links. The three types of regulatory links of the human TF-miRNA regulatory network are listed as follows. Click here for file

Additional file 2 **Source code for the identification of network components**. This file contains the java source codes for network components identification. Click here for file

Additional file 3 **Evolutionary rates of protein-coding genes**. This file contains the evolutionary rates of protein-coding genes. Click here for file

Additional file 4 **Evolutionary rates of miRNA genes**. This file contains the evolutionary rates of miRNA genes. Click here for file
