# Supplementary material for: microRNA evolution in a human transcription factor and microRNA regulatory network
Source: BMC Syst Biol. 2010 Jun 29;4:90. doi: 10.1186/1752-0509-4-90 (PMC2914650; doi:10.1186/1752-0509-4-90)
Supplement: Additional file 1 — The human TF-miRNA regulatory network This file contains the data of the human TF-miRNA regulatory network. Three types of regulatory relationships are presented. They are the TF-gene regulatory links, the TF-miRNA regulatory links, and the miRNA-target regulatory links. The three types of regulatory links of the human TF-miRNA regulatory network are listed as follows. [file 1752-0509-4-90-S1.DOC]

**Additional file 1.** This file contains the data of the human TF-miRNA regulatory network. Three types of regulatory relationships are presented. They are the TF-gene regulatory links, the TF-miRNA regulatory links, and the miRNA-target regulatory links. The three types of regulatory links of the human TF-miRNA regulatory network are listed as follows.

TF-gene regulatory links:

EntrezID of TF EntrezID of gene

142 2919

142 6622

190 6770

196 1543

196 2353

196 3315

196 7031

328 5741

367 354

367 367

367 1026

367 2158

367 3936

367 5284

401 1621

401 6530

401 7857

405 1356

405 2056

405 2353

405 2597

405 3280

405 3398

405 3952

405 4846

405 5045

405 5209

405 7018

405 7422

405 23621

405 51107

405 54578

406 5054

463 174

463 290

466 595

466 598

466 672

466 890

466 909

466 1595

466 1958

466 2064

466 2934

466 3811

466 4014

466 4261

466 5972

466 6750

466 7003

466 7042

466 57630

467 467

467 4312

467 6401

467 7157

467 53947

468 6955

604 598

604 894

604 7157

639 3399

639 3456

639 4261

639 5079

639 6689

672 1027

672 7015

860 632

860 860

860 1178

860 1300

860 2263

860 2778

860 4322

860 4982

860 7015

860 9421

860 50964

860 57708

861 596

861 3562

861 5328

861 6348

861 27040

861 29949

864 3804

864 3805

864 115653

1044 6476

1044 27035

1045 596

1045 2984

1045 4583

1045 4585

1045 5891

1045 6476

1045 9075

1045 27035

1045 54575

1045 54576

1050 241

1050 336

1050 338

1050 596

1050 1071

1050 1401

1050 1435

1050 1553

1050 1576

1050 2052

1050 2158

1050 3383

1050 3586

1050 3713

1050 4057

1050 5054

1050 5468

1050 5710

1050 6280

1050 7018

1050 9370

1050 54106

1050 407008

1051 596

1051 597

1051 1026

1051 1401

1051 1553

1051 1594

1051 2155

1051 2263

1051 3239

1051 3383

1051 3553

1051 3569

1051 3576

1051 3586

1051 3949

1051 4210

1051 5054

1051 5243

1051 5618

1051 6280

1051 6289

1051 6348

1051 6352

1051 6573

1051 6690

1051 7130

1051 7150

1051 7923

1051 8795

1051 10068

1051 55553

1052 241

1052 1080

1052 1553

1052 3569

1052 5054

1052 6352

1053 241

1053 1435

1053 2155

1054 2155

1316 4843

1316 7980

1385 154

1385 595

1385 598

1385 627

1385 672

1385 890

1385 909

1385 985

1385 1080

1385 1392

1385 1595

1385 1621

1385 1831

1385 1958

1385 2064

1385 2335

1385 2353

1385 2798

1385 3397

1385 3458

1385 3484

1385 3630

1385 3811

1385 4014

1385 4286

1385 4929

1385 5250

1385 5327

1385 5663

1385 5741

1385 5972

1385 6134

1385 6352

1385 6594

1385 6662

1385 6750

1385 7124

1385 7178

1385 7200

1385 7432

1385 9156

1385 9592

1385 10795

1385 26834

1385 54106

1385 55851

1385 55872

1385 406921

1386 467

1386 595

1386 890

1386 909

1386 1386

1386 1583

1386 1584

1386 1958

1386 2335

1386 2353

1386 3456

1386 3458

1386 3630

1386 4313

1386 5327

1386 5328

1386 6352

1386 6401

1386 7042

1386 7124

1386 7432

1386 53947

1386 55872

1387 3456

1387 3665

1390 595

1390 890

1390 909

1390 2353

1390 3630

1482 4878

1482 5351

1489 5921

1489 7157

1523 1536

1642 5610

1643 5610

1649 8795

1869 595

1869 596

1869 857

1869 890

1869 898

1869 990

1869 993

1869 1029

1869 1031

1869 1537

1869 1719

1869 1846

1869 1869

1869 1870

1869 1876

1869 3925

1869 4217

1869 4609

1869 4616

1869 4998

1869 5046

1869 5800

1869 5933

1869 6670

1869 7015

1869 7161

1869 8600

1869 9077

1869 10926

1869 55872

1869 89845

1869 406952

1870 4217

1871 1031

1871 4217

1871 9077

1874 983

1874 1031

1874 3925

1874 4217

1874 5111

1874 9077

1875 9077

1876 7015

1879 973

1879 3543

1879 7441

1943 675

1943 919

1943 968

1943 2205

1943 2207

1943 2242

1943 3558

1943 3559

1943 3811

1943 4067

1943 4790

1943 27040

1958 20

1958 356

1958 595

1958 847

1958 1234

1958 1278

1958 1280

1958 1636

1958 1647

1958 1956

1958 2002

1958 2152

1958 2247

1958 2321

1958 3397

1958 3558

1958 3560

1958 3562

1958 3949

1958 4129

1958 4665

1958 4790

1958 5154

1958 5468

1958 5979

1958 6549

1958 6550

1958 6647

1958 6720

1958 7048

1958 7124

1958 7803

1958 8061

1958 9536

1958 10397

1958 56034

1959 2705

1959 5243

1960 356

1961 3558

1961 7124

1998 4067

1998 5328

1999 284

1999 326

1999 1366

1999 6699

1999 7048

2002 1959

2002 2002

2002 2353

2002 4846

2002 5663

2002 6722

2002 7124

2002 8645

2002 54106

2004 2353

2005 2353

2033 595

2033 3456

2033 3486

2033 3665

2034 2597

2034 4846

2063 344

2063 345

2063 3973

2099 177

2099 348

2099 595

2099 598

2099 672

2099 901

2099 1026

2099 1393

2099 1442

2099 1545

2099 1728

2099 1831

2099 2353

2099 3206

2099 3315

2099 4057

2099 4313

2099 4582

2099 5241

2099 5757

2099 7015

2099 7018

2099 7022

2099 7031

2099 7039

2099 7124

2099 7157

2099 7422

2099 9166

2099 9167

2099 9368

2099 10553

2099 54828

2099 317716

2100 1393

2100 1442

2100 1728

2100 3643

2100 6752

2100 7031

2100 7124

2100 7422

2100 10553

2101 4057

2113 285

2113 326

2113 719

2113 834

2113 841

2113 925

2113 963

2113 985

2113 1147

2113 1278

2113 1460

2113 1889

2113 2064

2113 2352

2113 2908

2113 2925

2113 3560

2113 3687

2113 3811

2113 4249

2113 4312

2113 4790

2113 5310

2113 5663

2113 5744

2113 6237

2113 6667

2113 6698

2113 6955

2113 7020

2113 7056

2113 7076

2113 7124

2113 7454

2113 7852

2113 8061

2113 22801

2113 51744

2113 114757

2114 326

2114 595

2114 983

2114 2064

2114 3815

2114 4247

2114 5328

2114 5663

2114 7053

2114 8061

2114 54106

2118 2064

2118 2353

2118 2629

2118 2683

2118 4312

2118 4314

2118 4585

2118 4811

2118 5328

2118 9334

2119 3383

2119 4312

2119 4314

2119 5663

2122 7704

2130 57630

2161 1536

2254 2

2254 2209

2254 2353

2254 2633

2254 7453

2254 8554

2254 9636

2295 6439

2305 259

2305 338

2305 1401

2305 1576

2305 6439

2305 57708

2308 2538

2308 3484

2308 5166

2309 356

2309 367

2309 1027

2309 1831

2309 2538

2309 3397

2309 3484

2309 4843

2309 5166

2309 7042

2309 8660

2309 8743

2309 10935

2309 57708

2313 968

2313 1278

2313 1958

2313 2353

2313 2815

2313 5310

2313 51206

2353 9

2353 350

2353 595

2353 719

2353 826

2353 1621

2353 1906

2353 2006

2353 2312

2353 2353

2353 2771

2353 2798

2353 3558

2353 3576

2353 3868

2353 4312

2353 4313

2353 4321

2353 4502

2353 4988

2353 5241

2353 5328

2353 7157

2353 7465

2353 8061

2353 10628

2353 53947

2353 57708

2353 124056

2354 8797

2355 672

2355 1442

2355 3567

2355 3713

2355 6699

2355 7178

2355 8061

2494 8714

2494 9370

2516 269

2516 1584

2516 1585

2516 1586

2516 3284

2516 4158

2521 5471

2521 10606

2547 540

2551 672

2551 1667

2551 2064

2551 3560

2551 3689

2551 3811

2551 4528

2551 5021

2551 5663

2551 6237

2551 6389

2551 6790

2551 7019

2551 7066

2551 7402

2551 9804

2551 9868

2551 10855

2551 54538

2553 672

2553 1667

2553 3560

2553 3689

2553 4528

2553 5021

2553 5663

2553 7066

2553 7402

2553 10855

2553 54538

2623 212

2623 246

2623 350

2623 1536

2623 2056

2623 2057

2623 2994

2623 2995

2623 3133

2623 3145

2623 3212

2623 3674

2623 3685

2623 4005

2623 4904

2623 5553

2623 5981

2623 6006

2623 6708

2623 7490

2623 10210

2623 51206

2623 727897

2624 350

2624 1536

2624 1906

2624 2056

2624 2057

2624 2212

2624 2798

2624 3567

2624 4846

2624 4904

2624 5553

2624 6403

2624 7490

2624 30816

2625 925

2625 1536

2625 2056

2625 2212

2625 2798

2625 2994

2625 3565

2625 3567

2625 3596

2625 3821

2625 4582

2625 6955

2625 7432

2625 10210

2625 30816

2626 1278

2626 1586

2626 2052

2626 3283

2626 3567

2626 4585

2627 1528

2627 3172

2627 4585

2627 7032

2627 27035

2637 3569

2672 8328

2735 596

2735 894

2735 2735

2735 3489

2735 3728

2735 5727

2736 596

2736 2735

2737 596

2737 2735

2737 5727

2908 100

2908 174

2908 632

2908 1392

2908 1559

2908 2908

2908 6744

2908 9970

2908 54658

2909 2908

2969 2353

2971 6895

2971 23621

3065 1026

3065 3397

3065 3486

3065 7148

3068 150572

3091 1050

3091 1356

3091 2056

3091 2597

3091 3398

3091 3952

3091 4846

3091 4879

3091 4915

3091 5045

3091 5209

3091 7015

3091 7018

3091 7422

3091 7852

3091 23621

3091 51107

3096 3283

3096 3284

3096 3456

3096 4609

3148 5243

3159 2919

3159 3456

3159 3484

3159 3559

3159 3643

3159 5243

3164 230

3164 1585

3164 1869

3164 2908

3164 5054

3169 259

3169 338

3169 2052

3169 3239

3169 3484

3169 4585

3169 7018

3169 317716

3170 259

3170 335

3170 338

3170 1551

3170 2052

3170 3484

3170 3651

3170 5627

3171 1576

3172 183

3172 259

3172 335

3172 337

3172 338

3172 344

3172 345

3172 632

3172 1109

3172 1558

3172 1559

3172 1565

3172 1576

3172 1582

3172 1593

3172 2056

3172 2155

3172 2160

3172 2161

3172 2169

3172 2353

3172 2710

3172 2984

3172 3172

3172 3990

3172 4477

3172 5265

3172 5465

3172 6462

3172 6580

3172 6927

3172 10864

3172 27330

3172 57144

3172 64240

3172 64241

3174 632

3174 1109

3175 3172

3178 5610

3190 1977

3190 4609

3190 6714

3202 5241

3202 5764

3202 6530

3205 6401

3206 1026

3206 1536

3206 2018

3206 3690

3206 9935

3211 1290

3217 2247

3235 3235

3236 3235

3280 429

3280 2548

3280 5730

3280 5978

3297 3308

3297 5243

3298 3308

3298 8600

3344 3558

3394 968

3394 1536

3394 9636

3484 3456

3516 602

3516 1401

3516 3456

3516 3569

3516 4790

3516 4792

3553 10553

3569 3308

3569 10553

3586 7076

3592 3558

3593 3558

3603 7130

3607 3558

3621 174

3642 3642

3651 3630

3659 356

3659 632

3659 841

3659 1278

3659 1520

3659 1536

3659 2908

3659 3135

3659 3433

3659 3439

3659 3456

3659 3586

3659 3620

3659 3669

3659 4261

3659 5698

3659 6352

3659 6363

3659 6590

3659 6890

3659 7098

3659 7412

3659 8519

3659 8554

3659 9636

3659 10068

3659 10392

3660 356

3660 1278

3660 1520

3660 1536

3660 2908

3660 3456

3660 3586

3660 4261

3660 7098

3661 3439

3661 3456

3661 3665

3661 6352

3661 6363

3661 9636

3662 968

3662 1536

3662 3565

3662 3662

3662 9636

3663 3439

3663 3448

3663 6363

3665 3439

3665 3448

3665 3456

3665 3665

3665 6352

3665 6363

3665 9636

3725 9

3725 12

3725 226

3725 350

3725 351

3725 467

3725 595

3725 597

3725 623

3725 672

3725 719

3725 826

3725 1277

3725 1437

3725 1442

3725 1576

3725 1621

3725 1636

3725 1906

3725 2006

3725 2152

3725 2247

3725 2312

3725 2335

3725 2353

3725 2629

3725 2670

3725 2697

3725 2729

3725 2798

3725 2937

3725 2950

3725 3145

3725 3159

3725 3239

3725 3456

3725 3458

3725 3558

3725 3562

3725 3567

3725 3576

3725 3687

3725 3713

3725 3725

3725 3732

3725 3868

3725 4014

3725 4092

3725 4144

3725 4312

3725 4313

3725 4314

3725 4318

3725 4321

3725 4436

3725 4502

3725 4609

3725 4843

3725 4852

3725 4878

3725 5021

3725 5055

3725 5241

3725 5328

3725 5973

3725 6347

3725 6351

3725 6352

3725 6401

3725 6520

3725 6670

3725 6698

3725 6699

3725 6700

3725 6707

3725 6775

3725 6916

3725 7015

3725 7031

3725 7040

3725 7051

3725 7054

3725 7076

3725 7124

3725 7130

3725 7157

3725 7252

3725 7422

3725 7432

3725 8797

3725 10628

3725 10666

3725 53947

3725 57708

3725 124056

3726 3239

3726 3558

3726 3567

3726 3713

3726 5328

3726 6698

3726 6699

3726 7051

3726 8061

3726 8797

3727 595

3727 1442

3727 1728

3727 2064

3727 2312

3727 2729

3727 2730

3727 3239

3727 3567

3727 3569

3727 3713

3727 4835

3727 5328

3727 6699

3727 7015

3727 7051

3727 8061

3727 8797

4005 3815

4010 1286

4010 7827

4066 3397

4086 7538

4087 7042

4087 10516

4088 351

4088 1278

4088 1294

4088 2006

4088 2022

4088 3725

4088 4092

4088 4312

4088 5054

4088 5744

4088 7015

4088 7042

4088 7422

4088 10516

4089 351

4089 1278

4089 2006

4089 2022

4089 3725

4089 4092

4089 4312

4089 5054

4089 5268

4089 5744

4089 7042

4089 7422

4094 2771

4097 350

4097 2729

4097 2730

4149 993

4149 4904

4149 7015

4150 4150

4150 4609

4150 5334

4150 5745

4152 4100

4152 4102

4152 4111

4152 266740

4205 2027

4205 4656

4208 1375

4208 53630

4211 5196

4286 596

4286 1638

4286 2315

4286 4157

4286 4233

4286 4935

4286 6490

4286 7299

4286 7306

4286 7439

4286 79444

4303 604

4303 3484

4303 5934

4335 7015

4520 2729

4520 3308

4520 4502

4602 100

4602 983

4602 2908

4602 2950

4602 3815

4602 4602

4602 6427

4602 6670

4602 7201

4609 993

4609 1019

4609 1977

4609 2064

4609 2697

4609 4609

4609 4904

4609 7015

4609 7852

4609 8886

4609 89874

4609 406952

4609 727897

4613 4193

4613 5747

4613 727897

4617 23209

4618 23209

4654 208

4654 2353

4654 6744

4654 23209

4654 50964

4656 23209

4763 246

4763 338

4763 2335

4763 2670

4763 2796

4763 3925

4763 7020

4763 7157

4763 8641

4772 356

4772 1437

4772 1960

4772 2208

4772 3458

4772 3558

4772 3562

4772 3565

4772 3690

4773 1437

4773 1960

4773 2208

4773 3383

4773 3558

4773 3565

4773 3662

4773 3690

4773 7124

4774 407008

4775 643

4775 4879

4776 5468

4778 350

4778 3145

4779 1728

4779 2730

4779 2937

4780 158

4780 350

4780 1728

4780 2729

4780 2730

4780 2771

4780 2937

4780 2950

4780 3868

4780 4835

4780 6277

4782 292

4782 350

4782 720

4782 875

4782 1026

4782 1277

4782 1493

4782 1528

4782 1551

4782 1576

4782 1586

4782 2173

4782 3146

4782 3308

4782 3397

4782 3725

4782 4246

4782 4852

4782 5054

4782 5284

4782 6670

4782 6744

4782 7018

4782 8022

4790 355

4790 595

4790 1278

4790 1452

4790 2152

4790 2157

4790 2729

4790 2771

4790 2908

4790 2919

4790 2920

4790 2921

4790 3383

4790 3456

4790 3458

4790 3558

4790 3559

4790 3569

4790 3576

4790 4049

4790 4616

4790 4790

4790 4843

4790 5747

4790 6287

4790 6347

4790 6352

4790 6401

4790 6696

4790 6869

4790 7124

4790 7157

4790 7852

4790 406938

4791 3456

4791 4791

4791 6363

4800 350

4800 875

4800 890

4800 983

4800 1514

4800 1543

4800 1647

4800 1869

4800 2052

4800 2356

4800 4603

4800 5243

4800 5627

4800 6658

4800 7048

4800 7155

4800 7913

4800 10308

4800 10628

4800 10957

4800 50509

4800 51702

4801 478

4801 875

4801 983

4801 1027

4801 1514

4801 2052

4801 2212

4801 2356

4801 2719

4801 4246

4801 4287

4801 4603

4801 5243

4801 6670

4801 7048

4801 8721

4801 10957

4801 50509

4802 10957

4802 50509

4824 118425

4831 4609

4841 5250

4899 350

4899 826

4899 961

4899 1869

4899 1876

4899 1965

4899 5471

4899 6389

4899 7019

4899 9513

4899 9804

4899 10606

4899 64397

4901 6010

4904 355

4904 5243

4904 6532

4929 1585

4929 6531

5013 7306

5036 1869

5074 56616

5076 7157

5077 4286

5079 930

5079 2208

5079 7157

5080 3678

5080 3688

5080 6597

5087 1026

5087 1290

5087 1536

5087 3586

5087 5328

5087 7367

5088 1290

5088 3690

5088 5328

5088 7367

5204 54361

5241 2798

5241 3484

5241 4609

5308 5351

5309 6531

5316 1290

5316 5328

5316 7367

5328 2353

5328 6029

5328 7432

5449 2796

5449 7421

5451 9

5451 350

5451 595

5451 973

5451 3558

5451 3562

5451 3567

5451 3713

5451 4023

5451 4067

5451 4609

5451 6066

5451 8349

5452 3558

5452 4609

5452 8349

5454 230

5454 1644

5454 4671

5463 3558

5465 335

5465 1374

5465 1375

5465 3158

5465 6555

5465 9572

5465 10501

5465 116519

5468 51

5468 3638

5468 4035

5468 9370

5468 53630

5626 5449

5716 3558

5914 126

5914 335

5914 345

5914 632

5914 643

5914 912

5914 4843

5914 5915

5914 5916

5914 6439

5914 7066

5915 34

5915 126

5915 335

5915 5915

5915 5916

5915 5973

5916 912

5916 5915

5916 5916

5916 6439

5925 3397

5925 3815

5933 890

5933 89845

5937 355

5966 597

5966 598

5966 2152

5966 3456

5966 3559

5966 3662

5966 5284

5966 6347

5966 7124

5970 12

5970 177

5970 356

5970 595

5970 596

5970 1278

5970 1535

5970 1734

5970 1999

5970 2152

5970 2771

5970 3037

5970 3091

5970 3135

5970 3383

5970 3456

5970 3458

5970 3460

5970 3553

5970 3559

5970 3569

5970 3576

5970 3662

5970 4144

5970 4155

5970 4210

5970 4276

5970 4791

5970 4929

5970 4973

5970 4988

5970 5284

5970 5747

5970 6347

5970 6352

5970 6363

5970 6775

5970 7097

5970 7124

5970 7157

5970 7412

5970 8795

5970 9641

5970 64127

5970 727897

5971 3662

5971 6363

5978 551

5978 1141

5978 1584

5978 3897

5978 4916

5978 4988

5978 6853

5978 7054

5978 8829

5978 9479

5978 57369

5989 3568

5989 5111

5990 3568

5991 3568

5992 3568

6117 672

6118 672

6119 672

6241 3456

6241 4938

6256 19

6256 34

6256 51

6256 126

6256 211

6256 335

6256 336

6256 344

6256 345

6256 348

6256 570

6256 632

6256 643

6256 912

6256 1026

6256 1071

6256 1374

6256 1375

6256 1551

6256 1555

6256 1557

6256 1558

6256 1559

6256 1576

6256 1956

6256 2172

6256 3158

6256 3484

6256 3638

6256 4035

6256 4609

6256 4843

6256 5243

6256 5360

6256 5467

6256 5777

6256 5915

6256 5916

6256 5973

6256 6439

6256 6517

6256 6555

6256 6658

6256 7099

6256 7124

6256 7361

6256 8647

6256 9370

6256 9572

6256 9619

6256 9965

6256 10062

6256 10501

6256 10599

6256 10998

6256 53630

6256 55503

6256 116519

6304 1536

6421 5250

6591 675

6596 5054

6657 4609

6659 914

6662 916

6663 4286

6667 20

6667 100

6667 135

6667 155

6667 177

6667 226

6667 240

6667 246

6667 328

6667 335

6667 338

6667 348

6667 355

6667 401

6667 478

6667 581

6667 595

6667 598

6667 672

6667 682

6667 688

6667 720

6667 826

6667 841

6667 847

6667 857

6667 875

6667 894

6667 896

6667 929

6667 963

6667 1000

6667 1017

6667 1026

6667 1027

6667 1029

6667 1031

6667 1048

6667 1052

6667 1082

6667 1178

6667 1277

6667 1278

6667 1280

6667 1282

6667 1284

6667 1386

6667 1452

6667 1514

6667 1551

6667 1572

6667 1576

6667 1580

6667 1586

6667 1589

6667 1593

6667 1595

6667 1621

6667 1677

6667 1719

6667 1869

6667 1896

6667 1956

6667 2000

6667 2022

6667 2056

6667 2057

6667 2149

6667 2152

6667 2205

6667 2242

6667 2247

6667 2264

6667 2321

6667 2332

6667 2335

6667 2352

6667 2353

6667 2670

6667 2697

6667 2735

6667 2796

6667 2820

6667 2919

6667 2994

6667 2995

6667 3037

6667 3064

6667 3159

6667 3172

6667 3217

6667 3283

6667 3284

6667 3308

6667 3315

6667 3397

6667 3400

6667 3458

6667 3459

6667 3486

6667 3552

6667 3558

6667 3560

6667 3586

6667 3636

6667 3643

6667 3662

6667 3673

6667 3681

6667 3684

6667 3685

6667 3713

6667 3725

6667 3811

6667 3815

6667 3868

6667 3875

6667 3931

6667 3949

6667 3991

6667 4014

6667 4023

6667 4056

6667 4057

6667 4128

6667 4129

6667 4150

6667 4246

6667 4257

6667 4284

6667 4287

6667 4313

6667 4353

6667 4502

6667 4582

6667 4585

6667 4601

6667 4603

6667 4609

6667 4613

6667 4625

6667 4665

6667 4811

6667 4835

6667 4839

6667 4842

6667 4846

6667 4852

6667 4988

6667 5045

6667 5054

6667 5055

6667 5104

6667 5154

6667 5155

6667 5213

6667 5241

6667 5243

6667 5250

6667 5471

6667 5578

6667 5610

6667 5618

6667 5627

6667 5663

6667 5727

6667 5744

6667 5745

6667 5914

6667 5921

6667 5966

6667 5970

6667 5973

6667 5979

6667 5981

6667 6066

6667 6134

6667 6281

6667 6347

6667 6436

6667 6549

6667 6550

6667 6573

6667 6574

6667 6594

6667 6646

6667 6647

6667 6658

6667 6662

6667 6667

6667 6670

6667 6678

6667 6693

6667 6696

6667 6699

6667 6720

6667 6744

6667 6752

6667 6772

6667 6844

6667 6879

6667 6895

6667 6955

6667 7003

6667 7015

6667 7022

6667 7031

6667 7039

6667 7040

6667 7046

6667 7047

6667 7048

6667 7053

6667 7080

6667 7097

6667 7124

6667 7148

6667 7155

6667 7157

6667 7167

6667 7298

6667 7402

6667 7422

6667 7428

6667 7803

6667 7941

6667 8022

6667 8061

6667 8575

6667 8578

6667 8721

6667 8877

6667 9156

6667 9232

6667 9334

6667 9420

6667 9421

6667 9536

6667 10395

6667 10397

6667 10606

6667 10666

6667 10765

6667 10855

6667 10926

6667 22801

6667 23621

6667 27032

6667 27040

6667 29117

6667 30816

6667 51206

6667 51702

6667 54538

6667 56034

6667 57708

6667 80329

6667 89845

6667 114757

6667 124056

6667 148022

6667 727897

6668 595

6668 826

6668 4842

6668 6752

6668 8660

6670 20

6670 356

6670 401

6670 478

6670 595

6670 598

6670 672

6670 682

6670 720

6670 826

6670 875

6670 1000

6670 1026

6670 1031

6670 1278

6670 1280

6670 1514

6670 1528

6670 1551

6670 1572

6670 1576

6670 1580

6670 1586

6670 1593

6670 1595

6670 1869

6670 2149

6670 2321

6670 2335

6670 2352

6670 2919

6670 3037

6670 3159

6670 3217

6670 3283

6670 3284

6670 3486

6670 3560

6670 3636

6670 3685

6670 3868

6670 3931

6670 3949

6670 4014

6670 4023

6670 4056

6670 4129

6670 4246

6670 4313

6670 4585

6670 4613

6670 4625

6670 4835

6670 4842

6670 4988

6670 5054

6670 5627

6670 5979

6670 6549

6670 6550

6670 6573

6670 6574

6670 6667

6670 6670

6670 6752

6670 6772

6670 7022

6670 7031

6670 7047

6670 7048

6670 7080

6670 7097

6670 7157

6670 7402

6670 7422

6670 7941

6670 8061

6670 8660

6670 8721

6670 9156

6670 9420

6670 9421

6670 9536

6670 10395

6670 10765

6670 10855

6670 22801

6670 27040

6670 51702

6670 57708

6670 80329

6670 89845

6671 20

6671 401

6671 478

6671 595

6671 875

6671 9421

6688 820

6688 968

6688 1536

6688 1667

6688 2064

6688 2114

6688 2205

6688 2242

6688 3553

6688 3662

6688 3674

6688 3684

6688 3689

6688 4689

6688 5777

6688 6886

6688 9636

6688 51206

6688 494336

6688 653361

6689 2242

6720 32

6720 47

6720 835

6720 857

6720 1026

6720 2222

6720 3796

6720 3949

6720 5468

6720 5777

6720 6517

6720 6770

6720 8660

6721 19

6721 949

6721 2222

6721 3949

6721 4547

6721 5468

6721 5652

6721 6476

6721 6770

6721 7351

6722 348

6722 1958

6722 1959

6722 2002

6722 2353

6722 4634

6722 8518

6736 916

6736 6736

6772 12

6772 183

6772 595

6772 942

6772 958

6772 1026

6772 1536

6772 2208

6772 2209

6772 2211

6772 2353

6772 2537

6772 3133

6772 3383

6772 3433

6772 3458

6772 3559

6772 3572

6772 3586

6772 3620

6772 3659

6772 4261

6772 4582

6772 4609

6772 4615

6772 4843

6772 4938

6772 4988

6772 5292

6772 5698

6772 6281

6772 6347

6772 6363

6772 6646

6772 6890

6772 7200

6772 7432

6772 7453

6772 8519

6772 9636

6772 9961

6772 10352

6772 27035

6772 54739

6772 59067

6773 6363

6773 59067

6774 12

6774 332

6774 1026

6774 1154

6774 1401

6774 2353

6774 3082

6774 3458

6774 3559

6774 3572

6774 3586

6774 3659

6774 4582

6774 4609

6774 4615

6774 4988

6774 5292

6774 7200

6774 7291

6774 7432

6774 59067

6775 3458

6775 3559

6775 3659

6775 3662

6775 4609

6775 5292

6775 5551

6775 59067

6776 595

6776 598

6776 894

6776 1154

6776 2209

6776 3458

6776 3559

6776 3659

6776 3662

6776 3811

6776 4615

6776 5292

6776 7453

6776 10352

6777 595

6777 1026

6777 1154

6777 3458

6777 3559

6777 3659

6777 5008

6777 5292

6777 10352

6778 246

6778 2208

6778 2209

6778 3557

6778 3559

6778 3566

6778 3662

6778 4988

6778 6361

6778 10344

6886 3815

6908 212

6908 632

6908 1719

6908 2670

6908 3308

6908 7039

6908 26827

6908 51702

6910 4878

6925 269

6926 6862

6927 39

6927 259

6927 336

6927 338

6927 350

6927 1080

6927 1109

6927 1401

6927 1576

6927 2157

6927 2264

6927 2938

6927 3172

6927 3484

6927 3674

6927 4585

6927 4837

6927 5710

6927 6476

6927 6514

6927 6714

6927 7364

6927 7367

6927 9075

6927 9376

6927 9971

6927 10599

6927 27035

6927 27329

6927 50674

6927 54575

6927 54576

6927 54600

6927 57393

6927 117153

6928 336

6928 338

6928 1401

6928 3484

6928 3674

6928 4585

6928 4837

6928 6476

6928 6696

6928 9376

6928 54576

6928 59272

6929 2353

6929 3815

6929 5777

6932 916

6932 925

6934 4316

6934 4609

6934 6932

6934 8061

6935 3558

6935 7161

6935 406933

6935 406985

7003 4625

7004 3725

7004 4625

7005 3283

7005 4625

7005 6744

7020 133

7020 246

7020 338

7020 351

7020 632

7020 891

7020 1050

7020 1082

7020 1386

7020 1583

7020 2064

7020 2264

7020 2332

7020 2670

7020 2796

7020 3064

7020 3315

7020 3458

7020 3480

7020 3488

7020 3725

7020 3732

7020 3815

7020 3848

7020 3854

7020 4284

7020 4313

7020 4502

7020 4609

7020 5104

7020 5284

7020 5578

7020 5966

7020 6648

7020 6744

7020 6879

7020 6888

7020 6955

7020 7020

7020 7039

7020 7428

7020 8884

7020 9513

7020 10553

7020 53947

7020 80329

7020 89874

7020 727897

7021 5730

7021 6573

7021 6648

7022 1475

7022 6648

7022 57708

7023 4502

7023 6895

7023 51107

7023 53947

7025 183

7025 240

7025 335

7025 337

7025 345

7025 1026

7025 1375

7025 1565

7025 1585

7025 2056

7025 2158

7025 3381

7025 3973

7025 3990

7025 4057

7025 5915

7025 7018

7026 58

7026 335

7026 337

7026 338

7026 344

7026 345

7026 1585

7026 2158

7026 2908

7026 3172

7027 857

7027 890

7027 990

7027 1031

7027 1719

7027 1870

7027 3925

7027 4609

7027 4998

7027 5046

7027 5933

7027 7161

7030 5054

7033 3456

7067 1555

7067 4609

7067 4843

7067 7252

7068 336

7068 344

7068 1956

7068 7252

7068 116519

7071 3681

7080 6439

7080 7038

7110 999

7110 7094

7132 983

7157 317

7157 324

7157 355

7157 467

7157 581

7157 834

7157 857

7157 1026

7157 1111

7157 1643

7157 1647

7157 1843

7157 1956

7157 2176

7157 2810

7157 2876

7157 3486

7157 3732

7157 4193

7157 4313

7157 4436

7157 5111

7157 5268

7157 5747

7157 5921

7157 6273

7157 6376

7157 7015

7157 7039

7157 7157

7157 7161

7157 8626

7157 8794

7157 8795

7157 9232

7157 10346

7157 10397

7157 22943

7157 27113

7157 27244

7157 55800

7157 63970

7157 64326

7157 94241

7157 407040

7158 672

7161 100

7161 581

7161 1026

7161 7161

7181 226

7181 1271

7181 2056

7181 5915

7182 348

7182 1271

7182 1589

7182 3973

7291 2735

7342 1583

7376 19

7376 348

7376 1071

7376 9619

7376 10062

7391 328

7391 345

7391 351

7391 444

7391 551

7391 595

7391 632

7391 675

7391 875

7391 891

7391 1048

7391 1050

7391 1551

7391 1576

7391 2735

7391 3214

7391 3217

7391 3482

7391 3669

7391 4261

7391 4609

7391 5054

7391 5196

7391 5284

7391 5730

7391 5981

7391 6277

7391 6573

7391 6658

7391 6744

7391 7015

7391 7156

7391 7299

7391 7706

7391 116519

7391 387281

7392 336

7392 345

7392 351

7392 444

7392 551

7392 675

7392 2735

7392 3214

7392 3482

7392 5196

7392 5284

7392 6277

7392 7015

7392 7852

7392 116519

7421 632

7421 1026

7421 1551

7421 1555

7421 1576

7421 2796

7421 3484

7421 4843

7421 5449

7421 5467

7421 55503

7490 1647

7490 2297

7490 3481

7490 5154

7490 6608

7507 5243

7520 540

7520 1543

7528 9

7528 142

7528 355

7528 1029

7528 1071

7528 1178

7528 1437

7528 1536

7528 1621

7528 2205

7528 2353

7528 2548

7528 3217

7528 3283

7528 3284

7528 3397

7528 3444

7528 3458

7528 3811

7528 5111

7528 5178

7528 6770

7528 7057

7528 7156

7528 7157

7528 7913

7528 7919

7528 8200

7528 8518

7528 27032

7545 348

7546 348

7555 4609

7593 350

7593 875

7593 947

7593 1000

7593 4602

7593 4842

7593 4846

7593 6573

7629 4842

7629 6888

7702 4842

7702 6888

7702 26827

7707 4314

7707 6772

7709 1026

7716 1906

7716 3562

7727 5155

7849 7157

7975 1728

8061 2950

8061 3239

8061 3558

8061 3569

8061 3576

8061 3713

8061 4312

8061 4313

8061 5328

8061 6699

8061 8061

8061 8797

8091 597

8187 5949

8202 2064

8379 7015

8462 350

8462 3630

8462 4129

8463 1956

8538 796

8626 182

8626 1026

8626 4193

8648 2064

8648 3206

8844 4014

8856 211

8856 1551

8856 1555

8856 1557

8856 1558

8856 1559

8856 1576

8856 4843

8856 5243

8856 5777

8856 7361

8856 10599

8861 3815

8874 177

8874 355

8874 356

8874 595

8874 596

8874 597

8874 598

8874 1278

8874 1400

8874 1999

8874 3037

8874 3091

8874 3135

8874 3456

8874 3458

8874 3460

8874 3553

8874 3558

8874 3559

8874 3569

8874 3662

8874 4276

8874 4790

8874 4929

8874 4973

8874 5284

8874 5966

8874 6347

8874 6352

8874 6363

8874 6775

8874 7124

8874 7157

8874 7412

8874 8795

8874 64127

8874 727897

8880 672

8880 4609

8929 401

8929 7857

8929 8929

9112 54828

9314 350

9314 1026

9314 3681

9314 8884

9516 7124

9519 4763

9569 3224

9569 7135

9611 2064

9970 126

9970 211

9970 1555

9970 1557

9970 1558

9970 1559

9970 1576

9970 4843

9970 5915

9971 335

9971 344

9971 570

9971 2172

9971 5465

9971 5777

9971 8647

9971 9965

9971 10998

9971 28234

9971 116519

10062 19

10062 348

10062 1071

10062 2172

10062 6517

10062 7099

10062 7124

10062 9619

10062 10062

10189 6006

10320 925

10320 2166

10320 2264

10320 5981

10320 6573

10320 6775

10320 7433

10379 6363

10379 9636

10661 350

10664 350

10664 351

10664 672

10664 1080

10664 3481

10664 4609

10664 5292

10664 5347

10664 7015

10664 283120

10725 1571

10725 7124

11016 2064

11278 7020

11279 350

22926 3309

23114 3558

23373 3576

23373 6275

23493 7422

23528 2520

23528 89874

25942 1869

26257 174

29072 226

29072 1906

29072 2023

29072 2056

29072 5054

29072 5230

29072 5465

29072 7015

29072 7018

29072 7422

29072 8553

29072 79365

29842 1583

30818 2353

50624 4846

50943 1437

50943 3558

50943 7276

51043 1280

51176 100

51176 595

51176 1638

51176 4286

51176 6955

51222 3150

51274 720

51341 4609

51621 6352

51643 7157

55226 7015

55311 8578

55536 3456

55536 4938

55809 1583

55810 722

55893 3064

56731 3064

56938 5054

57410 632

59348 1647

64100 2353

64100 2735

64100 5777

64784 3576

80135 1956

80709 958

80709 959

84337 54106

89884 5449

126626 7066

128209 350

200186 3576

219285 5328

338917 6010

373863 1027

373863 26524

474256 350

474257 350

100271849 3725

TF-miRNA regulatory links:

EntrezID of TF miRNA name Regulatory Type

862 miR-223 Repression

3725 miR-21 Activation

3725 miR-21 Activation

650 miR-24 Activation

650 miR-31 Activation

654 miR-21 Repression

672 miR-146a Activation

672 miR-146b Activation

1050 miR-1 Activation

595 miR-17 Activation

595 miR-20a Activation

4602 miR-15a Activation

1385 miR-132 Activation

1869 miR-106a Regulation

1869 miR-106b Regulation

1869 miR-17 Activation

1869 miR-17 Regulation

1869 miR-18a Activation

1869 miR-18b Regulation

1869 miR-19a Activation

1869 miR-19b Activation

1869 miR-19b Regulation

1869 miR-20a Activation

1869 miR-20b Regulation

1869 miR-25 Regulation

1869 miR-363 Regulation

1869 miR-92a Activation

1869 miR-92a Regulation

1869 miR-93 Regulation

1958 miR-106a Regulation

27161 let-7a Activation

27161 let-7b Activation

27161 let-7c Activation

27161 let-7d Activation

27161 let-7e Activation

27161 let-7f Activation

27161 let-7g Activation

27161 let-7i Activation

2099 miR-21 Activation

50943 miR-155 Activation

50943 miR-155 Activation

6046 let-7a Repression

6046 mir-143 Repression

6046 mir-15b Repression

2623 miR-144 Activation

2623 miR-451 Activation

3091 miR-210 Activation

3159 miR-101 Activation

3159 miR-196a Activation

3159 miR-29a Activation

3159 miR-331 Activation

6927 miR-194 Activation

6927 miR-194 Regulation

3236 miR-7 Activation

3553 miR-146a Activation

3553 miR-9 Activation

4005 miR-142 Repression

3664 miR-9 Activation

4204 miR-184 Repression

50804 miR-1 Activation

50804 miR-133a Activation

4618 miR-1 Activation

4618 miR-133a Activation

4618 miR-206 Activation

4617 miR-1 Activation

4617 miR-206 Activation

4609 let-7a Repression

4609 let-7b Repression

4609 let-7c Repression

4609 let-7d Repression

4609 let-7e Repression

4609 let-7f Repression

4609 let-7g Repression

4609 let-7i Repression

4609 miR-106a Activation

4609 miR-15a Repression

4609 miR-16 Repression

4609 miR-17 Activation

4609 miR-17 Activation

4609 miR-18a Activation

4609 miR-18a Activation

4609 miR-195 Repression

4609 miR-19a Activation

4609 miR-19a Activation

4609 miR-19b Activation

4609 miR-19b Activation

4609 miR-20a Activation

4609 miR-20a Activation

4609 miR-22 Repression

4609 miR-221 Activation

4609 miR-23a Repression

4609 miR-23b Repression

4609 miR-26a Repression

4609 miR-26a Repression

4609 miR-29a Repression

4609 miR-29c Repression

4609 miR-34a Repression

4609 miR-92a Activation

4609 miR-92a Activation

4613 miR-106a Activation

4613 miR-17 Activation

4613 miR-17 Activation

4613 miR-18a Activation

4613 miR-18a Activation

4613 miR-19a Activation

4613 miR-19a Activation

4613 miR-19b Activation

4613 miR-19b Activation

4613 miR-20a Activation

4613 miR-20a Activation

4613 miR-221 Activation

4613 miR-92a Activation

4613 miR-92a Activation

4617 miR-133a Activation

4654 miR-1 Activation

4654 miR-133a Activation

4654 miR-206 Activation

4656 miR-1 Activation

4656 miR-133a Activation

4656 miR-206 Activation

4781 miR-21 Repression

4790 miR-125b Repression

4790 miR-146a Activation

4790 miR-155 Activation

4790 miR-29a Repression

4790 miR-29b Repression

4790 miR-29c Repression

4790 miR-9 Activation

1482 miR-17 Activation

1482 miR-18a Activation

1482 miR-19a Activation

1482 miR-19b Activation

1482 miR-20a Activation

1482 miR-92a Activation

2104 miR-127 Activation

2104 miR-433 Activation

5460 miR-302a Activation

5460 miR-302a Regulation

5460 miR-302b Activation

5460 miR-302b Regulation

5460 miR-302c Activation

5460 miR-302c Regulation

5460 miR-302d Activation

5460 miR-302d Regulation

5460 miR-367 Activation

5460 miR-367 Regulation

5460 miR-302a Activation

5460 miR-302b Activation

5460 miR-302c Activation

5460 miR-302d Activation

5460 miR-367 Activation

5154 miR-221 Activation

7704 miR-146a Repression

5578 miR-15a Repression

5728 miR-19a Regulation

5728 miR-21 Regulation

5978 miR-21 Repression

5978 miR-21 Repression

132625 miR-302a Activation

132625 miR-302b Activation

132625 miR-302c Activation

132625 miR-302d Activation

132625 miR-367 Activation

861 miR-27a Activation

8431 miR-127 Repression

8431 miR-433 Repression

8487 miR-141 Repression

8487 miR-200a Repression

8487 miR-200b Repression

8487 miR-200c Repression

8487 miR-429 Repression

8487 miR-8 Repression

4089 miR-155 Activation

6657 miR-302a Activation

6657 miR-302a Activation

6657 miR-302a Regulation

6657 miR-302b Activation

6657 miR-302b Activation

6657 miR-302b Regulation

6657 miR-302c Activation

6657 miR-302c Activation

6657 miR-302c Regulation

6657 miR-302d Activation

6657 miR-302d Activation

6657 miR-302d Regulation

6657 miR-367 Activation

6657 miR-367 Activation

6657 miR-367 Regulation

6667 miR-106a Regulation

7040 miR-141 Repression

7040 miR-155 Activation

7040 miR-200a Repression

7040 miR-200b Repression

7040 miR-200c Repression

7040 miR-205 Repression

7040 miR-23a Activation

7040 miR-24 Activation

7040 miR-24 Repression

7040 miR-27a Activation

7040 miR-429 Repression

2147 miR-222 Activation

7097 miR-9 Activation

7099 miR-9 Activation

51284 miR-9 Activation

51311 miR-9 Activation

7101 miR-9 Repression

3195 miR-17 Activation

3195 miR-18a Activation

3195 miR-19a Activation

3195 miR-19b Activation

3195 miR-20a Activation

3195 miR-92a Activation

30012 miR-17 Activation

30012 miR-18a Activation

30012 miR-19a Activation

30012 miR-19b Activation

30012 miR-20a Activation

30012 miR-92a Activation

7124 miR-9 Activation

7157 miR-145 Activation

7157 miR-192 Activation

7157 miR-192 Activation

7157 miR-192 Activation

7157 miR-215 Activation

7157 miR-215 Activation

7157 miR-34a Activation

7157 miR-34a Activation

7157 miR-34a Activation

7157 miR-34a Activation

7157 miR-34a Activation

7157 miR-34a Activation

7157 miR-34b Activation

7157 miR-34b Activation

7157 miR-34b Activation

7157 miR-34c Activation

7157 miR-34c Activation

7157 miR-34c Activation

22954 let-7a Activation

22954 let-7b Activation

22954 let-7c Activation

22954 let-7d Activation

22954 let-7e Activation

22954 let-7f Activation

22954 let-7g Activation

22954 let-7i Activation

7291 miR-199a Repression

7291 miR-214 Repression

7428 miR-210 Activation

7528 miR-29a Repression

7528 miR-29b Repression

7528 miR-29c Repression

6935 miR-141 Repression

6935 miR-200a Repression

6935 miR-200b Repression

6935 miR-200c Repression

6935 miR-429 Repression

6935 miR-8 Repression

9839 miR-141 Repression

9839 miR-200a Repression

9839 miR-200b Repression

9839 miR-200c Repression

9839 miR-429 Repression

9839 miR-8 Repression

1960 miR-195 Activation

1869 let-7a Activation

1869 let-7b Activation

1869 let-7c Activation

1869 let-7d Activation

1869 let-7i Activation

1869 mir-15b Activation

1869 mir-15a Activation

1869 mir-16 Activation

1869 mir-195 Activation

1869 mir-106b Activation

1869 mir-25 Activation

1869 mir-93 Activation

1871 let-7a Activation

1871 let-7b Activation

1871 let-7c Activation

1871 let-7d Activation

1871 let-7i Activation

1871 mir-15b Activation

1871 mir-15a Activation

1871 mir-16 Activation

1871 mir-195 Activation

1871 mir-106b Activation

1871 mir-25 Activation

1871 mir-93 Activation

6688 miR-23a Activation

7040 let-7d Repression

5728 miR-22 Activation

5728 miR-25 Activation

5728 miR-302 Activation

2099 miR-221 Activation

2099 miR-222 Activation

7157 miR-34a Activation

6426 miR-7 Activation

6426 miR-221 Activation

6426 miR-222 Activation

3456 miR143 Activation

3458 miR143 Activation

3456 miR145 Activation

3458 miR145 Activation

7097 miR-146a Activation

7040 miR-146a Activation

6688 miR-146a Activation

2309 miR-21 Repression

6657 miR-137 Regulation

2146 miR-137 Regulation

4204 miR-137 Regulation

10365 mir-126 Activation

3569 miR-519c Regulation

3565 miR-29a Repression

5155 miR-29a Repression

7040 miR-29a Repression

9971 miR-34a Repression

7157 miR-34a Activation

7157 miR-34c Activation

7157 miR-125b Repression

7157 miR-155 Repression

1960 miR-195 regulatory network

3091 miR-372 Activation

3091 miR-373 Activation

7291 miR-372 Activation

7291 miR-373 Activation

3845 miR-21 Activation

4893 miR-21 Activation

3265 miR-21 Activation

3569 miR-148a Repression

3569 miR-152 Repression

3569 miR-301 Repression

657 miR-21 Regulation

658 miR-21 Regulation

7040 miR-224 Activation

8742 miR-1 Repression

8742 miR-133a Repression

8742 miR-133b Repression

8742 miR-206 Repression

8742 miR-146a Activation

8742 miR-455 Activation

8742 miR-21 Regulation

4613 miR-19a Activation

4613 miR-18a Activation

4613 miR-421 Regulation

4798 miR-146a Activation

4609 miR-141 Regulation

51297 miR-141 Regulation

5728 miR-21 Regulation

1017 miR-449 auto-regulatory feedback circuit

1018 miR-449 auto-regulatory feedback circuit

1019 miR-449 auto-regulatory feedback circuit

1020 miR-449 auto-regulatory feedback circuit

1021 miR-449 auto-regulatory feedback circuit

1022 miR-449 auto-regulatory feedback circuit

1024 miR-449 auto-regulatory feedback circuit

1025 miR-449 auto-regulatory feedback circuit

5925 miR-449 auto-regulatory feedback circuit

1869 miR-449 auto-regulatory feedback circuit

1869 miR-223 autoregulatory negative feedback loop

7040 miR-181b Activation

3755 miR-146a Activation

5154 miR-24 Regulation

7040 miR-24 Regulation

6776 miR-15 Repression

6776 miR-16 Repression

1050 miR-223 Regulation

4609 miR-17 Regulation

4609 miR-19a Regulation

4609 miR-19b Regulation

4609 miR-200 regulation

4609 miR-429 regulation

4609 miR-141 regulation

7124 miR-31 Activation

7124 miR-17 Activation

207 let-7e Activation

207 miR-181c Activation

207 miR-155 Repression

207 miR-125b Repression

4088 miR-451 Activation

389421 let-7g Repression

103 mir-376a regulation

2099 mir-17 Activation

2099 mir-18a Activation

2099 mir-19a Activation

2099 mir-19b Activation

2099 mir-20a Activation

2099 mir-92a Activation

2099 mir-106a Activation

2099 mir-18b Activation

2099 mir-19b Activation

2099 mir-20b Activation

2099 mir-363 Activation

2099 mir-92a Activation

6774 miR-21 Activation

miRNA-target regulatory links:

miRNA name Target name

let-7 HMGA2

let-7 KRAS

let-7 NRAS

let-7a HMGA2

let-7a KRAS

let-7a lin-28

let-7a NF2

let-7a NRAS

let-7a raver2

let-7a TRIM71

let-7b AARSD1

let-7b AKAP8

let-7b ANAPC1

let-7b ATAD3B

let-7b ATP6V0A1

let-7b ATP6V1F

let-7b AURKB

let-7b BAT2

let-7b BCL7A

let-7b CALCOCO2

let-7b CAPG

let-7b CARHSP1

let-7b CCND1

let-7b CDC25A

let-7b CDIPT

let-7b CDK6

let-7b CDKAL1

let-7b CHMP2A

let-7b COMMD9

let-7b CSNK1D

let-7b DHX57

let-7b DOCK5

let-7b DSP

let-7b DUSP12

let-7b DUSP23

let-7b F2

let-7b FADS2

let-7b FAM105A

let-7b FAM96A

let-7b FNDC3A

let-7b GEMIN7

let-7b GPR56

let-7b GRPEL2

let-7b GTPBP3

let-7b GYS1

let-7b HMGA1

let-7b HMGA2a

let-7b IFIT5

let-7b IFRD1

let-7b IGF2BP1

let-7b IPO4

let-7b KIAA0409

let-7b Lin28

let-7b MARS2

let-7b MLLT1

let-7b MRM1

let-7b MRPS24

let-7b MRPS33

let-7b MTPN

let-7b MTRR

let-7b NEDD4

let-7b NXN

let-7b OPRS1

let-7b PGRMC1

let-7b POLD2

let-7b POLR2C

let-7b POM121

let-7b PPP1R7

let-7b PRIM1

let-7b PTGS2

let-7b PXDN

let-7b RBM19

let-7b RDH10

let-7b RHOB

let-7b RHOG

let-7b RPP38

let-7b SCYL1

let-7b SLC1A4

let-7b SLC25A1

let-7b SLC25A13

let-7b SLC25A24

let-7b SLC25A32

let-7b SNAP23

let-7b SPCS3

let-7b SPRYD4

let-7b TAF9B

let-7b THBS1

let-7b TMEM2

let-7b TRMT1

let-7b TTC9C

let-7b TYMS

let-7b UGT8

let-7b UHRF1

let-7b VPS39

let-7c c-MYC

let-7c HMGA2

let-7c HMGA2a

let-7c TRIM71

let-7e HMGA2a

let-7e SMC1L1

let-7g HMGA2

let-7g IGF2BP1

let-7g IMP-1

let-7i TLR4

miR-1 ABHD11

miR-1 ACPL2

miR-1 ADAR

miR-1 ADPGK

miR-1 AGMAT

miR-1 AGRN

miR-1 ANKIB1

miR-1 ANKRD29

miR-1 ANP32B

miR-1 ANPEP

miR-1 ANXA2

miR-1 AP3B1

miR-1 AP3D1

miR-1 ARCN1

miR-1 ARF3

miR-1 ARF4

miR-1 ARHGEF18

miR-1 ARID1A

miR-1 ARID2

miR-1 ASH2L

miR-1 ATP6V0A1

miR-1 AXL

miR-1 BCKDHB

miR-1 BDNF

miR-1 BLCAP

miR-1 BRI3BP

miR-1 BRWD2

miR-1 C20orf139

miR-1 C20orf9

miR-1 C2orf3

miR-1 CAND1

miR-1 CAP1

miR-1 CDCP1

miR-1 CDW92

miR-1 CHST11

miR-1 CHSY1

miR-1 CLCN3

miR-1 CLG

miR-1 COIL

miR-1 CORO1C

miR-1 CPOX

miR-1 CSRP1

miR-1 CT120

miR-1 CTEN

miR-1 CTSC

miR-1 CX43

miR-1 DDX5

miR-1 DHX15

miR-1 DNAJB1

miR-1 EGFR

miR-1 EHMT1

miR-1 EHMT2

miR-1 EML4

miR-1 EPB41L4B

miR-1 F2

miR-1 FBLN2

miR-1 FLJ20273

miR-1 FLJ20519

miR-1 FLJ21415

miR-1 G6PD

miR-1 GAK

miR-1 GCH1

miR-1 GJA1

miR-1 GNPDA2

miR-1 GNPNAT1

miR-1 GOLGA7

miR-1 GPD2

miR-1 H3F3B

miR-1 Hand2

miR-1 HCN2

miR-1 HCN4

miR-1 HDAC4

miR-1 HIST1H3B

miR-1 HIST1H3I

miR-1 HPS4

miR-1 HSP60

miR-1 HSP70

miR-1 IHPK2

miR-1 INPP5F

miR-1 IQGAP3

miR-1 ITGB4

miR-1 KCNE1

miR-1 KCNJ2

miR-1 KCNQ1

miR-1 KIAA1160

miR-1 KIAA1194

miR-1 KIAA1295

miR-1 KIAA1340

miR-1 KIAA1598

miR-1 KIAA1618

miR-1 KIF2

miR-1 KIS

miR-1 LASP1

miR-1 LASS2

miR-1 LIN7C

miR-1 LOC126731

miR-1 LOC63929

miR-1 LRP1

miR-1 LRRC8

miR-1 LRRC8A

miR-1 LZTFL1

miR-1 MET

miR-1 MGC26690

miR-1 MGC27345

miR-1 MMD

miR-1 MOV10

miR-1 MRC2

miR-1 MTHFD2

miR-1 MTX1

miR-1 MXD4

miR-1 NETO2

miR-1 NOTCH2

miR-1 NP

miR-1 NRP1

miR-1 OAT

miR-1 OSBPL7

miR-1 PARG1

miR-1 PDCD4

miR-1 PDLIM7

miR-1 PFTK1

miR-1 PGM2

miR-1 PICALM

miR-1 PIP3AP

miR-1 PLEKHB2

miR-1 PLEKHC1

miR-1 POGK

miR-1 POLA2

miR-1 POLR2K

miR-1 POM121

miR-1 PPIB

miR-1 PREX1

miR-1 PRSS21

miR-1 PTBP1

miR-1 PTBP2

miR-1 PTK9

miR-1 PTMA

miR-1 PTMAP7

miR-1 PTPLAD1

miR-1 PTPLB

miR-1 PTPRF

miR-1 PWP1

miR-1 RAB11FIP2

miR-1 RABGAP1L

miR-1 RABL2A

miR-1 RABL2B

miR-1 RFT1

miR-1 RNF138

miR-1 SAC3D1

miR-1 SDC4

miR-1 SEC23IP

miR-1 SERP1

miR-1 SERPINB5

miR-1 SFRS9

miR-1 SFXN1

miR-1 SH2D4A

miR-1 SH3BGRL3

miR-1 SLC16A9

miR-1 SLC25A1

miR-1 SLC25A22

miR-1 SLC25A30

miR-1 SNX6

miR-1 SSNA1

miR-1 SYNE1

miR-1 TAGLN2

miR-1 TDP1

miR-1 TH1L

miR-1 THBS1

miR-1 TIMP3

miR-1 TIP120A

miR-1 TM4SF7

miR-1 TMSB4X

miR-1 TPM1

miR-1 TPM2

miR-1 TPM3

miR-1 TPM4

miR-1 TRAPPC3

miR-1 TRIM2

miR-1 UHRF1

miR-1 UNC93B1

miR-1 UST

miR-1 WDFY1

miR-1 XPO6

miR-1 YWHAQ

miR-1 ZNF264

miR-101 Enx-1

miR-101 EZH2

miR-101 ICOS

miR-101 MYCN

miR-101 N-MYC

miR-103 FBXW1B

miR-103 ICOS

miR-103 serbp1

miR-103-1 FBXW1B

miR-106a AML1

miR-106a ARID4B

miR-106a CDKN1A

miR-106a HIPK3

miR-106a MYLIP

miR-106a p21

miR-106a RB1

miR-106a VEGF

miR-106b CDKN1A

miR-106b E2F1

miR-106b p21

miR-106b VEGF

miR-107 BACE1

miR-107 NF1-A

miR-107 NFIA

miR-107 serbp1

miR-10a HOXA1

miR-10b HOXD10

miR-122 Aldolase A

miR-122 Cat1

miR-122 CAT-1

miR-122 cyclin G1

miR-122 GTF2b

miR-122 GYS1

miR-122 TRPV6

miR-122a CCNG1

miR-124 ABHD5

miR-124 ACAA2

miR-124 ACTR8

miR-124 AHR

miR-124 AHRR

miR-124 AK2

miR-124 ALDH9A1

miR-124 ANKRD15

miR-124 ANKRD27

miR-124 ANXA8

miR-124 AP1M2

miR-124 APEX2

miR-124 ARAF1

miR-124 ARFIP1

miR-124 ARH

miR-124 ARHGEF1

miR-124 ARPC1B

miR-124 ARRDC1

miR-124 ATP6V0E

miR-124 B4GALT1

miR-124 BTG3

miR-124 C10orf56

miR-124 C14orf24

miR-124 C14orf32

miR-124 C18orf37

miR-124 C1QDC1

miR-124 C2orf12

miR-124 C2ORF25

miR-124 C3orf4

miR-124 C6orf72

miR-124 C9orf88

miR-124 CAV1

miR-124 CD164

miR-124 CD59

miR-124 CDC14B

miR-124 CDCA7

miR-124 CDK4

miR-124 CDK6

miR-124 CEBPA

miR-124 CHODL

miR-124 CHP

miR-124 CHSY1

miR-124 Contig3845_RC

miR-124 CPNE3

miR-124 CREB3L2

miR-124 CT120

miR-124 CTDSP1

miR-124 CTDSP2

miR-124 CTGF

miR-124 CTNND1

miR-124 CYP1B1

miR-124 D4ST1

miR-124 DCTD

miR-124 DEPDC1

miR-124 DFFB

miR-124 DHCR24

miR-124 DNAJC1

miR-124 DNM2

miR-124 DVL2

miR-124 E2F5

miR-124 E2IG4

miR-124 ELF4

miR-124 ELOVL1

miR-124 ELOVL5

miR-124 EPIM

miR-124 ERH

miR-124 EYA4

miR-124 F11R

miR-124 FA2H

miR-124 FAM104A

miR-124 FAM35A

miR-124 FCHO2

miR-124 FLJ10099

miR-124 FLJ10420

miR-124 FLJ11259

miR-124 FLJ20273

miR-124 FLJ20364

miR-124 FLJ20847

miR-124 FLJ21924

miR-124 FLJ25084

miR-124 FLJ46072

miR-124 FN5

miR-124 G3BP

miR-124 GAS2L1

miR-124 GCL

miR-124 GNAI3

miR-124 GNG10

miR-124 GSN

miR-124 HADHB

miR-124 HADHSC

miR-124 HEBP2

miR-124 HIC

miR-124 HSPC135

miR-124 HTATIP2

miR-124 IFRD2

miR-124 IQGAP1

miR-124 ITGB1

miR-124 JAKMIP1

miR-124 KATNA1

miR-124 KIAA0830

miR-124 KIAA1102

miR-124 KIS

miR-124 LAMC1

miR-124 LASS2

miR-124 LITAF

miR-124 LMNB1

miR-124 LOC116064

miR-124 LOC285908

miR-124 LOC339924

miR-124 LOC55974

miR-124 LRRC1

miR-124 MAD2L2

miR-124 MAN2A1

miR-124 MAPK14

miR-124 MGC17943

miR-124 MGC20741

miR-124 MGC4083

miR-124 MGC5508

miR-124 MGC62100

miR-124 MPHOSPH9

miR-124 MTMR6

miR-124 MTPN

miR-124 MYH9

miR-124 MYO10

miR-124 NEK6

miR-124 NEK9

miR-124 NFATC1

miR-124 NFIC

miR-124 NID1

miR-124 NM_014445

miR-124 NM_018211

miR-124 NM_032121

miR-124 NME4

miR-124 NS5ATP13TP2

miR-124 OSBPL8

miR-124 PAPSS2

miR-124 PARG1

miR-124 PARP16

miR-124 PDLIM7

miR-124 PECI

miR-124 PGF

miR-124 PGM1

miR-124 PGRMC2

miR-124 PHF19

miR-124 PLDN

miR-124 PLOD3

miR-124 PLP2

miR-124 PLSCR3

miR-124 PODXL

miR-124 POLR3G

miR-124 PP1201

miR-124 PRKD1

miR-124 PTBP1

miR-124 PTPN12

miR-124 PTTG1IP

miR-124 RAI

miR-124 RAM2

miR-124 RARG

miR-124 RASSF5

miR-124 RBMS1

miR-124 RDH10

miR-124 RELA

miR-124 RFFL

miR-124 RHOG

miR-124 RNPEPL1

miR-124 RYK

miR-124 SCP1

miR-124 SENP8

miR-124 SERP1

miR-124 SERPINB6

miR-124 SLC15A4

miR-124 SLC16A1

miR-124 SLC17A5

miR-124 SLC22A5

miR-124 SLC25A30

miR-124 SLC30A7

miR-124 SMAD5

miR-124 SNAI2

miR-124 SP1

miR-124 SPC18

miR-124 SSFA2

miR-124 STOM

miR-124 STX10

miR-124 SUCLG2

miR-124 SURF4

miR-124 SWAP70

miR-124 SYCP1

miR-124 SYNGR2

miR-124 SYPL

miR-124 TARBP1

miR-124 TBDN100

miR-124 TEAD1

miR-124 THG-1

miR-124 TJP2

miR-124 TLN1

miR-124 TNFRSF21

miR-124 TOM1L1

miR-124 TRIM29

miR-124 TRIP11

miR-124 TSPAN15

miR-124 TTC7A

miR-124 TWIST2

miR-124 UHRF1

miR-124 USP48

miR-124 VAMP3

miR-124 ZBED3

miR-124 ZFP36L2

miR-124a CTDSP1

miR-124a VAMP3

miR-125a ERBB2

miR-125a ERBB3

miR-125a Lin28

miR-125a lin-28

miR-125b ADAMTS1

miR-125b AK123478

miR-125b B3GALT4

miR-125b BAK1

miR-125b C10orf104

miR-125b CASP6

miR-125b CASP7

miR-125b CBLN2

miR-125b CBX7

miR-125b CEBPG

miR-125b CLU

miR-125b CYP1A1

miR-125b DIO3

miR-125b ERBB2

miR-125b ERBB3

miR-125b FAM19A1

miR-125b GPR160

miR-125b H3F3B

miR-125b HIST1H4A

miR-125b HOMER2

miR-125b ID1

miR-125b ID2

miR-125b ID3

miR-125b IGFBP3

miR-125b IL1RN

miR-125b JMJ

miR-125b Lin28

miR-125b lin-28

miR-125b MAN1A1

miR-125b MGC138396

miR-125b ODZ2

miR-125b PCDHB10

miR-125b PERP

miR-125b PIGR

miR-125b RBM8A

miR-125b SGPL1

miR-125b trkC

miR-125b TSPAN8

miR-125b UBE2I

miR-125b UGT2B15

miR-125b UGT2B17

miR-125b UGT2B28

miR-126 prostein

miR-126 SLC45A3

miR-126 VCAM1

miR-126 VCAM-1

miR-127 BCL6

miR-127 Peg11

miR-127 Rtl1

miR-129 CAMTA1

miR-129 EIF2C3

miR-129 Notch1

miR-130 CSF1

miR-130 MAFB

miR-130 MCSF

miR-130a GAX

miR-130a HOXA5

miR-130a MAFB

miR-130a MCSF

miR-130a MEOX2

miR-130a Tac1

miR-132 PGC

miR-132 RFX4

miR-132 RICS

miR-133 ERG

miR-133 HCN2

miR-133 KCNE1

miR-133 PTBP2

miR-133 SRF

miR-133a Casp9

miR-133a HCN2

miR-133a HERG

miR-133a KCNQ1

miR-133a PKM2

miR-133b nPTB

miR-133b Pitx3

miR-133b PKM2

miR-136 Peg11

miR-136 Rtl1

miR-137 CDK6

miR-137 E2F6

miR-137 NCOA2/TIF2

miR-138 TERT

miR-140 HDAC4

miR-140 VEGF

miR-141 CLOCK

miR-141 hmgb1

miR-141 serbp1

miR-141 sfpq

miR-141 TGF beta2

miR-143 ERK5

miR-143 MAPK12

miR-143 MAPK7

miR-145 FLJ21308

miR-145 IRS1

miR-145 IRS-1

miR-147 VEGF

miR-148 DNMT3B

miR-148a Dnmt3b1

miR-148a Dnmt3b3

miR-148a PXR

miR-148b Dnmt3b1

miR-15 BCL2

miR-15 DMTF1

miR-150 MYB

miR-155 AGTR1

miR-155 AMIGO2

miR-155 ANKFY1

miR-155 ARFIP1

miR-155 ARFIP2

miR-155 ARID2

miR-155 ARL10

miR-155 ARL5B

miR-155 AT1R

miR-155 ATG3

miR-155 ATP6V1C1

miR-155 BACH1

miR-155 BACH-1

miR-155 BET1

miR-155 BRPF3

miR-155 CBFB

miR-155 CDK5RAP3

miR-155 CEBPB

miR-155 CHAF1A

miR-155 CLDN1

miR-155 CTNNB1

miR-155 CUL4B

miR-155 CYP51A1

miR-155 DHX40

miR-155 DNAJB1

miR-155 DNAJC19

miR-155 DPP7

miR-155 DSG2

miR-155 F2

miR-155 FADS1

miR-155 FADS3

miR-155 FMNL2

miR-155 GNA13

miR-155 HSD17B12

miR-155 HSDL1

miR-155 KIAA0776

miR-155 LDOC1

miR-155 LPL

miR-155 LY6K

miR-155 LYCAT

miR-155 MATR3

miR-155 MET

miR-155 METTL7A

miR-155 MLSTD2

miR-155 MOSC1

miR-155 MOSPD2

miR-155 MPZL1

miR-155 MSI2

miR-155 MYO10

miR-155 MYO1E

miR-155 NARS

miR-155 NT5E

miR-155 PDE3A

miR-155 PDLIM5

miR-155 PHC2

miR-155 PICALM

miR-155 PKN2

miR-155 PLXND1

miR-155 PODXL

miR-155 POLE3

miR-155 POLE4

miR-155 PPL

miR-155 PPP5C

miR-155 PRAF2

miR-155 PRKCI

miR-155 PTPRJ

miR-155 RAB23

miR-155 RAB27B

miR-155 RAB34

miR-155 RAB5C

miR-155 RAB6A

miR-155 RAI14

miR-155 RCN2

miR-155 RCOR1

miR-155 RHEB

miR-155 SCAMP1

miR-155 SDCBP

miR-155 SH3BP4

miR-155 SLC30A1

miR-155 SMAD1

miR-155 SNAP29

miR-155 SYNE2

miR-155 SYPL1

miR-155 TACSTD2

miR-155 TBCA

miR-155 TM6SF1

miR-155 TNFRSF10A

miR-155 TP53INP1

miR-155 TRAM1

miR-155 TRIM32

miR-155 TRIP13

miR-155 TXNDC12

miR-155 TXNRD1

miR-155 UBE2J1

miR-155 VAMP3

miR-155 WDFY1

miR-155 WDR68

miR-15a Actr1A

miR-15a ASXL2

miR-15a BCL2

miR-15a C10orf104

miR-15a C14orf109

miR-15a CARD8

miR-15a CDC14B

miR-15a CENPJ

miR-15a Cep63

miR-15a CREBL2

miR-15a DMTF1

miR-15a ECHDC1

miR-15a FLJ10287

miR-15a FLJ13955

miR-15a FLJ20534

miR-15a FLJ21820

miR-15a FLJ33167

miR-15a GOLGA5

miR-15a GOLPH3L

miR-15a GTF2H1

miR-15a H3F3B

miR-15a HACE1

miR-15a HDHD2

miR-15a HERC6

miR-15a HLC-8

miR-15a HRSP12

miR-15a HSDL2

miR-15a HSPA1A

miR-15a IGSF4

miR-15a JUN

miR-15a KIAA1935

miR-15a LOC159091

miR-15a LOC339804

miR-15a LOC388650

miR-15a MCL1

miR-15a MSH2

miR-15a NT5C2L1

miR-15a OMA1

miR-15a OSGEPL1

miR-15a PDCD4

miR-15a PDCD6IP

miR-15a PHKB

miR-15a PMS1

miR-15a PNN

miR-15a PRIM1

miR-15a RAB21

miR-15a Rab9B

miR-15a RAD51C

miR-15a RHOT1

miR-15a RNASEL

miR-15a SCAP2

miR-15a SLC35A1

miR-15a SLC35B3

miR-15a TIA1

miR-15a Tpi1

miR-15a TRA1

miR-15a UGDH

miR-15a UGP2

miR-15a VEGF

miR-15a VPS45A

miR-15a WASPIP

miR-15a Wt1

miR-15a ZNF559

miR-15b BCL2

miR-15b Bcl-2

miR-16 ABCF2

miR-16 ABHD10

miR-16 ACP2

miR-16 Actr1A

miR-16 ADSS

miR-16 ARHGDIA

miR-16 ARL2

miR-16 ASXL2

miR-16 ATG9A

miR-16 BCL2

miR-16 Bcl-2

miR-16 C10orf104

miR-16 C14orf109

miR-16 CA12

miR-16 CACNA2D1

miR-16 CARD8

miR-16 CCND1

miR-16 CDC14B

miR-16 CDK5RAP1

miR-16 CENPJ

miR-16 Cep63

miR-16 Cfl2

miR-16 CGI-38

miR-16 CHORDC1

miR-16 CREBL2

miR-16 Crhbp

miR-16 Cshl1

miR-16 DNAJB4

miR-16 ECHDC1

miR-16 EGFR

miR-16 EIF4E

miR-16 F2

miR-16 FGF2

miR-16 FLJ10287

miR-16 FLJ13955

miR-16 FLJ20534

miR-16 FLJ21820

miR-16 FLJ33167

miR-16 FNDC3B

miR-16 GALNT7

miR-16 GFM1

miR-16 GFPT1

miR-16 GNL3L

miR-16 GOLGA5

miR-16 GOLPH3L

miR-16 GPAM

miR-16 GSTM4

miR-16 GTF2H1

miR-16 H3F3B

miR-16 HACE1

miR-16 HARS

miR-16 HARS2

miR-16 HBXIP

miR-16 HDHD2

miR-16 HERC6

miR-16 HLC-8

miR-16 HMOX1

miR-16 HRSP12

miR-16 HSDL2

miR-16 HSPA1A

miR-16 HSPA1B

miR-16 IFRD1

miR-16 IFRD2

miR-16 IGF2R

miR-16 IGSF4

miR-16 IPO4

miR-16 ITGA2

miR-16 JUN

miR-16 KCNN4

miR-16 KIAA1935

miR-16 KPNA3

miR-16 LAMC1

miR-16 LOC159091

miR-16 LOC339804

miR-16 LOC388650

miR-16 LUZP1

miR-16 LYPLA2

miR-16 MAP2K1IP1

miR-16 MCL1

miR-16 MLLT1

miR-16 MLLT11

miR-16 MRPL20

miR-16 MSH2

miR-16 NAPG

miR-16 NARG1

miR-16 NAT6

miR-16 NOTCH2

miR-16 NP

miR-16 NPR3

miR-16 NT5C2L1

miR-16 OMA1

miR-16 OSGEPL1

miR-16 PAFAH1B2

miR-16 PANX1

miR-16 PDCD4

miR-16 PDCD6IP

miR-16 PHKB

miR-16 PHLDB2

miR-16 PISD

miR-16 PLK1

miR-16 PMS1

miR-16 PNN

miR-16 PPIF

miR-16 PPP2R5C

miR-16 PRIM1

miR-16 PSAT1

miR-16 PTGS2

miR-16 PURA

miR-16 RAB21

miR-16 RAB30

miR-16 Rab9B

miR-16 RAD51C

miR-16 RARS

miR-16 RFT1

miR-16 RHOT1

miR-16 RNASEL

miR-16 RTN4

miR-16 SCAP2

miR-16 SEC24A

miR-16 SERPINE2

miR-16 SHOC2

miR-16 SLC12A2

miR-16 SLC16A3

miR-16 SLC25A22

miR-16 SLC35A1

miR-16 SLC35B3

miR-16 SLC38A1

miR-16 SLC38A5

miR-16 SLC7A1

miR-16 SNX15

miR-16 SPTLC1

miR-16 SQSTM1

miR-16 SRPR

miR-16 SRPRB

miR-16 TIA1

miR-16 TMEM109

miR-16 TMEM43

miR-16 TNFSF9

miR-16 TOMM34

miR-16 Tpi1

miR-16 TPM3

miR-16 TRA1

miR-16 TXN2

miR-16 UBE2S

miR-16 UBE2V1

miR-16 UBE4A

miR-16 UGDH

miR-16 UGP2

miR-16 UTP15

miR-16 VEGF

miR-16 VPS45A

miR-16 VTI1B

miR-16 WASPIP

miR-16 Wt1

miR-16 YIF1B

miR-16 ZNF559

miR-16 ZNF622

miR-17 AIB1

miR-17 AML1

miR-17 CDKN1A

miR-17 E2F1

miR-17 NCOA3

miR-17 p21

miR-17 VEGF

miR-181 TCL1A

miR-181a HOXA11

miR-181b GRIA2

miR-181b Tcl1

miR-181b VSNL1

miR-182 Adcy6

miR-182 Mitf

miR-189 SLITRK1

miR-192 SIP1

miR-192 ZEB2

miR-193a E2F6

miR-193a MCL1

miR-193a PTK2/FAK

miR-196 HOXA7

miR-196 HOXB8

miR-196 HOXC8

miR-196 HOXD8

miR-196a HOXA7

miR-196a HOXB8

miR-196a HOXC8

miR-196a HOXD8

miR-199a IKKB

miR-199a MET

miR-199b LAMC2

miR-19a PTEN

miR-19b HIPK3

miR-19b MYLIP

miR-1b BDNF

miR-1b G6PD

miR-20 E2F1

miR-200 ZEB1

miR-200 ZEB2

miR-200a SIP1

miR-200a TCF8

miR-200a ZEB1

miR-200a ZEB2

miR-200b RERE

miR-200b SIP1

miR-200b TCF8

miR-200b ZEB1

miR-200c SIP1

miR-200c TCF8

miR-200c ZEB1

miR-200c ZEB2

miR-203 p63

miR-203 SOCS-3

miR-205 SIP1

miR-205 VEGF

miR-205 ZEB1

miR-206 CX43

miR-206 ER alpha

miR-206 ESR1

miR-206 FSTL1

miR-206 GJA1

miR-206 Tac1

miR-206 UTRN

miR-20a AML1

miR-20a E2F1

miR-20a E2F-1

miR-20a TGFBR2

miR-20a VEGF

miR-20b ARID4B

miR-20b CDKN1A

miR-20b HIPK3

miR-20b MYLIP

miR-20b p21

miR-20b VEGF

miR-21 ACTA2

miR-21 APAF1

miR-21 BMPRII

miR-21 BPPRII

miR-21 BTG2

miR-21 CDK6

miR-21 CDKN1A

miR-21 CFL2

miR-21 FAM3C

miR-21 FAS

miR-21 Glcci1

miR-21 HIPK3

miR-21 IL-6R

miR-21 maspin

miR-21 NFIB

miR-21 PDCD4

miR-21 PRRG4

miR-21 PTEN

miR-21 RP2

miR-21 SERPINB5

miR-21 SESN1

miR-21 SGK3

miR-21 SLC16A10

miR-21 SOCS5

miR-21 TPM1

miR-210 EFNA3

miR-210 Ephrin-A3

miR-212 ZO-1

miR-214 PTEN

miR-218 LAMB3

miR-219 SCOP

miR-22 ESR1

miR-22 PPARA

miR-221 CDKN1B

miR-221 CDKN1C

miR-221 KIP1

miR-221 KIT

miR-221 p27

miR-221 p27Kip1

miR-221 p57

miR-222 CDKN1B

miR-222 CDKN1C

miR-222 KIP1

miR-222 KIT

miR-222 p27

miR-222 p27Kip1

miR-222 p57

miR-223 NFIA

miR-223 NFI-A

miR-224 API5

miR-224 API-5

miR-23 HES1

miR-23 POU4F2

miR-23a Brn-3b

miR-23a CXCL12

miR-23a FLJ13158

miR-23a Hes1

miR-23a SDF-1

miR-23b NOTCH1

miR-24 ALK4

miR-24 CDKN2A

miR-24 DHFR

miR-24 INK4a

miR-24 MAPK14

miR-24 NOTCH1

miR-24 p16

miR-24 SLITRK1

miR-26 SMAD1

miR-26a Ezh2

miR-26a PLAG1

miR-26a serbp1

miR-26a SMAD1

miR-26a TGFBR2

miR-26b serbp1

miR-27a MYT1

miR-27a Sp1

miR-27a Sp3

miR-27a Sp4

miR-27a ZBTB10

miR-27b CYP1B1

miR-27b Notch1

miR-29 TCL1A

miR-29a BACE1

miR-29a DNMT3A

miR-29a DNMT3B

miR-29b BACE1

miR-29b dnajb11

miR-29b DNMT3A

miR-29b DNMT3B

miR-29b Mcl1

miR-29b sfpq

miR-29b Tcl1

miR-29c COL15A1

miR-29c COL1A1

miR-29c COL1A2

miR-29c COL3A1

miR-29c COL4A1

miR-29c COL4A2

miR-29c DNMT3A

miR-29c DNMT3B

miR-29c fibrillin 1

miR-29c FUSIP1

miR-29c LAMC1

miR-29c SPARC

miR-29c TDG

miR-30 AADACL1

miR-30 ADPGK

miR-30 ANPEP

miR-30 AP2A1

miR-30 ATP2A2

miR-30 ATRX

miR-30 CBFB

miR-30 CDCP1

miR-30 CEP72

miR-30 CHD1

miR-30 CPNE8

miR-30 DOCK7

miR-30 ELMOD2

miR-30 F2

miR-30 FRG1

miR-30 FXR2

miR-30 GALNT1

miR-30 GALNT7

miR-30 GNAI2

miR-30 GPD2

miR-30 HNRPM

miR-30 IDH1

miR-30 IFRD1

miR-30 ITGA2

miR-30 JUN

miR-30 KDELC2

miR-30 KRTHB5

miR-30 LMNB2

miR-30 LRRC8C

miR-30 MAT2A

miR-30 MBNL1

miR-30 MET

miR-30 MLLT1

miR-30 MLLT11

miR-30 MPDU1

miR-30 MYO10

miR-30 NAPG

miR-30 NCL

miR-30 NP

miR-30 NPR3

miR-30 NT5C3

miR-30 NT5E

miR-30 NUCB1

miR-30 NUFIP2

miR-30 P4HA2

miR-30 PAFAH1B2

miR-30 PEX11B

miR-30 PGM1

miR-30 PPP2R4

miR-30 PPP3CA

miR-30 PPP3R1

miR-30 PRPF40A

miR-30 PTGFRN

miR-30 PTPRK

miR-30 PTRH1

miR-30 RAB27B

miR-30 RAD23B

miR-30 RBMS1

miR-30 RQCD1

miR-30 SEC23A

miR-30 SLC12A4

miR-30 SLC38A1

miR-30 SLC38A2

miR-30 SLC4A10

miR-30 SLC4A7

miR-30 SLC7A1

miR-30 SLC7A11

miR-30 SLC9A3R2

miR-30 STRN

miR-30 STX7

miR-30 SYPL1

miR-30 THEM4

miR-30 TICAM2

miR-30 TLOC1

miR-30 TMCO1

miR-30 TMED10

miR-30 TMED2

miR-30 TMED3

miR-30 TMED7

miR-30 TMEM41B

miR-30 TMEM59

miR-30 TMEM87A

miR-30 TNFAIP2

miR-30 TNFRSF10B

miR-30 UAP1

miR-30 WNT5A

miR-30 ZNF294

miR-302d VEGF

miR-30a CDK6

miR-30a cyr61

miR-30a Notch1

miR-30a pro2730

miR-30a ro2730

miR-30a SLC7A6

miR-30a THBS1

miR-30a TMEM113

miR-30a TMEM2

miR-30a tuba3

miR-30a vezatin

miR-30a VEZT

miR-32 PFV-1

miR-330 VEGF

miR-335 MERTK

miR-335 PTPRN2

miR-335 SOX4

miR-34 DLL1

miR-34 NOTCH1

miR-34a BCL2

miR-34a CCND1

miR-34a CDK6

miR-34a Delta1

miR-34a E2F3

miR-34a MYCN

miR-34a Notch1

miR-34a VEGF

miR-34b VEGF

miR-370 MAP3K8

miR-372 CDKN1A

miR-372 LATS2

miR-372 p21

miR-372 VEGF

miR-373 ADAM9

miR-373 AOF1

miR-373 ARHGEF3

miR-373 BAZ1A

miR-373 C11orf15

miR-373 C20orf142

miR-373 C2orf18

miR-373 C5orf5

miR-373 C9orf78

miR-373 CD24

miR-373 CD44

miR-373 CD83

miR-373 CDK11

miR-373 CENPF

miR-373 CFL2

miR-373 CKLFSF4

miR-373 EG1

miR-373 FLJ25555

miR-373 FLJ34236

miR-373 Fus1

miR-373 FYCO1

miR-373 GBAS

miR-373 GBP3

miR-373 GLTP

miR-373 GPSM2

miR-373 HERPUD1

miR-373 HSPA14

miR-373 INSIG2

miR-373 KIAA1194

miR-373 KIAA1399

miR-373 KIAA1919

miR-373 KIF23

miR-373 KLHL12

miR-373 LATS2

miR-373 LMNB1

miR-373 LOC149420

miR-373 LOC51035

miR-373 LOC93081

miR-373 LUC7L2

miR-373 MGC17943

miR-373 MGC29898

miR-373 MICA

miR-373 MKRN1

miR-373 MYBL1

miR-373 NCB5OR

miR-373 NEDD5

miR-373 NIN

miR-373 NUPL1

miR-373 PHC2

miR-373 PRC1

miR-373 RELA

miR-373 RNF149

miR-373 RNF159

miR-373 RPIA

miR-373 SLC25A23

miR-373 STK4

miR-373 STX11

miR-373 TBC1D2

miR-373 TFAP4

miR-373 TMEM14A

miR-373 TNFAIP1

miR-373 TOPK

miR-373 TTC8

miR-373 TUSC2

miR-373 USP12

miR-373 VEGF

miR-373 VPS26

miR-373 ZHX1

miR-373 ZNF226

miR-375 ADIPOR2

miR-375 C1QBP

miR-375 JAK2

miR-375 MTPN

miR-375 USP1

miR-376a PRPS1

miR-376a SFRS11

miR-376a SLC16A1

miR-376a SNX19

miR-376a TTK

miR-378 Fus1

miR-378 Fus-1

miR-378 SuFu

miR-378 TUSC2

miR-378 VEGF

miR-424 Galectin-3

miR-424 NFI-A

miR-429 RERE

miR-431 Peg11

miR-431 Rtl1

miR-433 FGF20

miR-433 Peg11

miR-433 Rtl1

miR-434 Peg11

miR-434 Rtl1

miR-504 VEGF

miR-520c CD44

miR-520g VEGF

miR-520h ABCG2

miR-520h SMAD6

miR-520h VEGF

miR-7 EGFR

miR-7 IRS-1

miR-7 IRS-2

miR-9 BACE1

miR-9 trkC

miR-92-2 HIPK3

miR-92-2 MYLIP

miR-93 E2F1

miR-93 p21

miR-93 VEGF

miR-96 Adcy6

miR-96 HTR1B

miR-96 Mitf

miR-98 HMGA2

miR-99a raver2

miR-99b raver2
