# Supplementary material for: microRNA evolution in a human transcription factor and microRNA regulatory network
Source: BMC Syst Biol. 2010 Jun 29;4:90. doi: 10.1186/1752-0509-4-90 (PMC2914650; doi:10.1186/1752-0509-4-90)
Supplement: Additional file 3 — Evolutionary rates of protein-coding genes. This file contains the evolutionary rates of protein-coding genes. [file 1752-0509-4-90-S3.DOC]

**Additional file 3.** This file contains the evolutionary rates of protein-coding genes.

EntrezGene dN

1 0.431

9 0.159

14 0.007

19 0.027

20 0.039

22 0.044

23 0.042

25 0.075

26 0.134

31 0.014

33 0.072

34 0.066

35 0.057

36 0.099

37 0.093

40 0.004

47 0.009

48 0.038

50 0.018

51 0.07

52 0.089

53 0.063

55 0.105

58 0.001

70 0.003

72 0.001

81 0.009

86 0.007

87 0.004

88 0.005

90 0.009

91 0.01

97 0.055

98 0.121

100 0.112

102 0.02

103 0.124

105 0.1

107 0.051

108 0.026

109 0.029

111 0.016

115 0.049

117 0.041

118 0.02

119 0.031

120 0.042

123 0.107

125 0.364

127 0.179

128 0.047

132 0.046

134 0.033

140 0.176

141 0.103

142 0.044

150 0.044

156 0.01

157 0.09

159 0.017

160 0.007

161 0.014

162 0.025

163 0.001

164 0.004

165 0.087

172 0.085

174 0.223

176 0.133

183 0.292

185 0.032

186 0.044

191 0.018

196 0.164

197 0.276

199 0.062

202 0.086

203 0.06

204 0.029

205 0.054

207 0.01

208 0.012

210 0.068

211 0.043

213 0.175

215 0.049

216 0.077

217 0.026

218 0.118

219 0.031

220 0.037

224 0.087

225 0.032

226 0.011

229 0.023

231 0.079

241 0.045

242 0.08

244 0.043

246 0.174

249 0.086

257 0.047

258 0.218

259 0.158

262 0.01

267 0.027

268 0.184

269 0.118

271 0.014

273 0.079

274 0.021

275 0.072

284 0.015

286 0.055

287 0.01

288 0.031

291 0.022

292 0.007

302 0.013

307 0.053

309 0.033

311 0.409

312 0.191

313 0.137

314 0.12

317 0.074

318 0.066

320 0.009

321 1.097

322 0.028

323 0.034

327 0.045

328 0.029

330 0.16

331 0.056

333 0.057

334 0.039

343 0.149

345 0.277

351 0.013

355 0.401

359 0.067

361 0.041

363 0.157

364 0.164

366 0.164

368 0.126

369 0.032

372 0.006

373 0.014

374 0.175

375 0

377 0

382 0

383 0.078

384 0.088

387 0.003

388 0

390 0.002

395 0.049

397 0.063

400 0.008

403 0.013

405 0.042

406 0.008

407 0.109

408 0.008

409 0.017

411 0.131

419 0.311

420 0.263

427 0.105

430 0.186

432 0.114

433 0.218

439 0.001

440 0.033

443 0.072

462 0.072

468 0.081

472 0.092

473 0.03

474 0.057

476 0.018

477 0.006

478 0.002

479 0.286

480 0.104

481 0.036

483 0.187

487 0.113

488 0.005

489 0.024

490 0.002

492 0.014

493 0.077

495 0.016

496 0.097

498 0.015

509 0.042

513 0.083

516 0.059

518 0.021

522 0.098

523 0.011

525 0.035

526 0.006

528 0.012

535 0.022

539 0.105

540 0.098

549 0.037

551 0.126

558 0.067

563 0.281

570 0.217

571 0.117

572 0.128

573 0.162

575 0.031

576 0.027

577 0.008

578 0.121

579 0.087

581 0.057

583 0.058

585 0.055

586 0.086

587 0.098

590 0.132

593 0.041

594 0.043

595 0.033

596 0.066

597 0.171

598 0.013

602 0.096

604 0.027

610 0.028

623 0.159

627 0.014

629 0.096

631 0.21

634 0.335

636 0.277

637 0.265

639 0.062

642 0.035

644 0.114

648 0.012

649 0.02

652 0.013

653 0.035

655 0.012

656 0.075

657 0.011

658 0.009

659 0.018

660 0.049

661 0.033

662 0.044

663 0.045

664 0.028

665 0.015

666 0.022

667 0.201

669 0.045

673 0.01

676 0.169

677 0.012

678 0.041

685 0.125

686 0.096

688 0.042

695 0.009

699 0.155

701 0.136

705 0.052

706 0.125

712 0.202

715 0.149

716 0.162

717 0.158

719 0.24

720 0.17

722 0.354

726 0.045

729 0.154

730 0.178

734 0.041

735 0.254

740 0.102

744 0.144

745 0.057

747 0.015

752 0.063

753 0.048

754 0.146

757 0.009

758 0.009

762 0.344

767 0.007

768 0.173

770 0.018

771 0.109

775 0.244

778 0.05

780 0.04

782 0.013

783 0.027

784 0.01

785 0.011

788 0.039

790 0.025

793 0.007

794 0.296

797 0.173

800 0.753

801 0

808 0

813 0.01

815 0.001

816 0.005

817 0

818 0

820 0.334

821 0.037

824 0.038

825 0.028

826 0.03

827 0.026

828 0.254

830 0.008

833 0.054

835 0.057

836 0.078

839 0.062

840 0.244

841 0.226

842 0.142

844 0.028

845 0.067

847 0.057

857 0.027

858 0.05

862 0.003

863 0.052

864 0.215

865 0.021

866 0.299

867 0.036

875 0.092

881 0.038

885 0.112

894 0.044

896 0.03

898 0.13

899 0.096

900 0.039

901 0.059

902 0.026

904 0.052

905 0.071

908 0.016

912 0.244

914 0.39

915 0.254

916 0.288

919 0.122

920 0.357

921 0.257

922 0.222

924 0.358

925 0.415

926 0.38

931 0.146

932 0.302

933 0.309

940 0.174

941 0.458

942 0.4

945 0.376

946 0.383

949 0.123

950 0.077

953 0.148

954 0.098

955 0.081

956 0.118

957 0.064

959 0.127

962 0.383

963 0.108

969 0.301

970 0.357

971 0.367

974 0.238

976 0.28

977 0.044

978 0.108

983 0.017

984 0.022

987 0.054

988 0.011

989 0.005

990 0.121

991 0.033

993 0.102

994 0.106

995 0.186

997 0.003

998 0

1000 0.019

1001 0.1

1003 0.157

1004 0.018

1005 0.011

1006 0.019

1007 0.047

1009 0.018

1013 0.091

1014 0.143

1015 0.14

1016 0.03

1017 0.005

1018 0.089

1019 0.028

1020 0.011

1021 0.02

1022 0.027

1026 0.141

1027 0.064

1028 0.26

1032 0.076

1033 0.201

1039 0.063

1045 0.032

1056 0.136

1058 0.18

1059 0.042

1066 0.141

1068 0.058

1069 0.022

1070 0.006

1071 0.556

1072 0.009

1073 0.003

1075 0.137

1102 0.026

1103 0.076

1104 0.033

1105 0.022

1106 0.031

1108 0.007

1109 0.19

1111 0.036

1113 0.187

1116 0.17

1119 0.07

1122 0.124

1123 0.019

1124 0.009

1133 0.056

1134 0.032

1138 0.245

1140 0.058

1146 0.054

1147 0.024

1149 0.101

1152 0.021

1153 0.029

1154 0.051

1158 0.165

1161 0.054

1163 0

1173 0

1179 0.157

1181 0.034

1184 0.012

1186 0.019

1192 0.01

1197 0.052

1198 0.01

1200 0.065

1201 0.086

1203 0.207

1208 0.164

1211 0.018

1212 0.324

1213 0.001

1215 0.202

1232 0.196

1233 0.085

1234 0.425

1236 0.074

1238 0.187

1260 0.033

1261 0.101

1262 0.047

1263 0.043

1264 0.011

1266 0.012

1267 0.082

1272 0.024

1278 0.062

1281 0.054

1284 0.118

1287 0.049

1289 0.22

1290 0.037

1291 0.058

1292 0.056

1293 0.098

1295 0.035

1296 0.029

1297 0.061

1299 0.051

1302 0.029

1303 0.043

1305 0.053

1306 0.178

1311 0.059

1314 0.009

1315 0.008

1316 0.025

1317 0.045

1318 0.149

1327 0.129

1329 0.101

1337 0.1

1339 0.144

1340 0.087

1349 0.144

1352 0.112

1353 0.101

1355 0.059

1358 0.088

1359 0.107

1361 0.1

1362 0.044

1364 0.088

1366 0.047

1368 0.089

1369 0.099

1370 0.222

1371 0.127

1373 0.025

1374 0.075

1382 0.037

1385 0.001

1386 0.015

1388 0.068

1389 0.028

1390 0.231

1392 0.104

1395 0.074

1397 0.038

1398 0.002

1399 0.018

1400 0.016

1406 0.439

1408 0.023

1410 0.011

1411 0.023

1412 0.047

1414 0.122

1417 0.045

1419 0.088

1427 0.059

1428 0.065

1431 0.027

1434 0

1435 0.19

1440 0.184

1441 0.263

1448 0.488

1452 0

1453 0.001

1455 0.027

1456 0.006

1457 0.006

1463 0.209

1468 0.063

1474 0.194

1475 0.258

1482 0.054

1488 0.003

1489 0.136

1491 0.092

1493 0.169

1495 0.004

1497 0.098

1503 0.016

1509 0.122

1510 0.108

1511 0.229

1512 0.1

1513 0.078

1515 0.16

1519 0.179

1522 0.093

1523 0.02

1524 0.109

1525 0.063

1527 0.285

1528 0.071

1534 0.072

1536 0.045

1537 0.052

1540 0.027

1543 0.119

1545 0.115

1553 0.466

1556 0.587

1558 0.172

1573 0.169

1579 0.16

1580 0.093

1582 0.163

1584 0.261

1586 0.238

1588 0.126

1593 0.171

1595 0.051

1600 0.017

1602 0.004

1603 0

1606 0.105

1607 0.018

1610 0.114

1611 0.029

1613 0.102

1615 0.021

1616 0.197

1618 0.057

1620 0.007

1622 0.137

1627 0.063

1635 0.037

1636 0.098

1641 0.005

1642 0.003

1643 0.119

1645 0.179

1646 0.164

1654 0.009

1656 0.011

1657 0.054

1662 0.094

1663 0.16

1665 0.007

1674 0.012

1675 0.235

1676 0.145

1677 0.206

1678 0.023

1687 0.193

1690 0.03

1716 0.148

1717 0.064

1719 0.051

1723 0.073

1728 0.07

1729 0.049

1730 0.085

1731 0.032

1733 0.143

1736 0.044

1737 0.079

1738 0.027

1743 0.04

1745 0.008

1747 0.01

1749 0.016

1750 0.003

1755 0.322

1756 0.046

1759 0.003

1761 0.092

1767 0.06

1768 0.009

1769 0.04

1770 0.07

1773 0.151

1774 0.199

1776 0.117

1777 0.182

1778 0.006

1780 0.01

1781 0.011

1783 0.016

1785 0.011

1786 0.15

1787 0.11

1788 0.02

1789 0.086

1791 0.123

1793 0.019

1795 0.01

1796 0.097

1798 0.035

1800 0.189

1803 0.084

1804 0.08

1806 0.059

1808 0.007

1809 0.008

1810 0.003

1811 0.119

1812 0.047

1813 0.024

1816 0.09

1819 0.007

1822 0.039

1824 0.146

1825 0.124

1826 0.008

1827 0.029

1828 0.129

1831 0.041

1832 0.032

1833 0.107

1834 0.449

1836 0.114

1837 0.003

1838 0.177

1839 0.116

1840 0.015

1841 0.11

1842 0.14

1843 0.017

1845 0.035

1846 0.079

1847 0.036

1848 0.014

1849 0.006

1855 0.031

1856 0.018

1857 0.015

1859 0.015

1861 0.237

1869 0.087

1870 0.096

1871 0.021

1876 0.095

1879 0

1889 0.031

1892 0.071

1894 0.049

1896 0.034

1901 0.036

1902 0.02

1908 0.279

1909 0.046

1911 0.043

1912 0.063

1933 0.028

1936 0.084

1937 0.011

1939 0.102

1944 0.017

1945 0.098

1947 0.023

1949 0.026

1951 0.042

1952 0.023

1953 0.229

1954 0.02

1955 0.065

1956 0.078

1959 0.054

1964 0

1965 0.004

1968 0.002

1969 0.041

1974 0

1975 0.03

1978 0.041

1979 0.024

1981 0.043

1982 0.007

1983 0.012

1994 0.007

1995 0.005

1996 0.001

1998 0.042

1999 0.067

2000 0.121

2001 0.027

2004 0.04

2005 0.088

2009 0.048

2010 0.147

2013 0.145

2015 0.218

2016 0.016

2017 0.043

2018 0.002

2022 0.199

2026 0.01

2027 0.014

2029 0.008

2030 0.172

2034 0.068

2035 0.036

2036 0.026

2037 0.128

2039 0.023

2042 0.019

2043 0.008

2046 0.066

2047 0.006

2048 0.002

2049 0.016

2050 0.049

2051 0.041

2052 0.095

2053 0.169

2054 0.09

2055 0.094

2057 0.109

2059 0.059

2060 0.058

2063 0.024

2065 0.051

2070 0.032

2073 0.209

2077 0.018

2078 0.016

2081 0.04

2091 0.017

2098 0.049

2099 0.062

2100 0.088

2101 0.008

2103 0.037

2104 0.001

2107 0

2109 0.042

2110 0.039

2113 0.014

2114 0.035

2115 0.024

2116 0.201

2119 0.019

2120 0.047

2121 0.201

2123 0.432

2131 0.007

2132 0.026

2135 0.069

2137 0.014

2138 0.021

2139 0.075

2140 0.028

2145 0.012

2146 0.01

2147 0.118

2149 0.14

2151 0.181

2152 0.341

2159 0.2

2161 0.185

2162 0.076

2167 0.045

2168 0.093

2169 0.133

2170 0.083

2171 0.107

2172 0.118

2173 0.079

2175 0.231

2176 0.202

2177 0.152

2178 0.238

2180 0.083

2181 0.038

2182 0.017

2185 0.026

2186 0.058

2187 0.364

2188 0.419

2189 0.167

2192 0.085

2193 0.049

2194 0.141

2196 0.109

2197 0.011

2201 0.021

2202 0.03

2209 0.19

2213 0.277

2218 0.055

2222 0.063

2230 0.155

2232 0.078

2235 0.064

2239 0.027

2242 0.059

2245 0.025

2246 0.022

2250 0.082

2252 0.027

2253 0.008

2255 0.426

2256 0.018

2257 0.049

2258 0.023

2259 0.062

2260 0.009

2262 0.083

2263 0.018

2264 0.064

2268 0.085

2271 0.042

2273 0.027

2277 0.079

2286 0.021

2287 0.028

2288 0.059

2289 0.079

2298 0.264

2299 0.071

2301 0.088

2302 0.039

2305 0.117

2307 0.144

2308 0.034

2309 0.025

2314 0.026

2317 0.024

2318 0.01

2319 0.005

2323 0.223

2327 0.08

2328 0.136

2329 0.12

2330 0.096

2331 0.041

2335 0.043

2348 0.129

2350 0.141

2353 0.033

2355 0.024

2356 0.111

2358 0.176

2359 0.274

2395 0.205

2444 0.061

2475 0.006

2483 0.013

2491 0.172

2494 0.055

2512 0.098

2515 0.295

2516 0.036

2517 0.103

2519 0.108

2524 0.292

2530 0.018

2531 0.039

2533 0.131

2534 0.042

2535 0.002

2539 0.052

2542 0.041

2548 0.128

2549 0.053

2550 0.008

2551 0.019

2553 0.009

2554 0.006

2556 0.024

2557 0.055

2558 0.022

2560 0.01

2562 0.015

2563 0.038

2565 0.026

2566 0.007

2567 0.023

2568 0.039

2571 0.028

2572 0.024

2580 0.102

2581 0.097

2583 0.076

2584 0.068

2585 0.068

2588 0.094

2589 0.01

2590 0.02

2591 0.044

2615 0.133

2617 0.028

2618 0.083

2620 0.014

2621 0.111

2622 0.053

2623 0.081

2625 0.017

2626 0.037

2628 0.026

2631 0.043

2637 0.008

2639 0.073

2645 0.04

2648 0.011

2649 0.007

2650 0.092

2651 0.137

2657 0.117

2658 0.09

2660 0.021

2662 0.094

2665 0.018

2668 0.041

2669 0.025

2670 0.044

2671 0.111

2672 0.085

2673 0.006

2674 0.036

2675 0.032

2676 0.14

2681 0.188

2683 0.076

2686 0.02

2693 0.022

2705 0.016

2710 0.015

2717 0.134

2719 0.031

2720 0.152

2729 0.03

2730 0.021

2733 0.127

2735 0.079

2736 0.078

2742 0.009

2743 0.016

2744 0.024

2747 0.036

2752 0.038

2760 0.21

2762 0.02

2764 0.007

2767 0.022

2768 0.074

2770 0

2771 0.012

2773 0.008

2774 0.038

2775 0.01

2776 0.003

2778 0.003

2779 0.003

2780 0.024

2782 0.001

2784 0.018

2794 0.025

2799 0.045

2801 0.134

2802 0.109

2803 0.184

2810 0.03

2813 0.251

2815 0.205

2820 0.041

2821 0.067

2823 0.005

2824 0.008

2825 0.113

2826 0.07

2827 0.032

2828 0.641

2829 0.194

2830 0.03

2834 0.061

2835 0.037

2838 0.155

2841 0.088

2842 0.061

2845 0.016

2847 0.049

2849 0.026

2850 0.012

2852 0.076

2857 0.054

2861 0.134

2862 0.244

2865 0.17

2870 0.019

2872 0.032

2873 0.013

2877 0.029

2879 0.024

2880 0.144

2882 0.06

2885 0.002

2887 0.101

2888 0.076

2889 0.045

2891 0.008

2894 0.008

2895 0.043

2896 0.16

2897 0.012

2898 0.008

2901 0.012

2907 0.038

2909 0.423

2913 0.019

2918 0.011

2919 0.304

2922 0.199

2926 0.043

2932 0.008

2934 0.05

2935 0.007

2936 0.081

2937 0.068

2938 0.168

2939 0.345

2944 0.097

2948 0.059

2949 0.105

2958 0.004

2959 0.002

2962 0.037

2963 0.013

2965 0.016

2966 0.017

2967 0.033

2968 0.007

2969 0.016

2970 1.85

2972 0.062

2974 0.078

2978 0.047

2979 0.075

2982 0.055

2983 0.008

2987 0.071

2997 0.026

2999 0.313

3000 0.086

3001 0.195

3002 0.261

3003 0.159

3004 0.231

3009 0.15

3021 0.003

3024 0.119

3026 0.14

3029 0.07

3030 0.078

3032 0.047

3033 0.053

3038 0.033

3045 0.164

3046 0.1

3054 0.002

3059 0.075

3060 0.114

3061 0.037

3062 0.028

3064 0.053

3065 0.004

3067 0.071

3068 0.064

3069 0.012

3070 0.047

3073 0.101

3074 0.163

3080 0.254

3081 0.037

3084 0.064

3087 0.037

3091 0.049

3092 0.064

3093 0

3094 0.032

3098 0.044

3099 0.035

3104 0.051

3108 0.151

3111 0.172

3117 0.188

3127 0.252

3131 0.009

3135 0.228

3142 0.071

3156 0.041

3157 0.249

3158 0.057

3159 0.02

3161 0.157

3164 0.046

3169 0.024

3170 0.012

3171 0.398

3174 0.025

3178 0.001

3181 0.003

3182 0.03

3183 0.011

3184 0.012

3185 0.009

3189 0.005

3190 0

3191 0.01

3192 0.028

3196 0.038

3198 0.037

3200 0.031

3202 0.011

3203 0.022

3204 0.139

3207 0.011

3208 0

3209 0.01

3214 0.625

3216 0.621

3221 0.004

3224 0.002

3226 0.014

3227 0.011

3229 0.006

3231 0.094

3233 0.026

3235 0.062

3236 0.007

3240 0.128

3242 0.062

3248 0.057

3249 0.062

3257 0.114

3265 0

3267 0.017

3274 0.121

3275 0.071

3280 0.008

3284 0.205

3290 0.141

3292 0.223

3295 0.074

3297 0.052

3305 0.034

3306 0.004

3308 0.023

3309 0.007

3312 0.008

3313 0.011

3321 0.04

3326 0.003

3329 0.014

3336 0.024

3337 0.023

3338 0.181

3344 0.028

3350 0.065

3361 0.072

3363 0.061

3364 0.09

3376 0.052

3384 0.281

3386 0.227

3396 0.127

3397 0.027

3400 0.009

3421 0.026

3423 0.081

3425 0.129

3429 0.291

3430 0.187

3431 0.418

3443 0.77

3448 0.271

3454 0.368

3455 0.407

3458 0.483

3459 0.366

3460 0.319

3475 0.031

3476 0.104

3483 0.145

3485 0.069

3488 0.027

3489 0.179

3490 0.06

3500 0.287

3512 0.149

3547 0.065

3549 0.025

3550 0.012

3551 0.042

3552 0.254

3553 0.196

3556 0.171

3560 0.299

3568 0.223

3572 0.142

3574 0.187

3575 0.246

3577 0.217

3579 0.205

3581 0.241

3586 0.166

3587 0.345

3588 0.202

3589 0.074

3590 0.098

3594 0.326

3597 0.15

3600 0.168

3601 0.492

3603 0.117

3604 0.305

3606 0.237

3607 0.06

3609 0.038

3611 0.003

3612 0.072

3613 0.045

3614 0.014

3615 0.007

3619 0.148

3621 0.078

3622 0.016

3623 0.117

3624 0.018

3625 0.018

3627 0.208

3630 0.106

3638 0.103

3640 0.325

3652 0.086

3654 0.112

3656 0.191

3659 0.08

3660 0.04

3663 0.069

3667 0.055

3670 0.001

3672 0.042

3676 0.086

3678 0.051

3679 0.084

3680 0.06

3684 0.169

3687 0.199

3688 0.62

3692 0.012

3694 0.053

3695 0.082

3696 0.069

3697 0.108

3705 0.013

3706 0.034

3707 0.133

3712 0.056

3713 0.566

3714 0.056

3717 0.033

3720 0.037

3725 0.013

3728 0.01

3743 0.045

3747 0.015

3748 0.042

3749 0.015

3750 0.028

3751 0.006

3752 0.004

3753 0.142

3754 0.02

3755 0.061

3756 0.036

3758 0.037

3759 0.008

3760 0.005

3761 0.018

3762 0.035

3766 0.006

3767 0.021

3768 0.016

3772 0.02

3773 0.055

3775 0.029

3776 0.046

3777 0.042

3778 0.004

3778 0.236

3779 0.097

3781 0.005

3782 0.024

3783 0.08

3784 0.056

3785 0.005

3786 0.022

3787 0.065

3788 0.01

3790 0.027

3792 0.167

3795 0.143

3797 0.259

3800 0.01

3801 0.028

3811 0.476

3814 0.29

3816 0.24

3821 0.426

3824 0.309

3831 0.036

3833 0.126

3835 0.109

3837 0.005

3839 0.004

3840 0.005

3841 0.116

3842 0.002

3843 0.014

3845 0.015

3848 0.153

3851 0.106

3853 0.088

3854 0.19

3855 0.098

3858 0.119

3860 0.109

3861 0.038

3866 0.054

3868 0.092

3872 0.03

3880 0.103

3883 0.073

3884 0.088

3885 0.09

3887 0.114

3888 0.077

3890 0.093

3892 0.074

3895 0.094

3898 0.191

3902 0.196

3903 0.514

3904 0.651

3906 0.219

3908 0.073

3909 0.136

3910 0.066

3911 0.129

3912 0.041

3913 0.464

3915 0.041

3920 0.228

3925 0.008

3927 0.022

3930 0.125

3932 0.022

3934 0.275

3936 0.017

3937 0.086

3939 0.086

3945 0.01

3948 0.155

3949 0.138

3953 0.146

3954 0.097

3955 0.037

3956 0.061

3957 0.246

3959 0.212

3960 0.152

3964 0.115

3965 0.211

3980 0.066

3981 0.08

3982 0.044

3984 0.031

3985 0.038

3987 0.024

3988 0.158

3993 0.056

3995 0.056

3998 0.06

4000 0.015

4001 0.017

4005 0.006

4007 0.06

4008 0.121

4009 0.014

4010 0.001

4013 0.256

4015 0.071

4023 0.043

4025 0.129

4026 0.06

4034 0.083

4037 0.04

4038 0.019

4040 0.011

4041 0.034

4043 0.16

4045 0.006

4047 0.09

4048 0.038

4051 0.12

4054 0.044

4055 0.218

4060 0.078

4062 0.057

4063 0.327

4065 0.144

4067 0.036

4068 0.062

4069 0.304

4071 0.128

4072 0.088

4074 0.041

4084 0.067

4085 0.031

4086 0.004

4087 0.003

4089 0.008

4090 0.005

4091 0.032

4092 0.01

4093 0.015

4094 0.018

4097 0.006

4099 0.036

4100 0.54

4102 0.55

4107 0.479

4108 0.467

4108 0.472

4109 0.33

4116 0

4117 0.095

4118 0.078

4121 0.051

4123 0.057

4124 0.147

4125 0.17

4126 0.157

4128 0.073

4129 0.056

4133 0.182

4134 0.232

4135 0.244

4139 0.031

4140 0.018

4141 0.064

4143 0.022

4144 0.002

4145 0.062

4146 0.061

4147 0.075

4148 0.089

4149 0

4151 0.096

4152 0.195

4154 0.001

4155 0.053

4159 0.078

4160 0.04

4161 0.105

4162 0.151

4166 0.11

4168 0.158

4170 0.123

4171 0.028

4172 0.105

4173 0.028

4175 0.025

4176 0.043

4179 0.439

4184 0.517

4185 0.027

4189 0.031

4190 0.019

4194 0.095

4200 0.064

4201 0.04

4204 0.024

4207 0.205

4209 0.026

4210 0.367

4211 0.001

4212 0.004

4213 0.194

4214 0.06

4215 0.017

4216 0.06

4221 0.017

4225 0.145

4232 0.012

4233 0.058

4234 0.058

4237 0.049

4238 0.094

4240 0.276

4241 0.096

4242 0.096

4245 0.061

4247 0.062

4248 0.037

4253 0.144

4254 0.097

4255 0.212

4256 0.083

4258 0.121

4281 0.024

4282 0.058

4284 0.037

4286 0.031

4287 0.083

4289 0.005

4291 0.103

4292 0.069

4293 0.007

4296 0.032

4297 0.051

4301 0.044

4308 0.183

4311 0.031

4312 0.32

4313 0.023

4316 0.292

4319 0.144

4320 0.141

4324 0.07

4325 0.011

4326 0.072

4327 0.121

4331 0.029

4332 0.544

4336 0.062

4338 0.088

4340 0.077

4342 0.165

4343 0.048

4345 0.132

4351 0.087

4354 0.039

4355 0.012

4356 0.048

4359 0.032

4361 0.066

4363 0.065

4430 0.012

4436 0.042

4438 0.063

4439 0.062

4440 0.004

4478 0.007

4482 0.096

4507 0.037

4521 0.145

4524 0.05

4528 0.106

4534 0.042

4542 0.037

4544 0.11

4552 0.138

4582 0.458

4585 0.854

4586 0.287

4587 0.166

4588 0.244

4591 0.045

4593 0.035

4595 0.136

4597 0.091

4599 0.16

4601 0.023

4602 0.064

4604 0.044

4605 0.09

4608 0.082

4610 0.094

4613 0.086

4616 0.065

4620 0.018

4627 0.016

4629 0.022

4632 0.03

4634 0.019

4635 0.05

4638 0.051

4640 0.083

4642 0.015

4645 0.052

4646 0.027

4648 0.131

4649 0.058

4651 0.032

4653 0.112

4656 0.034

4659 0.031

4660 0.067

4661 0.049

4665 0.025

4666 0.511

4669 0.102

4674 0.097

4675 0.149

4676 0.025

4677 0.039

4678 0.104

4681 0.034

4683 0.196

4686 0.01

4687 0.103

4690 0.005

4691 0.103

4693 0.024

4695 0.071

4698 0.097

4700 0.068

4703 0.036

4706 0.074

4709 0.122

4710 0.181

4711 0.157

4712 0.222

4714 0.104

4715 0.081

4716 0.112

4718 0.192

4719 0.03

4720 0.044

4723 0.021

4724 0.071

4725 0.191

4726 0.083

4728 0.061

4733 0.003

4738 0.006

4739 0.063

4744 0.094

4745 0.032

4750 0.094

4751 0.071

4752 0.154

4753 0.033

4758 0.092

4759 0.217

4760 0.009

4762 0.059

4763 0.009

4771 0.01

4774 0.014

4775 0.057

4776 0.04

4778 0.065

4779 0.016

4780 0.102

4781 0.005

4782 0.028

4784 0.007

4790 0.077

4791 0.047

4792 0.042

4793 0.095

4796 0.15

4798 0.033

4799 0.064

4800 0.001

4801 0.005

4804 0.041

4808 0.011

4810 0.056

4814 0.06

4815 0.357

4820 0.129

4821 0.062

4824 0.156

4826 0.006

4836 0.013

4837 0.087

4838 0.121

4841 0.007

4848 1.603

4850 0.005

4851 0.376

4852 0.042

4853 0.042

4855 0.109

4856 0.116

4858 0.141

4861 0.384

4862 0.068

4864 0.086

4867 0.127

4868 0.101

4869 0.018

4878 0.104

4879 0.708

4880 0.049

4881 0.058

4882 0.006

4884 0.017

4885 0.018

4886 0.032

4892 0.079

4897 0.045

4898 0.033

4899 0.003

4901 0.065

4905 0.016

4908 0.019

4909 0.045

4914 0.068

4916 0.018

4922 0.105

4927 0.05

4928 0.033

4929 0.004

4931 0.075

4935 0.131

4938 0.23

4940 0.207

4942 0.053

4943 0.068

4948 0.133

4950 0.053

4951 0.06

4958 0.109

4967 0.04

4968 0.134

4973 0.263

4974 0.062

4976 0.022

4978 0.008

4982 0.089

4983 0.044

4987 0.033

4990 0.012

4993 0.109

4998 0.218

4999 0.126

5000 0.057

5001 0.033

5002 0.153

5005 0.456

5007 0.016

5008 0.403

5009 0.04

5010 0.033

5015 0.004

5017 0.024

5019 0.043

5020 0.071

5023 0.06

5027 0.12

5029 0.053

5033 0.027

5034 0.032

5045 0.034

5046 0.044

5048 0.001

5050 0.02

5051 0.128

5052 0.03

5053 0.046

5054 0.121

5055 0.142

5058 0.005

5063 0.011

5064 0.112

5066 0.05

5067 0.045

5069 0.047

5071 0.063

5073 0.061

5074 0.106

5075 0.058

5077 0.008

5078 0.134

5082 0.042

5083 0.007

5087 0

5091 0.018

5092 0.005

5094 0

5095 0.061

5097 0.021

5099 0.013

5100 0.031

5101 0.034

5104 0.254

5105 0.052

5108 0.063

5110 0.02

5118 0.089

5122 0.039

5125 0.032

5126 0.016

5127 0.014

5128 0.007

5129 0.049

5134 0.101

5136 0.039

5137 0.024

5138 0.034

5140 0.097

5143 0.12

5144 0.015

5147 0.013

5148 0.049

5152 0.043

5153 0.022

5156 0.046

5159 0.082

5161 0.157

5163 0.053

5164 0.013

5166 0.039

5168 0.032

5169 0.12

5170 0.027

5172 0.068

5174 0.131

5176 0.076

5179 0.104

5184 0.068

5187 0.043

5188 0.08

5189 0.058

5190 0.075

5191 0.038

5192 0.116

5193 0.067

5194 0.048

5195 0.035

5198 0.072

5199 0.139

5201 0.012

5202 0.022

5203 0.015

5204 0.003

5205 0.031

5208 0.039

5209 0.016

5210 0.015

5211 0.054

5212 0.087

5213 0.011

5214 0.07

5218 0.009

5222 0.372

5222 0.431

5224 0.045

5225 0.182

5226 0.26

5230 0.011

5232 0.076

5236 0.013

5238 0.081

5239 0.011

5241 0.116

5250 0.06

5252 0.035

5253 0.04

5257 0.038

5260 0.036

5261 0.036

5264 0.157

5265 0.252

5268 0.06

5269 0.171

5270 0.084

5272 0.195

5274 0.072

5275 0.149

5276 0.135

5277 0.071

5281 0.095

5283 0.058

5288 0.168

5289 0.013

5294 0.032

5295 0.022

5296 0.063

5297 0.014

5298 0.008

5300 0.023

5303 0.014

5304 0.449

5305 0.011

5307 0.021

5308 0.008

5309 0.008

5313 0.05

5314 0.173

5315 0.015

5316 0.019

5317 0.026

5319 0.144

5320 0.26

5325 0.201

5327 0.124

5328 0.226

5329 0.281

5331 0.045

5332 0.01

5334 0.031

5335 0.015

5336 0.036

5337 0.053

5338 0.058

5339 0.034

5342 0.19

5346 0.099

5347 0.027

5348 0.111

5349 0.178

5354 0

5358 0.005

5359 0.148

5361 0.023

5362 0.02

5364 0.076

5365 0.09

5367 0.068

5368 0.097

5371 0.202

5372 0.024

5373 0.041

5375 0.066

5376 0.08

5378 0.146

5393 0.045

5394 0.079

5395 0.138

5396 0.002

5407 0.098

5408 0.152

5409 0.094

5412 0.004

5413 0.005

5414 0.039

5420 0.467

5422 0.069

5424 0.062

5427 0.058

5429 0.11

5431 0.001

5432 0.003

5433 0.007

5434 0.008

5438 0

5439 0

5443 0.132

5446 0.115

5447 0.045

5450 0.062

5451 0.034

5452 0.009

5454 0.001

5460 0.076

5464 0.03

5465 0.039

5468 0.01

5469 0.017

5470 0.083

5471 0.045

5475 0.189

5478 0.018

5479 0.031

5481 0.035

5493 0.072

5495 0.031

5500 0

5501 0.002

5504 0.085

5506 0.108

5507 0.073

5509 0.087

5511 0.011

5514 0.027

5515 0.002

5516 0

5518 0.001

5519 0.038

5520 0

5521 0.052

5522 0.007

5525 0.012

5526 0.007

5527 0.006

5530 0.002

5532 0.004

5534 0

5537 0.002

5538 0.085

5546 0.034

5547 0.144

5549 0.053

5550 0.023

5551 0.224

5557 0.055

5558 0.062

5562 0.006

5563 0.015

5564 0.022

5565 0.012

5566 0.011

5567 0.016

5573 0.017

5576 0.076

5577 0.015

5578 0.002

5580 0.057

5581 0.013

5582 0.2

5583 0.013

5584 0.007

5585 0.047

5588 0.031

5589 0.07

5590 0.023

5595 0.019

5598 0.038

5600 0.022

5601 0.021

5602 0.007

5605 0.034

5606 0.014

5608 0.01

5609 0.009

5611 0.02

5612 0.028

5617 0.277

5618 0.172

5623 0.243

5624 0.197

5626 0.162

5634 0.006

5636 0.005

5639 0.121

5641 0.101

5648 0.262

5650 0.267

5652 0.151

5653 0.228

5655 0.517

5662 0.038

5663 0.039

5664 0.03

5678 0.551

5681 0.013

5683 0.002

5691 0.007

5692 0.039

5693 0.027

5695 0.022

5700 0.001

5701 0.002

5702 0.006

5704 0.003

5708 0.009

5711 0.054

5715 0.086

5716 0.031

5717 0.001

5718 0.014

5719 0.019

5720 0.03

5721 0.033

5723 0.031

5725 0.018

5727 0.018

5728 0.001

5730 0.152

5731 0.083

5733 0.132

5738 0.057

5739 0.651

5742 0.061

5743 0.079

5745 0.054

5746 0.124

5747 0.014

5753 0.112

5754 0.046

5756 0.025

5757 0.113

5763 0.038

5768 0.148

5770 0.109

5774 0.048

5775 0.03

5777 0.029

5778 0.052

5780 0.014

5781 0.003

5782 0.1

5783 0.113

5784 0.047

5786 0.024

5787 0.083

5788 0.223

5791 0.04

5792 0.024

5793 0.03

5796 0.008

5797 0.014

5798 0.171

5799 0.217

5800 0.055

5801 0.063

5802 0.036

5805 0.098

5810 0.056

5814 0.009

5818 0.097

5824 0.056

5825 0.025

5826 0.058

5827 0.149

5831 0.051

5832 0.026

5833 0.046

5834 0.028

5836 0.028

5837 0.018

5859 0.059

5863 0.06

5864 0.004

5865 0.014

5866 0.084

5867 0.031

5868 0.009

5869 0

5870 0.019

5871 0.035

5873 0.022

5874 0.029

5875 0.05

5876 0.022

5877 0.044

5879 0.009

5881 0

5883 0.105

5884 0.111

5886 0.029

5887 0.049

5888 0.007

5889 0.07

5890 0.076

5891 0.1

5892 0.104

5894 0.013

5896 0.054

5897 0.063

5898 0.002

5899 0.023

5900 0.073

5902 0.022

5903 0.074

5905 0.061

5906 0

5908 0

5910 0.025

5911 0.01

5912 0

5913 0.019

5914 0.01

5916 0.006

5917 0.046

5918 0.17

5921 0.023

5922 0.058

5923 0.088

5924 0.026

5925 0.047

5929 0.025

5930 0.073

5931 0.001

5932 0.147

5933 0.049

5934 0.049

5937 0.016

5940 0.257

5948 0.062

5949 0.087

5950 0.091

5954 0.035

5957 0.059

5962 0.012

5965 0.094

5968 0.185

5973 0.083

5976 0.007

5977 0.007

5979 0.106

5980 0.076

5981 0.1

5985 0.021

5987 0.01

5988 0.426

5989 0.037

5990 0.066

5991 0.005

5992 0.017

5994 0.118

5996 0.069

5997 0.031

5998 0.131

5999 0.024

6000 0.009

6001 0.041

6002 0.083

6003 0.094

6004 0.078

6006 0.325

6007 0.754

6009 0.006

6010 0.03

6014 0.041

6015 0.019

6017 0.051

6018 0.057

6039 0.243

6045 0.001

6046 0.022

6047 0.044

6049 0.155

6051 0.068

6059 0.01

6091 0.021

6092 0.023

6095 0.004

6096 0.011

6097 0.059

6098 0.126

6101 0.265

6102 0.081

6103 0.256

6117 0.075

6118 0.059

6119 0.14

6120 0.028

6122 0.011

6123 0.032

6128 0.058

6129 0.032

6133 0.007

6134 0.002

6135 0

6137 0.013

6139 0.005

6142 0.003

6143 0

6144 0.003

6144 0.032

6144 0.034

6146 0.011

6147 0.013

6152 0

6154 0

6155 0.003

6157 0.014

6158 0.007

6159 0.11

6160 0

6160 0.094

6164 0.004

6166 0.004

6168 0

6173 0

6175 0.012

6181 0.167

6182 0.08

6183 0.097

6187 0.009

6189 0.005

6191 0

6192 0.048

6193 0.005

6195 0.007

6196 0.023

6197 0.001

6198 0.003

6199 0.035

6201 0.002

6202 0

6204 0.006

6206 0.018

6207 0

6217 0

6218 0.083

6223 0.005

6224 0

6228 0

6230 0.008

6232 0

6236 0.044

6237 0.029

6238 0.112

6239 0.087

6240 0.017

6241 0.064

6242 0.082

6247 0.019

6249 0.072

6251 0.016

6252 0.107

6256 0.207

6257 0.015

6258 0.009

6263 0.06

6272 0.046

6273 0.562

6277 0.017

6279 0.314

6282 0.094

6284 0.094

6285 0.01

6289 0.235

6293 0.012

6294 0.086

6295 0.077

6302 0.067

6303 0.019

6304 0.008

6305 0.031

6311 0.035

6314 0.068

6318 0.289

6319 0.106

6320 0.092

6323 0.02

6324 0.024

6327 0.045

6328 0.013

6330 0.128

6331 0.031

6334 0.008

6336 0.113

6337 0.097

6338 0.097

6340 0.081

6342 0.063

6344 0.186

6345 0.016

6347 0.35

6351 0.144

6352 0.106

6356 0.286

6358 0.56

6359 0.56

6361 0.259

6364 0.295

6369 0.309

6372 0.33

6373 0.196

6376 0.238

6382 0.147

6383 0.095

6385 0.11

6386 0.059

6387 0.032

6388 0.031

6389 0.031

6390 0.049

6391 0.13

6392 0.097

6396 0.017

6398 0.483

6399 0.012

6400 0.04

6402 0.17

6403 0.171

6404 0.498

6405 0.019

6414 0.182

6416 0.008

6421 0.023

6422 0.027

6423 0.009

6424 0.033

6425 0.032

6426 0

6428 0

6430 0.013

6432 0.009

6434 0.002

6435 0.225

6443 0.031

6444 0.029

6446 0.018

6448 0.064

6449 0.063

6451 0.025

6452 0.031

6453 0.026

6455 0.031

6456 0.005

6468 0.078

6474 0.009

6477 0.002

6478 0.007

6480 0.116

6483 0.031

6487 0.014

6490 0.123

6494 0.057

6495 0.007

6496 0.017

6498 0.066

6499 0.037

6500 0.003

6502 0.085

6503 0.09

6504 0.266

6505 0.06

6506 0.024

6507 0.018

6509 0.064

6510 0.366

6511 0.025

6518 0.117

6519 0.109

6520 0.163

6522 0.032

6524 0.053

6525 0.107

6527 0.138

6533 0.591

6534 0.036

6535 0.008

6540 0.393

6541 0.074

6543 0.209

6545 0.104

6547 0.013

6548 0.037

6549 0.052

6553 0.024

6556 0.074

6558 0.029

6560 0.02

6561 0.11

6566 0.077

6569 0.052

6570 0.101

6573 0.27

6574 0.051

6575 0.042

6579 0.173

6581 0.077

6582 0.092

6583 0.083

6584 0.086

6585 0.032

6586 0.034

6590 0.311

6591 0.022

6596 0.095

6597 0.005

6601 0.051

6602 0.003

6603 0.039

6604 0.002

6605 0.013

6608 0.036

6613 0

6614 0.184

6615 0.075

6616 0

6617 0.135

6618 0.236

6619 0.085

6620 0.018

6624 0.02

6626 0.017

6627 0.011

6634 0

6635 0

6636 0

6638 0

6640 0.042

6641 0.03

6642 0.029

6643 0.011

6645 0.017

6646 0.11

6647 0.109

6648 0.053

6649 0.236

6651 0.077

6653 0.038

6659 0.067

6660 0.017

6662 0.014

6663 0.007

6666 0.026

6667 0.022

6668 0.034

6670 0.014

6672 0.472

6674 0.191

6676 0.099

6677 0.27

6678 0.04

6683 0.051

6687 0.049

6688 0.06

6690 0.237

6691 0.364

6692 0.12

6694 0.197

6696 0.227

6697 0.176

6709 0.341

6710 0.054

6712 0.028

6714 0.005

6715 0.267

6718 0.12

6720 0.109

6721 0.07

6723 0.029

6726 0.038

6727 0.125

6728 0.02

6729 0

6730 0.024

6731 0.027

6733 0.044

6734 0.014

6737 0.208

6738 0.061

6742 0.041

6744 0.11

6745 0.024

6746 0.016

6747 0.008

6748 0.025

6749 0.012

6750 0.017

6751 0.007

6753 0.081

6754 0.063

6757 0.396

6757 0.407

6760 0.021

6764 0.016

6767 0.028

6768 0.106

6769 0.094

6770 0.073

6772 0.036

6774 0.002

6775 0.029

6776 0.018

6777 0.016

6778 0.077

6780 0.096

6781 0.019

6782 0.048

6783 0.135

6785 0.046

6786 0.017

6788 0.017

6789 0.013

6790 0.095

6792 0.019

6793 0.064

6794 0.055

6795 0.135

6801 0.02

6804 0.013

6809 0.087

6811 0.032

6812 0

6815 0.019

6818 0.171

6819 0.133

6820 0.159

6827 0

6829 0.012

6832 0.071

6839 0.025

6840 0.108

6843 0.009

6844 0.008

6850 0.044

6854 0.023

6856 0.107

6857 0.012

6860 0.059

6861 0.05

6862 0.358

6863 0.024

6865 0.076

6866 0.226

6867 0.157

6871 0.012

6873 0.012

6874 0.027

6877 0.026

6879 0.025

6882 0.082

6883 0.018

6885 0.004

6887 0.098

6890 0.194

6891 0.147

6892 0.157

6894 0.171

6897 0.031

6899 0.031

6900 0.053

6901 0.015

6902 0.043

6904 0.117

6906 0.179

6907 0.028

6910 0.021

6913 0.006

6916 0.117

6917 0.016

6920 0.044

6923 0.016

6924 0.093

6925 0.009

6932 0.039

6934 0.067

6935 0.071

6936 0.186

6938 0.024

6939 0.126

6940 0.071

6941 0.12

6942 0.039

6943 0.022

6945 0.028

6948 0.183

6950 0.019

6954 0.14

6975 0.034

6990 0.053

6991 0.104

7003 0.004

7004 0.032

7005 0.027

7008 0.037

7009 0.037

7011 0.157

7014 0.08

7015 0.215

7018 0.177

7021 0

7022 0.06

7025 0.266

7027 0.025

7030 0.021

7031 0.234

7033 0.23

7036 0.097

7039 0.04

7040 0.063

7043 0.012

7044 0.11

7045 0.051

7047 0.359

7051 0.053

7052 0.104

7056 0.229

7058 0.069

7060 0.042

7064 0.066

7067 0.015

7068 0.018

7069 0.09

7070 0.227

7071 0.093

7072 0.021

7073 0.004

7075 0.04

7076 0.164

7077 0.009

7078 0.018

7080 0.009

7083 0.069

7086 0.03

7088 0.011

7089 0.043

7090 0.006

7091 0.006

7092 0.033

7093 0.057

7094 0.009

7095 0.03

7096 0.179

7097 0.199

7101 0.006

7102 0.008

7103 0.218

7104 0.072

7105 0.04

7106 0.035

7107 0.04

7109 0.004

7111 0.015

7112 0.042

7113 0.149

7122 0.073

7125 0.006

7126 0.014

7128 0.056

7133 0.254

7135 0.01

7136 0.015

7138 0.02

7139 0.064

7140 0.059

7145 0.049

7150 0.016

7153 0.059

7156 0.076

7157 0.139

7158 0.104

7162 0.089

7164 0.051

7165 0.044

7169 0.003

7170 0.005

7171 0.124

7172 0.118

7174 0.031

7179 0.295

7184 0.02

7185 0.079

7186 0.078

7187 0.021

7188 0.119

7189 0.064

7200 0.282

7203 0.015

7204 0.005

7205 0.076

7216 0.141

7222 0.035

7223 0.015

7224 0.016

7225 0.037

7226 0.089

7227 0.035

7247 0.009

7248 0.071

7249 0.05

7257 0.049

7259 0.258

7262 0.269

7263 0.06

7266 0.015

7272 0.142

7273 0.017

7275 0.02

7276 0.115

7277 0

7278 0

7283 0.006

7286 0.06

7287 0.106

7289 0.188

7290 0.022

7292 0.393

7296 0.057

7297 0.118

7298 0.081

7306 0.086

7307 0.061

7311 0.004

7314 0.027

7316 0.023

7317 0.024

7318 0.164

7319 0.003

7320 0

7321 0

7322 0

7323 0

7324 0.008

7325 0.005

7326 0.014

7327 0.008

7328 0

7329 0.003

7332 0

7334 0

7336 0.006

7341 0

7342 0.034

7343 0.01

7345 0.025

7347 0.008

7348 0.053

7349 0.085

7350 0.125

7351 0.024

7352 0.079

7356 0.304

7357 0.011

7358 0.011

7363 0.238

7366 0.234

7368 0.037

7372 0.068

7373 0.051

7374 0.077

7376 0.052

7378 0.132

7380 0.088

7381 0.073

7385 0.079

7386 0.064

7389 0.041

7390 0.134

7391 0.012

7392 0.012

7398 0.063

7399 0.183

7401 0.102

7403 0.014

7404 0.083

7405 0.066

7407 0.586

7409 0.043

7412 0.146

7414 0.005

7415 0.001

7416 0.008

7419 0.008

7422 0.054

7424 0.069

7425 0.082

7430 0.018

7431 0.012

7432 0.11

7433 0.082

7434 0.072

7436 0.021

7439 0.202

7441 0.162

7443 0.079

7444 0.129

7450 0.106

7455 0.336

7464 0.091

7465 0.051

7466 0.075

7468 0.084

7469 0.027

7471 0.008

7472 0.018

7473 0.007

7474 0.01

7475 0.011

7476 0.157

7477 0.005

7478 0.107

7480 0.019

7481 0.014

7482 0.006

7483 0.01

7484 0.035

7486 0.181

7490 0.013

7504 0.107

7507 0.073

7511 0.027

7514 0.012

7516 0.134

7517 0.178

7518 0.177

7520 0.126

7528 0.008

7529 0.006

7532 0

7535 0.035

7536 0.066

7539 0.174

7541 0.005

7542 0.04

7544 0.045

7546 0.007

7547 0.022

7549 0.133

7552 0.01

7553 0.203

7554 0.153

7555 0

7556 0.566

7559 0.168

7561 0.371

7562 0.432

7564 0.324

7566 0.136

7569 0.08

7570 0.089

7572 0.03

7574 0.425

7576 0.581

7579 0.14

7580 0.023

7586 0.304

7587 0.523

7589 0.547

7592 0.537

7593 0.098

7596 0.526

7597 0.045

7621 0.302

7629 0.041

7638 0.604

7691 0.516

7693 0.584

7699 0.504

7701 0.078

7702 0.016

7703 0.028

7704 0.02

7710 0.436

7711 0.635

7716 0.007

7726 0.062

7727 0.146

7728 0.336

7732 0.069

7733 0.067

7741 0.186

7743 0.035

7748 0.532

7750 0.022

7753 0.12

7755 0.155

7760 0.17

7762 0.549

7763 0.007

7764 0.181

7767 0.57

7771 0.244

7776 0.074

7780 0.12

7782 0.041

7784 0.23

7786 0.024

7789 0.263

7791 0.065

7799 0.091

7802 0.061

7804 0.054

7805 0.137

7809 0.178

7812 0.008

7813 0.047

7827 0.079

7837 0.049

7840 0.172

7844 0.022

7849 0.009

7850 0.288

7851 0.089

7855 0.018

7857 0.101

7867 0.028

7869 0.067

7871 0.053

7879 0

7881 0.086

7903 0.014

7905 0.031

7913 0.049

7915 0.09

7917 0.029

7920 0.021

7922 0.08

7932 0.071

7941 0.226

7942 0.035

7957 0.056

7976 0.008

7980 0.37

7982 0.005

7984 0.245

7988 0.123

7994 0.055

8001 0.127

8013 0.034

8021 0.126

8022 0.261

8027 0.04

8028 0.046

8029 0.203

8034 0.043

8036 0.008

8038 0.107

8048 0.01

8050 0.084

8061 0.045

8065 0.023

8073 0

8074 0.207

8076 0.107

8082 0.095

8085 0.036

8086 0.036

8089 0.004

8099 0.011

8100 0.056

8106 0.01

8110 0.008

8111 0.054

8115 0.379

8128 0.015

8139 0.022

8140 0.046

8148 0.024

8153 0.007

8161 0.22

8165 0.192

8170 0.046

8174 0.425

8175 0.018

8178 0.104

8187 0.256

8189 0.086

8192 0.094

8195 0.135

8200 0.039

8202 0.081

8208 0.095

8209 0.072

8216 0.031

8224 0.042

8241 0.02

8270 0.388

8277 0.192

8287 0.054

8289 0.027

8291 0.035

8292 0.068

8295 0.006

8302 0.63

8309 0.169

8313 0.052

8315 0.047

8317 0.115

8321 0.031

8322 0.018

8323 0.092

8324 0.017

8325 0.017

8326 0.022

8328 0.083

8332 0.013

8338 0

8347 0

8351 0.004

8352 0.003

8353 0.007

8368 0.005

8379 0.116

8382 0.088

8383 0.105

8390 0.365

8392 0.519

8394 0.048

8395 0.034

8396 0.005

8398 0.057

8405 0

8408 0.059

8409 0.052

8411 0.084

8412 0.08

8416 0.307

8424 0.06

8425 0.059

8428 0.011

8431 0.127

8433 0.278

8434 0.034

8435 0.103

8436 0.099

8437 0.084

8443 0.114

8444 0.057

8445 0.014

8446 0.187

8448 0.024

8449 0.026

8450 0.014

8451 0.024

8452 0.003

8454 0.001

8455 0.049

8458 0.19

8459 0.03

8462 0.149

8463 0.035

8464 0.043

8467 0.013

8468 0.084

8470 0.046

8471 0.117

8473 0.002

8476 0.031

8477 0.131

8482 0.059

8483 0.051

8484 0.046

8487 0.043

8490 0.053

8491 0.022

8492 0.107

8493 0.057

8495 0.057

8496 0.059

8497 0.028

8498 0.114

8499 0.009

8500 0.024

8501 0.093

8502 0.028

8504 0.031

8505 0.076

8507 0.004

8509 0.018

8513 0.139

8514 0.006

8515 0.07

8517 0.07

8518 0.118

8520 0.034

8521 0.154

8522 0.033

8527 0.033

8528 0.113

8531 0.056

8535 0.051

8537 0.245

8538 0.045

8539 0.009

8541 0.012

8542 0.555

8543 0

8546 0.063

8548 0.14

8549 0.084

8550 0.015

8553 0.05

8554 0.011

8555 0.066

8556 0.067

8557 0.052

8558 0.021

8560 0.09

8562 0.017

8563 0.025

8564 0.101

8565 0.019

8566 0.143

8567 0.03

8568 0.202

8569 0.036

8570 0.031

8572 0.049

8573 0.009

8574 0.069

8576 0.042

8577 0.045

8581 0.358

8601 0.115

8602 0.108

8603 0.043

8607 0.003

8608 0.22

8609 0.014

8612 0.054

8613 0.028

8614 0.074

8618 0.005

8621 0.026

8622 0.037

8625 0.083

8626 0.01

8629 0.134

8630 0.183

8631 0.067

8632 0.028

8633 0.017

8635 0.211

8636 0.013

8638 0.632

8642 0.041

8643 0.047

8645 0.061

8646 0.03

8647 0.11

8648 0.038

8649 0.012

8650 0.03

8653 0.062

8658 0.011

8659 0.046

8660 0.083

8662 0.042

8663 0

8665 0.036

8666 0.009

8667 0.016

8669 0.011

8671 0.037

8672 0.071

8673 0.053

8674 0.041

8675 0.011

8676 0.084

8678 0.008

8681 0.11

8682 0.057

8685 0.238

8689 0.12

8693 0.048

8694 0.076

8697 0.025

8698 0.115

8701 0.089

8702 0.102

8703 0.017

8704 0.043

8705 0.125

8706 0.034

8708 0.004

8710 0.153

8711 0.113

8714 0.118

8715 0.034

8717 0.138

8721 0.01

8722 0.137

8723 0.038

8724 0

8725 0.152

8726 0

8727 0.068

8728 0.089

8729 0.033

8731 0.141

8732 0.022

8733 0.06

8735 0.009

8736 0.064

8738 0.051

8740 0.146

8743 0.217

8745 0.033

8748 0.398

8749 0.245

8751 0.12

8754 0.086

8756 0.207

8760 0.022

8761 0.012

8764 0.471

8766 0

8767 0.091

8773 0.068

8776 0.035

8777 0.091

8780 0.031

8784 0.317

8785 0.054

8786 0.113

8787 0.049

8790 0.164

8792 0.197

8795 0.586

8796 0.173

8797 0.576

8798 0.155

8800 0.132

8804 0.145

8805 0.031

8807 0.221

8808 0.229

8811 0.096

8812 0.011

8813 0.055

8815 0.017

8816 0.049

8817 0.005

8818 0.072

8819 0.022

8820 0.115

8822 0.008

8824 0.242

8825 0.002

8828 0.03

8829 0.047

8832 0.272

8833 0.011

8834 0.013

8836 0.222

8837 0.231

8838 0.123

8840 0.102

8841 0.001

8842 0.285

8846 0.111

8848 0.035

8850 0.038

8851 0.008

8853 0.31

8854 0.014

8856 0.122

8861 0.002

8863 0.231

8864 0.118

8867 0.039

8869 0.094

8871 0.095

8874 0.04

8876 0.152

8877 0.117

8878 0.05

8879 0.09

8880 0.007

8881 0.028

8882 0.053

8883 0.018

8884 0.107

8887 0.06

8888 0.129

8893 0.071

8895 0.036

8897 0.08

8898 0.008

8899 1.002

8904 0.049

8905 0.023

8906 0.074

8910 0.02

8911 0.048

8912 0.084

8913 0.031

8914 0.094

8915 0.05

8924 0.025

8927 0.056

8929 0.001

8932 0.013

8935 0.052

8936 0.011

8938 0.066

8939 0.038

8941 0.027

8943 0.051

8945 0.006

8971 0.144

8972 0.127

8973 0.092

8974 0.043

8976 0.021

8985 0.048

8986 0.022

8987 0.217

8991 0.08

8994 0.133

8995 0.338

8996 0.091

8997 0.02

8997 0.063

9001 0.219

9013 0.23

9019 0.093

9021 0.017

9023 0.123

9024 0.007

9025 0.188

9026 0.054

9027 0.284

9031 0.052

9032 0.102

9033 0.077

9034 0.359

9038 0.08

9043 0.028

9044 0.019

9045 0.07

9046 0.154

9047 0.193

9048 0.134

9050 0.084

9052 0.154

9053 0.112

9054 0.044

9055 0.091

9058 0.134

9060 0.048

9061 0.023

9063 0.011

9064 0.074

9066 0.014

9068 0.035

9069 0.05

9071 0.024

9073 0.101

9074 0.068

9075 0.048

9076 0.052

9079 0.016

9085 0.53

9086 0.01

9092 0.022

9093 0.073

9094 0.047

9095 0.04

9096 0.016

9097 0.015

9098 0.051

9099 0.058

9104 0.067

9107 0.051

9108 0.03

9110 0.045

9112 0.022

9113 0.039

9114 0.002

9118 0.032

9121 0.246

9124 0.043

9125 0.006

9126 0.001

9127 0.167

9128 0.007

9129 0.003

9130 0.013

9131 0.074

9132 0.019

9133 0.056

9134 0.042

9136 0.039

9138 0.062

9139 0.016

9140 0.064

9141 0.02

9145 0.034

9146 0.038

9147 0.074

9148 0.033

9149 0.014

9150 0.13

9152 0.029

9156 0.165

9158 0.014

9159 0.074

9166 0.018

9167 0.053

9169 0.184

9170 0.046

9173 0.25

9175 0.064

9177 0.154

9181 0.115

9182 0.089

9184 0

9185 0.065

9187 0.173

9188 0.096

9191 0.006

9194 0.177

9197 0.033

9200 0.06

9201 0.008

9205 0.206

9208 0.063

9209 0.101

9210 0.234

9211 0.014

9212 0.097

9214 0.28

9215 0.013

9217 0.053

9219 0.008

9223 0.021

9227 0.117

9228 0.063

9229 0.02

9232 0.169

9236 0.179

9238 0.157

9240 0.044

9242 0.075

9245 0.15

9247 0.205

9249 0.034

9252 0.017

9253 0.022

9254 0.02

9255 0.074

9256 0.106

9258 0.024

9260 0.047

9261 0.024

9263 0.295

9265 0.006

9267 0.01

9271 0.028

9275 0.028

9276 0.008

9277 0.084

9278 0.072

9282 0.025

9287 0.061

9289 0.13

9290 0.142

9294 0.062

9295 0.007

9306 0.099

9308 0.21

9312 0.037

9314 0.043

9317 0.072

9318 0

9322 0.05

9325 0.062

9326 0.11

9328 0.084

9329 0.04

9330 0.033

9331 0.026

9332 0.173

9333 0.095

9334 0.028

9337 0.005

9338 0.092

9340 0.13

9342 0.097

9344 0.006

9348 0.039

9349 0

9350 0.21

9351 0.041

9352 0.009

9353 0.018

9355 0.006

9356 0.078

9358 0.106

9361 0.069

9362 0.013

9365 0.085

9367 0.023

9368 0.064

9369 0.326

9370 0.114

9371 0.01

9372 0.108

9373 0.033

9375 0.022

9376 0.139

9377 0.079

9378 0.002

9379 0.01

9381 0.133

9382 0.081

9388 0.117

9391 0.028

9392 0.061

9394 0.02

9397 0.022

9399 0.063

9400 0.133

9401 0.232

9403 0.072

9404 0.09

9406 0.015

9409 0.051

9411 0.104

9412 0.01

9413 0.076

9414 0.085

9415 0.07

9416 0.008

9419 0.007

9420 0.217

9421 0.042

9422 0.332

9424 0.115

9425 0.083

9426 0.253

9427 0.028

9429 0.116

9435 0.026

9439 0.027

9440 0.023

9442 0.01

9443 0.032

9444 0

9445 0.025

9446 0.191

9448 0.012

9450 0.195

9451 0.07

9453 0.021

9456 0.012

9459 0.046

9462 0.015

9463 0.004

9466 0.256

9468 0.023

9469 0.078

9470 0.009

9472 0.117

9473 0.256

9474 0.016

9475 0.019

9476 0.187

9477 0.017

9478 0.027

9479 0.03

9481 0.023

9482 0.035

9486 0.057

9489 0.027

9491 0.097

9493 0.059

9496 0.036

9497 0.031

9500 0.057

9507 0.055

9508 0.046

9509 0.064

9510 0.101

9512 0.06

9514 0.093

9515 0.025

9517 0.02

9518 0.258

9519 0

9520 0.01

9521 0.077

9522 0.01

9525 0.025

9527 0.014

9528 0.032

9529 0.052

9530 0.079

9531 0.076

9533 0.031

9534 0.496

9538 0.019

9541 0.005

9542 0.017

9543 0.071

9545 0.028

9546 0.153

9547 0.049

9551 0.116

9552 0.074

9553 0.13

9555 0.008

9557 0.078

9559 0.006

9562 0.105

9563 0.098

9564 0.048

9567 0.013

9568 0.027

9570 0.066

9572 0.022

9573 0.188

9575 0.019

9576 0.02

9577 0.005

9578 0.046

9580 0.055

9581 0.039

9583 0.037

9584 0

9585 0.161

9586 0.008

9589 0.018

9590 0.277

9592 0.138

9595 0.117

9600 0.035

9601 0.061

9603 0.178

9604 0.048

9607 0.017

9609 0.128

9611 0.075

9612 0.071

9615 0.047

9616 0.03

9617 0.108

9618 0.031

9620 0.111

9622 0.216

9625 0.25

9627 0.072

9628 0.017

9629 0.187

9630 0.021

9631 0.029

9632 0.033

9633 0.149

9635 0.175

9637 0.062

9644 0.052

9645 0.055

9646 0.015

9648 0.123

9649 0.017

9650 0.125

9651 0.07

9652 0.089

9653 0.016

9655 0.033

9662 0.105

9665 0.083

9666 0.093

9669 0.093

9670 0.005

9671 0.037

9672 0.092

9673 0.023

9675 0.09

9677 0.055

9678 0.015

9681 0.027

9682 0.035

9683 0.107

9684 0.038

9686 0.044

9687 0.077

9688 0.014

9689 0

9692 0.127

9693 0.031

9694 0.013

9696 0.095

9698 0.005

9699 0.07

9701 0.1

9702 0.071

9705 0.085

9706 0.035

9709 0.064

9710 0.033

9715 0.025

9716 0.028

9717 0.074

9718 0.042

9720 0.318

9722 0.034

9723 0.078

9725 0.058

9726 0.182

9728 0.063

9730 0.008

9731 0.118

9732 0.01

9733 0.077

9734 0.047

9736 0.019

9737 0.255

9739 0.018

9741 0.015

9742 0.098

9743 0.046

9745 0.063

9746 0.025

9747 0.056

9748 0.095

9749 0.1

9750 0.059

9751 0.046

9753 0.234

9755 0.03

9757 0.046

9758 0.075

9759 0.032

9760 0.028

9762 0.024

9764 0.057

9765 0.165

9766 0.055

9768 0.092

9770 0.046

9776 0.033

9782 0.008

9785 0.014

9786 0.18

9787 0.277

9788 0.017

9791 0.014

9796 0.04

9798 0.026

9810 0.041

9811 0.035

9813 0.106

9814 0.247

9815 0.042

9817 0.037

9818 0.043

9819 0.017

9820 0.128

9821 0.058

9823 0.144

9825 0.085

9826 0.079

9828 0.071

9829 0.045

9830 0.136

9831 0.117

9832 0.004

9836 0.216

9839 0.017

9841 0.082

9842 0.097

9843 0.082

9844 0.003

9846 0.034

9847 0.03

9848 0.056

9849 0.137

9851 0.148

9852 0.055

9853 0.069

9854 0.051

9855 0.098

9856 0.143

9857 0.115

9859 0.041

9860 0.054

9861 0.015

9862 0.035

9863 0.008

9865 0.063

9866 0.074

9867 0.094

9868 0.035

9870 0.709

9871 0.069

9873 0.018

9874 0.011

9875 0.157

9877 0.116

9878 0.024

9879 0.006

9880 0.058

9881 0.087

9882 0.095

9883 0.184

9885 0.051

9886 0.069

9887 0.043

9889 0.101

9890 0.022

9891 0.052

9892 0.029

9894 0.16

9895 0.102

9896 0.029

9899 0.028

9900 0.01

9901 0.013

9902 0.054

9903 0.01

9904 0.105

9905 0.043

9907 0.14

9908 0.009

9909 0.047

9910 0.038

9911 0.022

9912 0.034

9913 0.023

9914 0.107

9915 0.014

9917 0.014

9918 0.075

9919 0.731

9920 0.11

9921 0.06

9922 0.27

9923 0.125

9924 0.018

9925 0.029

9933 0.058

9935 0.013

9939 0.003

9940 0.186

9941 0.104

9943 0.151

9945 0.012

9946 0.051

9947 0.622

9948 0.027

9949 0.01

9950 0.088

9951 0.154

9953 0.067

9955 0.084

9956 0.031

9957 0.068

9958 0.012

9960 0.027

9961 0.059

9962 0.026

9963 0.051

9965 0.379

9966 0.204

9967 0.032

9968 0.015

9969 0.033

9972 0.097

9973 0.077

9978 0

9980 0.091

9982 0.254

9984 0.021

9985 0.139

9986 0.028

9987 0.007

9988 0.026

9989 0.076

9990 0.01

9991 0.046

9992 0.087

10000 0.009

10001 0.025

10002 0.053

10003 0.078

10004 0.112

10005 0.083

10006 0.009

10009 0.061

10010 0.104

10013 0.142

10014 0.021

10015 0.029

10016 0.007

10017 0.409

10018 0.051

10019 0.131

10020 0.008

10021 0.023

10022 0.304

10023 0.13

10025 0.048

10026 0.055

10038 0.058

10039 0.116

10040 0.103

10042 0.072

10043 0.061

10044 0.048

10046 1.12

10047 0.289

10048 0.028

10049 0.023

10050 0.121

10051 0.059

10053 0.022

10054 0.035

10056 0

10057 0.031

10058 0.063

10059 0.008

10061 0.008

10066 0.072

10068 0.274

10072 0.042

10075 0.015

10076 0.04

10077 0.273

10079 0.01

10081 0.054

10082 0.023

10083 0.053

10085 0.022

10087 0.03

10089 0.127

10090 0.021

10096 0.001

10097 0

10098 0

10099 0.019

10100 0.032

10101 0.092

10102 0.085

10103 0.184

10105 0.056

10106 0.025

10107 0.097

10109 0.003

10110 0.035

10113 0.064

10114 0.052

10116 0.007

10117 0.233

10120 0.006

10123 0

10125 0.052

10126 0.091

10127 0.088

10130 0.028

10131 0.069

10133 0.12

10134 0.065

10136 0.165

10138 0.066

10140 0.02

10142 0.186

10144 0.104

10146 0.031

10147 0.157

10148 0.248

10149 0.111

10152 0.011

10154 0.068

10155 0.032

10156 0.086

10157 0.075

10159 0.031

10160 0.048

10162 0.073

10163 0.043

10164 0.178

10165 0.021

10166 0.033

10171 0.019

10172 0.433

10174 0.101

10175 0.003

10178 0.02

10179 0.086

10180 0.058

10181 0.011

10186 0.024

10189 0.018

10190 0.058

10193 0.002

10194 0.04

10195 0.055

10196 0.055

10197 0

10198 0.089

10199 0.104

10201 0.051

10205 0.1

10206 0.021

10207 0.113

10208 0.239

10209 0.004

10210 0.072

10211 0.008

10213 0

10214 0.536

10215 0.018

10218 0.062

10219 0.31

10220 0.001

10221 0.031

10224 0.633

10225 0.26

10226 0.151

10232 0.314

10233 0.131

10235 0.034

10236 0.002

10238 0.001

10239 0

10240 0.209

10241 0.231

10243 0.001

10244 0.126

10245 0.02

10247 0.107

10249 0.161

10250 0.028

10251 0.467

10252 0.081

10253 0.017

10254 0.077

10256 0.142

10257 0.081

10260 0.033

10265 0.033

10268 0.086

10269 0.036

10270 0.122

10272 0.087

10273 0.01

10276 0.079

10277 0.014

10278 0.118

10279 0.135

10280 0.062

10285 0.006

10289 0

10290 0.029

10291 0.008

10293 0.12

10294 0.002

10296 0.071

10298 0.039

10299 0.009

10300 0.026

10302 0.111

10307 0.067

10311 0.047

10312 0.097

10313 0.026

10316 0.178

10317 0.182

10318 0.087

10319 0.162

10320 0.035

10322 0.054

10324 0.022

10325 0.013

10327 0.037

10328 0.038

10330 0.016

10331 0.277

10332 0.407

10333 0.168

10335 0.094

10346 0.312

10347 0.142

10350 0.131

10351 0.158

10352 0.074

10360 0.072

10361 0.205

10362 0.036

10363 0.02

10365 0.648

10367 0.041

10368 0.01

10369 0.006

10382 0.005

10383 0.001

10385 0.197

10388 0.144

10389 0.264

10390 0.007

10391 0.009

10392 0.118

10393 0.003

10394 0.277

10395 0.04

10396 0.011

10397 0.033

10398 0.003

10399 0

10400 0.123

10401 0.019

10402 0.159

10403 0.11

10404 0.065

10411 0.054

10412 0.004

10413 0.044

10417 0.09

10418 0.021

10419 0.01

10420 0.034

10421 0.048

10423 0.02

10425 0.008

10426 0.033

10427 0.09

10428 0.091

10432 0.009

10434 0.043

10435 0.068

10439 0.326

10440 0.019

10446 0.05

10449 0.072

10450 0.009

10451 0.023

10454 0.029

10457 0.175

10458 0.026

10459 0.009

10460 0.247

10462 0.352

10463 0.057

10464 0.064

10465 0

10466 0.058

10467 0.007

10468 0.011

10469 0.059

10472 0.004

10474 0.005

10476 0.126

10478 0.024

10479 0.019

10480 0.006

10482 0.046

10483 0.013

10484 0.005

10486 0.06

10487 0.023

10488 0.211

10489 0.022

10491 0.058

10492 0.002

10493 0.048

10494 0.006

10495 0.033

10497 0.03

10498 0.005

10499 0.029

10500 0.067

10501 0.059

10505 0.046

10507 0.644

10509 0.098

10512 0.016

10514 0.202

10518 0.309

10520 0.605

10521 0.007

10522 0.049

10524 0.001

10525 0.042

10526 0.047

10527 0.003

10528 0.055

10529 0.078

10531 0.077

10533 0.037

10538 0.023

10542 0.179

10549 0.055

10550 0.053

10551 0.049

10552 0.008

10553 0.089

10556 0.051

10559 0.045

10560 0.056

10561 0.363

10562 0.194

10563 0.464

10564 0.025

10565 0.015

10566 0.163

10568 0.136

10570 0.036

10571 0.24

10572 0.177

10574 0.025

10575 0.018

10576 0.014

10577 0.14

10579 0.08

10580 0.068

10584 0.065

10585 0.086

10587 0.095

10588 0.08

10590 0.088

10591 0.166

10592 0.047

10594 0.001

10595 0.11

10597 0.025

10598 0.027

10599 0.633

10602 0.044

10603 0.076

10606 0.041

10607 0.064

10608 0.046

10609 0.05

10610 0.166

10611 0.068

10612 0.011

10613 0.025

10614 0.074

10615 0.205

10616 0.048

10620 0.064

10621 0.009

10623 0.035

10625 0.004

10627 0.011

10629 0.037

10630 0.421

10632 0.101

10633 0.028

10634 0.066

10636 0.083

10637 0.118

10640 0.011

10641 0.009

10642 0.004

10643 0.018

10644 0.022

10645 0.043

10648 0.474

10651 0.018

10652 0.036

10654 0.095

10656 0.013

10658 0.003

10659 0.002

10660 0.014

10661 0.161

10667 0.088

10668 0.092

10670 0

10671 0.019

10672 0.02

10673 0.21

10675 0.072

10677 0.059

10678 0.076

10681 0.004

10683 0.102

10686 0.131

10687 0.132

10690 0.004

10691 0.023

10692 0.114

10693 0.1

10694 0.019

10699 0.099

10712 0.03

10713 0.009

10715 0.117

10718 0.064

10720 0.24

10721 0.209

10723 0.055

10725 0.042

10730 0.031

10732 0.061

10733 0.099

10734 0.413

10735 0.005

10736 0.006

10741 0.039

10742 0.055

10745 0.036

10746 0.032

10748 0.525

10752 0.093

10753 0.083

10755 0.019

10758 0.101

10761 0.312

10762 0.124

10763 0.151

10765 0.033

10766 0.051

10767 0.061

10768 0

10769 0.016

10771 0.023

10772 0

10773 0.04

10775 0.112

10776 0.004

10777 0.088

10778 0.083

10780 0.385

10781 0.35

10782 0.282

10783 0.026

10785 0.152

10787 0.005

10791 0.178

10793 0.366

10797 0.045

10798 0.054

10799 2.832

10800 0.068

10801 0.046

10802 0.062

10804 0.025

10806 0.128

10809 0.028

10810 0.032

10813 0.135

10814 0.039

10815 0.021

10818 0.024

10820 0.224

10825 0.229

10827 0.114

10838 0.066

10841 0.093

10842 0.117

10844 0.039

10845 0.022

10846 0.029

10847 0.063

10850 0.248

10855 0.129

10858 0.026

10859 0.551

10861 0.143

10863 0.184

10865 0.155

10867 0.018

10868 0.328

10869 0.058

10871 0.455

10879 0.704

10880 0.074

10881 0.086

10882 0

10885 0.052

10886 0.119

10887 0.092

10888 0.063

10891 0.027

10892 0.068

10893 0.016

10894 0.202

10898 0.085

10899 0.082

10900 0.019

10901 0.121

10902 0.028

10903 0.07

10904 0

10905 0.032

10906 0.14

10907 0.003

10910 0.075

10911 0.416

10913 0.044

10914 0.02

10915 0.012

10916 0.072

10919 0.023

10920 0.04

10921 0.002

10923 0.036

10924 0.117

10926 0.222

10929 0.129

10930 0.047

10933 0.008

10935 0.072

10936 0.063

10938 0.004

10940 0.142

10941 0.07

10942 0.258

10943 0.07

10944 0.021

10945 0.002

10946 0.004

10947 0.009

10948 0.035

10949 0.035

10950 0.028

10951 0

10952 0.005

10955 0.135

10956 0.115

10957 0.084

10959 0.005

10962 0.07

10963 0.016

10964 0.258

10969 0.108

10970 0.132

10971 0.002

10972 0.039

10973 0.043

10979 0.009

10981 0.084

10982 0.017

10983 0.03

10987 0.003

10988 0.029

10990 0.419

10991 0.065

10992 0.013

10994 0.08

10998 0.196

10999 0.332

11000 0.059

11001 0.099

11004 0.067

11005 0.238

11009 0.263

11010 0.184

11011 0.003

11012 0.171

11015 0.046

11016 0.013

11020 0.089

11021 0.002

11022 0.048

11023 0.083

11024 0.523

11030 0.022

11031 0.029

11033 0.039

11034 0.021

11035 0.294

11036 0.168

11040 0.062

11041 0.02

11044 0.032

11045 0.035

11054 0.331

11055 0.134

11056 0.036

11057 0.007

11059 0.048

11060 0.022

11061 0.089

11062 2.348

11063 0.125

11064 0.12

11065 0.02

11066 0.062

11067 0.291

11069 0.015

11074 0.337

11076 0.039

11077 0.067

11078 0.064

11079 0.017

11080 0.031

11081 0.077

11082 0.161

11083 0.138

11083 0.141

11085 0.333

11086 0.418

11091 0.108

11092 0.042

11095 0.109

11096 0.053

11097 0.076

11100 0.022

11101 0.137

11102 0.077

11103 0.055

11105 0.198

11107 0.039

11108 0.027

11113 0.019

11117 0.073

11122 0.009

11123 0.056

11124 0.023

11126 0.217

11127 0.014

11128 0.012

11130 0.312

11131 0.156

11132 0.102

11135 0.151

11136 0.077

11138 0.053

11141 0.007

11143 0.002

11144 0.015

11145 0.103

11146 0.08

11151 0.03

11152 0.013

11153 0.062

11154 0.112

11155 0.106

11156 0.024

11157 0.009

11158 0.095

11159 0.096

11160 0.015

11161 0.01

11162 0.121

11163 0.016

11164 0.099

11165 0.011

11167 0.038

11169 0.095

11171 0.011

11172 0.444

11173 0.125

11174 0.041

11177 0.071

11178 0.056

11179 0.05

11180 0.083

11181 0.109

11183 0.017

11185 0.391

11186 0.055

11187 0.037

11188 0.054

11189 0.002

11190 0.061

11191 0.014

11193 0.125

11194 0.106

11196 0.084

11197 0.037

11199 0.061

11201 0.134

11202 0.19

11212 0.078

11213 0.149

11214 0.079

11215 0.155

11216 0.052

11218 0.1

11219 0.067

11221 0.018

11224 0.012

11227 0.154

11228 0.051

11231 0.018

11232 0.127

11234 0.106

11235 0.002

11236 0.032

11238 0.059

11240 0.042

11244 0.047

11245 0.09

11247 0.062

11248 0.019

11249 0.061

11250 0.061

11251 0.127

11254 0.06

11255 0.03

11258 0.032

11260 0.026

11261 0.005

11264 0.118

11266 0.103

11267 0.007

11269 0.014

11270 0.033

11274 0.191

11275 0.01

11276 0.068

11277 0.78

11278 0.014

11280 0.173

11282 0.01

11283 0.206

11284 0.119

11309 0.159

11311 0.016

11314 0.432

11317 0.067

11320 0.036

11321 0.05

11322 0.15

11325 0.02

11329 0.007

11330 0.138

11331 0.002

11332 0.02

11333 0.018

11335 0

11336 0.021

11338 0.016

11339 0.171

11340 0.048

11341 0.1

11342 0.036

11343 0.093

11345 0

11346 0.113

22795 0.124

22796 0.083

22797 0.147

22798 0.342

22801 0.056

22802 0.198

22803 0.022

22806 0.089

22807 0.01

22809 0.076

22818 0

22820 0.015

22822 0.031

22824 0.044

22826 0.008

22827 0.007

22828 0.045

22829 0.165

22830 0.089

22832 0.15

22835 0.093

22836 0.021

22837 0.196

22838 0.036

22839 0.038

22841 0.055

22843 0.052

22844 0.138

22846 0.036

22847 0.12

22848 0.041

22849 0.011

22850 0.115

22852 0.246

22853 0.135

22854 0.025

22856 0.031

22858 0.073

22859 0.006

22861 0.369

22862 0.05

22863 0.036

22865 0.021

22866 0.006

22868 0.211

22869 0.616

22870 0.083

22871 0.011

22872 0.044

22873 0.25

22874 0.041

22875 0.107

22876 0.053

22878 0.043

22880 0.054

22881 0.08

22883 0.044

22884 0.019

22885 0.012

22887 0.025

22888 0.123

22889 0.018

22890 0.023

22891 0.078

22893 0.05

22894 0.07

22895 0.058

22897 0.214

22898 0.131

22899 0.085

22901 0.111

22903 0.016

22904 0.104

22905 0.057

22906 0.085

22907 0.012

22911 0.025

22915 0.229

22916 0.015

22917 0.257

22918 0.214

22920 0.012

22921 0.152

22924 0.005

22925 0.163

22926 0.081

22928 0.06

22929 0.004

22931 0.006

22933 0.078

22934 0.031

22936 0.059

22938 0.009

22941 0.037

22941 0.042

22941 0.043

22944 0.044

22948 0.023

22949 0.096

22950 0.112

22953 0.089

22955 0.03

22974 0.122

22976 0.094

22978 0.003

22979 0.015

22980 0.06

22981 0.297

22982 0.006

22983 0.036

22985 0.077

22986 0.039

22987 0.015

22989 0.149

22990 0.052

22992 0.012

22993 0.078

22994 0.154

22995 0.215

22996 0.043

22998 0.088

22999 1.044

23001 0.02

23002 0.016

23004 0.15

23005 0.077

23007 0.018

23011 0.015

23012 0.013

23013 0.107

23014 0.026

23015 0.36

23016 0.02

23019 0.005

23020 0.003

23022 0.072

23023 0.02

23024 0.031

23025 0.023

23026 0.139

23028 0.009

23030 0.1

23031 0.059

23032 0.055

23033 0.02

23034 0.022

23035 0.052

23036 0.04

23037 0.206

23038 0.016

23039 0.003

23040 0.021

23041 0.028

23043 0.013

23045 0.043

23046 0.023

23047 0.021

23048 0.039

23049 0.066

23051 0.079

23052 0.144

23053 0.017

23054 0.044

23057 0.006

23060 0.034

23061 0.041

23062 0.045

23064 0.128

23066 0.054

23067 0.171

23071 0.037

23072 0.069

23074 0.088

23075 0.029

23076 0.228

23077 0.016

23078 0.082

23080 0.067

23081 0.095

23082 0.134

23085 0.194

23086 0.283

23087 0.249

23089 0.215

23090 0.008

23091 0.048

23093 0.059

23094 0.044

23095 0.017

23096 0.006

23097 0.021

23098 0.042

23099 0.037

23102 0.067

23105 0.093

23108 0.032

23109 0.165

23111 0.078

23112 0.21

23113 0.088

23114 0.155

23118 0.027

23119 0.064

23120 0.149

23122 0.018

23125 0.044

23126 0.035

23127 0.043

23129 0.046

23130 0.082

23131 0.078

23132 0.025

23133 0.028

23135 1.038

23136 0.459

23137 0.06

23138 0.049

23139 0.104

23140 0.062

23141 0.202

23142 0.011

23143 0.064

23144 0.192

23145 0.162

23145 0.168

23145 0.219

23148 0.254

23149 0.079

23150 0.035

23151 0.029

23152 0.039

23154 0.01

23157 0.02

23158 0.022

23161 0.014

23162 0.037

23163 0.068

23164 0.047

23174 0.076

23176 0.062

23177 0.159

23179 0.028

23180 0.177

23181 0.078

23184 0.067

23187 0.029

23189 0.083

23190 0.055

23191 0.027

23192 0.037

23193 0.047

23194 0.009

23195 0.097

23197 0.011

23200 0.035

23207 0.061

23208 0.023

23209 0.059

23210 0.013

23212 0.051

23213 0.04

23214 0.012

23215 0.068

23216 0.055

23217 0.38

23218 0.068

23219 0.027

23220 0.036

23221 0.015

23223 0.051

23224 0.132

23227 0.018

23228 0.02

23230 0.057

23232 0.084

23233 0.007

23234 0.067

23235 0.045

23236 0.017

23237 0.041

23239 0.044

23240 0.139

23241 0.031

23242 0.228

23243 0.01

23244 0.003

23245 0.012

23246 0.069

23246 0.106

23247 0.196

23248 0.046

23249 0.056

23250 0.036

23251 0.056

23252 0.095

23253 0.043

23256 0.013

23258 0.012

23259 0.068

23261 0.034

23262 0.028

23263 0.054

23264 0.023

23265 0.019

23266 0.021

23268 0.15

23269 0.075

23269 0.137

23270 0.042

23271 0.045

23275 0.047

23276 0.008

23277 0.036

23279 0.094

23281 0.117

23285 0.258

23286 0.042

23287 0.055

23291 0.006

23293 0.051

23299 0.023

23300 0.126

23301 0.062

23302 0.092

23303 0.072

23304 0.056

23305 0.06

23306 0.082

23307 0.12

23308 0.474

23309 0.379

23310 0.196

23312 0.039

23313 0.016

23314 0.003

23315 0.02

23316 0.097

23317 0.019

23321 0.005

23324 0.16

23325 0.02

23326 0.149

23327 0.014

23328 0.072

23329 0.092

23331 0.064

23332 0.012

23333 0.051

23334 0.039

23335 0.027

23336 0.175

23338 0.047

23339 0.015

23341 0.062

23344 0.067

23348 0.023

23351 0.155

23352 0.018

23353 0.208

23354 0.18

23355 0.068

23357 0.051

23359 0.162

23360 0.071

23361 0.056

23362 0.045

23362 0.396

23363 0.055

23365 0.059

23366 0.083

23367 0.025

23367 0.38

23368 0.047

23373 0.05

23376 0.052

23380 0.014

23381 0.036

23383 0.001

23385 0.062

23386 0.016

23387 0.044

23389 0.039

23390 0.007

23392 0.023

23395 0.074

23396 0.041

23397 0.128

23398 0.017

23400 0.082

23401 0.143

23404 0.033

23405 0.038

23409 0.077

23410 0.132

23411 0.067

23412 0.053

23413 0

23414 0.033

23415 0.049

23416 0.023

23418 0.147

23424 0.087

23426 0.035

23428 0.039

23429 0.005

23430 0.214

23431 0.117

23432 0.04

23433 0.011

23435 0.022

23436 0.103

23439 0.058

23440 0.001

23446 0.021

23450 0.002

23451 0.002

23452 0.028

23456 0.12

23457 0.035

23460 0.212

23463 0.023

23464 0.053

23466 0.05

23469 0.122

23471 0.035

23473 0.026

23474 0.054

23475 0.096

23476 0.022

23478 0.125

23479 0.011

23483 0.047

23492 0.062

23493 0.034

23499 0.305

23500 0.045

23504 0.079

23507 0.037

23509 0.046

23516 0.078

23517 0.013

23519 0.121

23521 0.026

23522 1.158

23523 0.04

23524 1.342

23526 0.099

23528 0.028

23529 0.015

23531 0.004

23532 0.507

23533 0.078

23534 0.003

23536 0.137

23539 0.077

23541 0.036

23542 0.062

23543 0.008

23544 0.088

23545 0.048

23546 0.164

23547 0.407

23548 0.104

23549 0.068

23550 0.19

23551 0.03

23553 0.117

23554 0.013

23556 0.075

23557 0.011

23559 0.092

23560 0.028

23562 0.048

23563 0.081

23566 0.051

23568 0.026

23569 0.162

23576 0.035

23580 0.112

23581 0.169

23583 0.061

23584 0.104

23585 0.035

23587 0.16

23588 0.019

23589 0.017

23592 0.069

23593 0.091

23594 0.137

23595 0.115

23596 0.07

23597 0.107

23598 0.031

23600 0.15

23601 0.163

23603 0.014

23604 0.014

23607 0.08

23608 0.031

23609 0.064

23612 0.004

23613 0.047

23617 0.595

23619 0.709

23620 0.135

23621 0.018

23623 0.087

23624 0.468

23626 0.096

23627 0.15

23629 0.449

23630 0.118

23632 0.096

23633 0.004

23635 0.001

23637 0.043

23639 0.11

23640 0.023

23643 0.251

23644 0.024

23645 0.396

23646 0.035

23647 0.004

23648 0

23650 0.059

23654 0.066

23657 0.06

23658 0

23659 0.067

23670 0.073

23671 0.006

23673 0.037

23676 0.08

23677 0.042

23678 0.021

23683 0.023

23705 0.053

23708 0.051

23710 0

23729 0.079

23731 0.049

23732 0.009

23743 0.088

23746 0.075

23753 0.066

23759 0.058

23760 0.011

23761 0.045

23762 0.056

23764 0.031

23765 0.203

23766 0.367

23769 0.02

23774 0.025

23786 0.148

23787 0.009

23788 0.038

24137 0.079

24139 0.257

24141 0.087

24145 0.071

24146 0.132

24147 0.056

24148 0.01

24149 0.099

25759 0.097

25763 0.378

25766 0.021

25769 0.058

25771 0.059

25777 0.096

25778 0.045

25780 0.029

25782 0.054

25788 0.118

25790 0.166

25791 0.083

25792 0.177

25793 0.166

25794 0.054

25796 0.062

25798 0.017

25799 0.334

25803 0.076

25804 0.017

25806 0.074

25813 0.022

25816 1.918

25817 0

25818 0.171

25819 0.054

25820 0.002

25821 0.079

25822 0.004

25824 0.068

25825 0.062

25827 0.022

25828 0.062

25830 0.023

25831 0.007

25833 0.038

25836 0.016

25839 0.026

25840 0.098

25841 0.017

25843 0

25849 0.242

25850 0.385

25851 0.074

25852 0.008

25853 0.007

25854 0.21

25855 0.037

25861 0.066

25862 0.042

25865 0.248

25870 0.088

25871 0.227

25875 0.116

25876 0.065

25878 0.787

25879 0.039

25880 0.201

25884 0.184

25886 0.052

25888 0.34

25890 0.171

25891 0.055

25893 0.584

25897 0.035

25898 0.053

25900 0.016

25901 0.109

25902 0.07

25903 0.093

25904 0.02

25906 0

25907 0.054

25909 0.11

25911 0.065

25912 0.023

25913 0.181

25920 0.021

25921 0.012

25923 0.029

25924 0.12

25925 0.015

25926 0.147

25928 0.03

25929 0.105

25930 0.048

25932 0.007

25934 0.149

25936 0.207

25937 0.041

25938 0.054

25938 0.067

25939 0.176

25942 0.011

25943 0.088

25945 0.04

25946 0.026

25949 0.05

25950 0.186

25953 0.026

25956 0.156

25957 0.023

25959 0.092

25960 0.066

25961 0.15

25962 0.031

25963 0.068

25966 0.176

25972 0.024

25973 0.11

25976 0.037

25977 0.035

25978 0.002

25981 0.048

25983 0.064

25984 0.122

25987 0.096

25988 0.059

25989 0.054

25992 0.098

25994 0.074

25996 0.018

25998 0.077

26000 0.034

26003 0.055

26005 0.118

26009 0.07

26010 0.056

26013 0.055

26015 0.121

26018 0.096

26019 0.014

26020 0.069

26024 0.158

26027 0.053

26030 0.079

26031 0.046

26032 0.239

26033 0.02

26034 0.142

26035 0.021

26036 0.131

26037 0.021

26038 0.037

26039 0.045

26040 0.047

26043 0.015

26046 0.076

26047 0.033

26048 0.638

26050 0.02

26051 0.02

26052 0.012

26053 0.031

26054 0.104

26056 0.081

26057 0.015

26058 0.026

26059 0.007

26060 0.008

26061 0.072

26062 0.078

26064 0.087

26065 0.028

26071 0.351

26073 0.028

26074 0.126

26084 0.075

26085 0.243

26086 0.335

26088 0.047

26090 0.042

26091 0.017

26092 0.185

26094 0.113

26097 0.004

26098 0.048

26099 0.018

26100 0.016

26103 0.131

26108 0.084

26112 0.542

26115 0.01

26118 0.038

26119 0.229

26122 0.017

26123 0.167

26127 0.008

26128 0.047

26130 0.012

26133 0.013

26135 0.009

26136 0.044

26137 0.012

26146 0.125

26147 0.066

26148 0.206

26148 0.216

26149 0.548

26150 0.195

26151 0.161

26152 0.514

26153 0.137

26154 0.045

26156 0.302

26160 0.025

26164 0.097

26166 0.154

26167 0.126

26168 0.019

26172 0.213

26173 0.087

26175 0.026

26190 0.007

26191 0.184

26205 0.033

26206 0.23

26207 0.009

26223 0.081

26224 0.015

26227 0.033

26228 0.098

26230 0.077

26231 0.138

26232 0.061

26233 0.114

26234 0.04

26235 0.037

26240 0.083

26249 0.015

26251 0.05

26253 0.21

26254 0.191

26256 0.152

26257 0.131

26258 0.128

26259 0.125

26261 0.056

26262 0.019

26263 0.043

26266 0.06

26267 0.034

26268 0.043

26269 0.024

26270 0.163

26271 0.21

26272 0.063

26273 0.028

26276 0.014

26277 0.226

26278 0.227

26279 0.181

26280 0.028

26281 0.028

26284 0.129

26286 0.106

26287 0.064

26289 0.048

26290 0.358

26291 0.122

26292 0.504

26297 0.131

26298 0.031

26301 0.226

26330 0.112

26353 0.029

26354 0.224

26355 0.594

26468 0.033

26469 0.154

26470 0.02

26471 0.147

26499 0.025

26502 0.1

26503 0.077

26504 0.066

26505 0.107

26507 0.03

26508 0.124

26509 0.033

26512 0.02

26519 0

26520 0.017

26523 0.001

26524 0.082

26525 0.058

26528 0.007

26531 0.152

26538 0.477

26548 0.037

26574 0.108

26575 0.042

26576 0.024

26577 0.07

26585 0.088

26586 0.25

26589 0.112

26608 0.067

26610 0.185

26686 0.077

26707 0.38

26716 0.376

26747 0.17

26750 0.14

26751 0.044

26762 0.467

26872 0.105

26873 0.044

26953 0.02

26960 0.013

26973 0.034

26974 0.606

26984 0.016

26985 0.002

26986 0.002

26993 0.039

26994 0.003

26998 0.237

26999 0.002

27005 0.021

27006 0.088

27010 0.068

27012 0.024

27013 0.088

27019 0.065

27020 0.052

27030 0.175

27031 0.063

27032 0.017

27034 0.057

27035 0.097

27036 0.36

27037 0.103

27039 0.111

27043 0.067

27044 0.013

27063 0.054

27068 0.143

27069 0.045

27071 0.038

27072 0.015

27074 0.329

27076 0.167

27077 0.031

27079 0.128

27085 0.134

27086 0.023

27087 0.009

27090 0.06

27091 0.015

27092 0.009

27094 0.193

27095 0.008

27097 0.042

27101 0.032

27106 0.215

27109 0.133

27111 0.13

27112 0.032

27115 0.044

27120 0.237

27121 0.171

27122 0.09

27123 0.028

27124 0.047

27125 0.053

27125 0.179

27127 0.102

27128 0.043

27129 0.038

27130 0.121

27131 0.008

27132 0.061

27133 0.011

27134 0.101

27136 0.217

27143 0.115

27145 0.039

27146 0.14

27147 0.098

27148 0.088

27152 0.115

27153 0.013

27154 0.041

27156 0.27

27159 0.104

27161 0.003

27163 0.145

27165 0.035

27173 0.034

27175 0.012

27177 0.22

27179 0.341

27183 0.005

27185 0.309

27189 0.153

27190 0.06

27199 0.085

27229 0.009

27231 0.102

27232 0.045

27235 0.086

27236 0.093

27237 0.098

27239 0.031

27240 0.128

27241 0.075

27242 0.066

27243 0.008

27244 0.012

27245 0.091

27247 0.057

27248 0.017

27252 0.002

27253 0.01

27254 0.015

27255 0.056

27257 0.011

27283 0.087

27284 0.159

27285 0.093

27286 0.035

27288 0.12

27290 0.244

27291 0.22

27294 0.153

27295 0.136

27296 0.335

27297 0.077

27300 0.424

27301 0.134

27303 0.04

27304 0.132

27309 0.023

27314 0

27315 0.038

27316 0.008

27319 0.033

27324 0.022

27327 0.027

27328 0.096

27329 0.142

27330 0.07

27332 0.13

27334 0.079

27335 0.009

27336 0.127

27339 0.004

27340 0.081

27342 0.021

27343 0.103

27346 0.121

27347 0.015

27348 0.231

27349 0.149

27352 0.035

27429 0.098

27430 0.03

27433 0.073

27434 0.148

27436 0.061

27439 0.066

27440 0.142

27443 0.108

27445 0.019

28227 0.22

28231 0.151

28232 0.011

28234 0.216

28316 0.024

28378 0.347

28511 0.025

28512 0.03

28513 0.178

28514 0.063

28738 0.449

28952 0.07

28956 0.004

28957 0.167

28959 0.371

28960 0.053

28962 0.116

28964 0.004

28965 0.135

28966 0.015

28968 0.409

28969 0.004

28970 0.07

28971 0.138

28973 0.128

28976 0.082

28977 0.148

28978 0.041

28981 0.037

28982 0.144

28984 0.067

28985 0.003

28986 0.414

28987 0.053

28988 0.082

28991 0.12

28992 0.087

28996 0.013

28998 0.057

28999 0.081

29028 0.075

29035 0.009

29058 0.016

29062 0.054

29063 0.11

29066 0.08

29068 0.019

29070 0.132

29071 0.03

29072 0.026

29074 0.107

29078 0.175

29080 0.168

29081 0.532

29086 0.238

29087 0.108

29088 0.076

29089 0.089

29091 0.005

29093 0.054

29099 0.063

29100 0.031

29101 0.005

29102 0.027

29104 0.071

29105 0.006

29106 0.061

29107 0.006

29108 0.241

29109 0.078

29110 0.034

29113 0.427

29114 0.121

29115 0.023

29116 0.036

29117 0.069

29119 0.036

29121 0.523

29123 0.111

29127 0.087

29128 0.159

29760 0.082

29761 0.034

29763 0.031

29765 0.02

29766 0.06

29767 0.028

29775 0.048

29780 0.039

29781 0.126

29785 0.126

29789 0.002

29798 0.036

29800 0.121

29801 0.045

29803 0.091

29841 0.052

29842 0.046

29843 0.063

29850 0.095

29851 0.189

29855 0.074

29880 0.06

29881 0.15

29882 0.032

29883 0.002

29886 0.06

29887 0.027

29888 0.372

29889 0.081

29890 0.05

29893 0.071

29894 0.068

29896 0.002

29899 0.048

29903 0.082

29906 0.088

29907 0.07

29909 0.059

29911 0.076

29914 0.043

29915 0.05

29916 0.098

29919 0.028

29920 0.043

29922 0.065

29924 0.027

29925 0.011

29926 0.016

29927 0.001

29929 0.092

29930 0.072

29933 0.219

29934 0.024

29937 0.043

29940 0.033

29941 0.427

29942 0.066

29943 0.155

29944 0.168

29945 0.04

29946 0.081

29947 0.26

29948 0.518

29950 0.085

29951 0.068

29953 0.022

29954 0.052

29956 0.039

29957 0.036

29958 0.055

29959 0.009

29960 0.151

29965 0.029

29967 0.039

29968 0.041

29969 0.109

29970 0.033

29974 0.034

29978 0.053

29979 0.051

29982 0.046

29984 0.105

29985 0.104

29986 0.143

29990 0.551

29991 0.641

29992 0.481

29993 0.022

29994 0.053

29995 0.043

29999 0.093

30000 0.003

30008 0.026

30009 0.064

30010 0.006

30011 0.03

30012 0.002

30061 0.05

30062 0.07

30811 0.04

30813 0.144

30814 0.116

30815 0.064

30818 0.047

30819 0.039

30820 0.004

30827 0.019

30832 0.139

30833 0.088

30835 0.359

30836 0.244

30837 0.024

30844 0.017

30845 0.029

30846 0.227

30849 0.029

43847 0.169

43849 0.209

49855 0.069

49860 0.243

50485 0.163

50486 0.155

50487 0.204

50488 0.012

50489 0.217

50506 0.064

50507 0.057

50508 0.118

50509 0.104

50511 0.16

50512 0.143

50515 0.016

50604 0.148

50613 0.152

50614 0.029

50615 0.248

50617 0.085

50618 0.035

50619 0.045

50626 0.019

50636 0.108

50640 0.094

50649 0.136

50650 0.031

50651 0.069

50700 0.104

50717 0.016

50801 0.112

50804 0.042

50805 0.088

50807 0.023

50808 0.039

50809 0.053

50813 0.006

50831 0.225

50835 0.627

50837 0.198

50838 0.286

50839 0.306

50840 0.425

50846 0.017

50848 0.197

50852 0.268

50853 0.177

50855 0.043

50856 0.39

50859 0.052

50861 0.019

50862 0.004

50863 0.01

50937 0.104

50939 0.17

50940 0.108

50943 0.074

50944 0.051

50945 0.166

50964 0.06

50999 0.053

51000 0.12

51004 0.074

51005 2.487

51006 0.027

51008 0.078

51009 0.008

51010 0.07

51012 0.022

51013 0.028

51015 0.054

51016 0.051

51018 0.162

51020 0.063

51022 0.131

51026 0.014

51027 0.068

51028 0.018

51029 0.017

51035 0.044

51046 0.017

51052 0.141

51056 0.055

51057 0.143

51058 0.077

51060 0.049

51061 0.105

51062 0.017

51063 0.042

51065 0

51066 0.116

51067 0.078

51068 0.026

51069 0.093

51070 0.058

51072 0.105

51074 0.033

51076 0.044

51077 0.01

51078 0.078

51079 0.127

51081 0.093

51083 0.192

51084 0.113

51085 0.098

51087 0.044

51088 0.016

51090 0.059

51091 0.066

51092 0.024

51093 0.081

51095 0.039

51096 0.091

51097 1.755

51098 0.046

51099 0.029

51100 0.025

51101 0.073

51103 0.14

51104 0.015

51105 0.038

51106 0.085

51109 0.103

51110 0.098

51111 0.01

51114 0.008

51115 0.09

51118 0.051

51119 0.016

51122 0.077

51124 0.011

51126 0.003

51128 0.006

51129 0.148

51130 0.097

51131 0.302

51132 0.057

51133 0.032

51134 0.206

51135 0.1

51138 0.002

51141 0.016

51142 0.09

51143 0.028

51144 0.117

51146 0.181

51149 0.373

51150 0.08

51151 0.111

51154 0.036

51155 0.074

51156 0.184

51161 0.593

51163 0.11

51164 0.018

51166 0.173

51167 0.108

51168 0.1

51170 0.119

51171 0.11

51172 0.121

51174 0.076

51175 0.049

51176 0.011

51179 0.194

51181 0.092

51182 0.056

51184 0.034

51185 0.023

51186 0.132

51187 0.013

51191 0.649

51193 0.039

51194 0.017

51195 0.019

51196 0.084

51199 0.106

51200 0.089

51201 0.025

51203 0.21

51204 0.124

51206 0.184

51207 0.138

51208 0.09

51209 0.011

51213 0.764

51214 0.556

51218 0.036

51222 0.064

51225 0.09

51226 0.046

51227 0.042

51228 0.035

51230 0.058

51231 0.146

51232 0.06

51234 0.003

51236 0.128

51237 0.22

51246 0.19

51247 0.017

51248 0.014

51249 0.198

51252 0.133

51253 0.112

51256 0.053

51258 0.118

51259 0.028

51263 0.09

51264 0.099

51265 0.111

51267 0.208

51268 0.115

51271 0.052

51274 0.023

51277 0.038

51279 0.16

51280 0.195

51281 0.335

51282 0.299

51283 0.057

51284 0.116

51285 0.037

51286 0.101

51290 0.023

51291 0.105

51292 0.031

51293 0.483

51294 0.123

51296 0.119

51297 0.183

51298 0.174

51300 0.182

51302 0.162

51303 0.054

51304 0.016

51305 0.123

51306 0.055

51307 0.029

51308 0.036

51309 0.096

51310 0.013

51312 0.075

51312 0.836

51313 0.124

51314 0.269

51315 0.14

51316 0.091

51317 0.027

51318 0.147

51319 0.043

51320 0.002

51321 0.241

51322 0.084

51326 0.192

51332 0.232

51333 0.022

51334 0.045

51335 0.178

51337 0.076

51339 0.115

51340 0.029

51341 0.031

51343 0.01

51347 0.005

51351 0.318

51360 0.014

51361 0.039

51362 0.007

51364 0.059

51365 0.11

51366 0.01

51368 0.092

51371 0.024

51377 0.015

51378 0.32

51380 0.061

51384 0.047

51385 0.577

51386 0.015

51388 0.011

51389 0.023

51390 0.21

51393 0.515

51397 0.061

51399 0.077

51400 0.016

51406 0.131

51409 0.134

51421 0.066

51422 0.034

51426 0.138

51428 0.007

51429 0.047

51430 0.082

51433 0.032

51434 0.01

51435 0.05

51438 0.541

51439 0.051

51440 0.002

51442 0.337

51447 0.017

51449 0.134

51451 0.066

51455 0.087

51458 0.145

51460 0.06

51463 0.032

51465 0.04

51466 0.033

51471 0.304

51473 0.19

51474 0.142

51475 0.062

51478 0.141

51479 0.043

51491 0.055

51493 0.003

51495 0.046

51496 0.019

51497 0.014

51503 0.006

51506 0.017

51507 0.262

51512 0.302

51513 0.471

51514 0.055

51517 0.055

51520 0.055

51523 0.025

51526 0.072

51527 0.036

51528 0.017

51529 0.16

51530 0.078

51531 0.65

51533 0.119

51534 0.04

51535 0.098

51537 1.992

51540 0.105

51542 0.039

51547 0.031

51548 0.077

51550 0.148

51552 0.002

51555 0.027

51557 0.118

51560 0.002

51562 0.121

51564 0.058

51566 0.019

51567 0.206

51569 0

51571 0.001

51573 0.071

51574 0.098

51575 0.106

51582 0.022

51585 0.032

51586 0.033

51588 0.054

51592 0.019

51593 0.013

51594 0.09

51599 0.12

51603 0.061

51604 0.04

51606 0.007

51608 0.032

51611 0.057

51614 0.01

51616 0.047

51617 0.016

51619 0.034

51621 0.348

51622 0.033

51626 0.067

51629 0.061

51631 0.006

51633 0.065

51635 0.147

51637 0.018

51642 0.118

51645 0.009

51647 0.017

51652 0.018

51654 0.068

51657 0.221

51659 0.16

51661 0.078

51663 0.011

51665 0.043

51666 0.038

51667 0.073

51668 0.087

51669 0.108

51676 0.048

51678 0.009

51684 0.012

51690 0.016

51691 0

51692 0.016

51696 0.102

51699 0

51700 0.1

51701 0.001

51702 0.075

51703 0.124

51704 0.07

51705 0.386

51706 0.043

51714 0

51716 0.111

51719 0.004

51720 0.134

51725 0.083

51726 0.008

51727 0.047

51728 0.004

51729 0.025

51733 0.085

51734 0.047

51735 0.04

51738 0.086

51741 0.029

51742 0.058

51744 0.41

51751 0.113

51752 0.081

51754 0.019

51755 0.072

51761 0.033

51762 0.007

51765 0.009

51768 0.14

51773 0.105

51776 0.049

51778 0.058

51780 0.019

51802 0.133

51804 0.006

51805 0.179

51806 0.386

51808 0.068

51809 0.023

53335 0.005

53336 0.451

53339 0.018

53340 0.182

53342 0.088

53343 0.043

53347 0.123

53349 0.02

53353 0.047

53354 0.013

53358 0.419

53371 0.02

53373 0.079

53405 0.02

53615 0.029

53616 0.048

53630 0.088

53632 0.274

53635 0.078

53637 0.11

53820 0.399

53822 0.078

53826 0.037

53827 0.42

53828 0.214

53829 0.117

53831 0.09

53832 0.249

53834 0.061

53836 0.05

53838 0.344

53841 0.269

53904 0.271

53905 0.052

53916 0

53938 0.158

53940 0.35

53942 0.054

53944 0.044

53947 0.136

53981 0.012

54014 0.125

54020 0.081

54033 0.184

54039 0.016

54058 0.276

54059 0.132

54069 0.189

54084 0.113

54093 0.13

54097 0.14

54101 0.055

54102 0.34

54103 0.099

54107 0.026

54108 0.096

54148 0.066

54149 0.087

54165 0.005

54187 0.029

54205 0.045

54206 0.094

54207 0.024

54209 0.206

54210 0.484

54212 0.035

54221 0.098

54328 0.003

54329 0.001

54345 0.072

54360 0.182

54361 0.007

54363 0.059

54386 0.103

54414 0.117

54431 0.049

54433 0.027

54434 0.094

54436 0.168

54437 0.04

54438 0.016

54439 0.195

54440 0.027

54442 0.02

54443 0.115

54453 0.04

54454 0.065

54455 0.023

54456 0.106

54457 0.364

54458 1.084

54460 0.051

54461 0.056

54462 0.046

54463 0.035

54464 0.067

54465 0.321

54466 0.199

54467 0.053

54468 0.064

54469 0.045

54470 0.185

54471 0.013

54472 0.039

54474 0.132

54475 0.024

54476 0.088

54477 0.081

54480 0.026

54487 0.025

54490 0.251

54491 0.097

54492 0.469

54496 0.083

54497 0.019

54498 0.019

54499 0

54502 0.012

54503 0.051

54504 0.145

54505 0.036

54507 0.141

54509 0.036

54510 0.038

54511 0.054

54512 0.022

54514 0.056

54516 0.116

54517 0.067

54518 0.12

54520 0.046

54521 0.048

54529 0.123

54530 0.048

54531 0.109

54534 0.146

54535 0.164

54536 0.043

54537 0.24

54538 0.137

54539 0.134

54540 0.078

54541 0.038

54542 0.028

54545 0.058

54546 0.221

54549 0.026

54550 0.12

54551 0.219

54552 0.066

54554 0.093

54555 0.07

54556 0.019

54557 0.018

54558 0.05

54566 0.085

54567 0.077

54576 0.268

54583 0.119

54584 0.122

54586 0.337

54587 0.138

54596 0.442

54602 0.079

54606 0.06

54617 0.016

54620 0.015

54621 0.215

54622 0.014

54623 0.008

54625 0.275

54626 0.098

54627 0.363

54629 0.019

54660 0.162

54661 0.228

54663 0.057

54664 0.019

54665 0.02

54675 0.051

54677 0.088

54680 0.167

54681 0.051

54682 0.289

54700 0.154

54704 0.01

54707 0.024

54708 0.002

54714 0.214

54715 0.007

54716 0.05

54718 0.216

54726 0.08

54733 0.038

54734 0.013

54737 0.136

54738 0.02

54739 0.539

54749 0.121

54751 0.136

54752 0.187

54753 0.068

54754 0.33

54756 0.081

54757 0.083

54758 0.125

54760 0.154

54763 0.09

54764 0.005

54765 0.044

54766 0.171

54768 0.106

54768 0.143

54768 0.161

54769 0.005

54776 0.004

54777 0.315

54778 0.05

54780 0.063

54784 0.717

54785 0.139

54788 0.031

54790 0.269

54793 0.014

54795 0.11

54797 0.007

54798 0.181

54800 0.004

54801 0.217

54802 0.065

54805 0.008

54806 0.133

54808 0.03

54810 0.099

54812 0.12

54813 0.019

54815 0.065

54816 0.133

54819 0.07

54820 0.052

54821 0.169

54822 0.031

54823 0.175

54825 0.216

54827 0.234

54828 0.019

54831 0.061

54832 0.065

54834 0.027

54838 0.074

54839 0.057

54840 0.079

54841 0.065

54842 0.059

54843 0.035

54844 0.282

54845 0.016

54847 0.045

54848 0.074

54849 0.044

54850 0.04

54851 0.064

54852 0.045

54853 0.062

54854 0.167

54855 0.023

54856 0.097

54857 0.097

54858 0.028

54859 0.146

54860 0.395

54862 0.099

54863 0.13

54865 0.306

54866 0.123

54867 0.114

54868 0.063

54869 0.079

54870 0.011

54872 0.123

54873 0.122

54874 0.014

54875 0.131

54878 0.027

54879 0.05

54880 0.06

54881 0.065

54883 0.051

54884 0.117

54885 0.084

54886 0.02

54888 0.087

54890 0.048

54891 0.041

54892 0.137

54894 0.113

54896 1.999

54897 0.052

54898 0.061

54899 0.06

54901 0.053

54903 0.102

54904 0.054

54905 0.142

54908 0.173

54910 0.07

54913 0.073

54914 0.092

54915 0.032

54916 0.096

54918 0.11

54921 0.063

54922 0.029

54925 0.544

54926 0

54927 0.052

54928 0.041

54931 0.139

54933 0.057

54934 0.025

54936 2.562

54937 0.367

54938 0.116

54939 0.048

54940 0.077

54941 0.163

54942 0.149

54943 0.156

54946 0.153

54947 0.064

54948 0.093

54949 0.046

54951 0.141

54952 0.012

54953 0.079

54954 0.038

54955 0.108

54956 0.055

54957 0.014

54959 0.225

54960 0.211

54961 0.111

54962 0.134

54963 0.036

54964 0.302

54965 0.095

54970 0.155

54971 0.021

54972 0.075

54973 0.016

54974 0.104

54976 0.056

54977 0.076

54980 0.112

54981 0.107

54982 0.049

54984 0.172

54986 0.117

54986 0.186

54987 0.102

54988 0.082

54989 0.14

54991 0.22

54993 0.416

54994 0.007

54995 0.096

54996 0.152

54997 0.015

54998 0.226

55001 0.133

55002 0.237

55003 0.169

55005 0.112

55006 0.465

55007 0.049

55008 0.219

55010 0.209

55014 0.06

55015 2.685

55016 0.019

55020 0.07

55022 0.024

55023 0.026

55024 0.217

55026 0.025

55027 0.093

55030 0.153

55032 0.081

55033 0.043

55034 0.152

55035 0.226

55036 0.303

55037 0.155

55038 0.174

55039 0.125

55040 0.069

55041 0.055

55048 0.117

55049 0.185

55051 0.105

55054 0.036

55055 0.116

55062 0.03

55063 0.241

55064 0.175

55065 0.109

55066 0.045

55069 0.034

55070 0.006

55071 0.245

55074 0.105

55075 0.117

55076 0.241

55079 0.02

55080 0.204

55081 0.048

55082 0.005

55084 0.025

55086 0.128

55088 0.121

55090 0.1

55092 0.074

55093 0.086

55094 0.083

55095 0.012

55100 0.055

55102 0.052

55103 0.013

55105 0.081

55107 0.048

55108 0.092

55109 0.132

55111 0.077

55112 0.153

55113 0.21

55114 0.081

55116 0.014

55117 0.043

55118 0.031

55119 0.039

55120 0.127

55124 0.066

55125 0.267

55127 0.08

55128 0.23

55129 0.048

55130 0.024

55131 0.109

55132 0.038

55133 0.138

55135 0.073

55137 0.014

55139 0.124

55140 0.024

55142 0.178

55143 0.131

55144 0.022

55146 0.162

55148 0.075

55150 0.289

55151 0.166

55154 0.121

55156 0.01

55157 0.083

55159 0.217

55160 0.046

55161 0.01

55163 0.049

55164 0.167

55165 0.152

55166 0.214

55167 0.006

55168 0.118

55170 0.045

55173 0.117

55174 0.025

55175 0.018

55176 0.001

55177 0.08

55179 0.148

55181 0.024

55182 0.012

55183 0.18

55184 0.108

55187 0.039

55188 0.022

55190 0.027

55191 0.08

55192 0.058

55193 0.008

55195 0.228

55197 0.011

55198 0.041

55203 0.011

55204 0.026

55205 0.035

55206 0.016

55207 0

55208 0.069

55210 0.052

55211 0.388

55212 0.047

55213 0.023

55214 0.049

55215 0.119

55216 0.107

55218 0.109

55220 0.027

55222 0.059

55223 0.005

55224 0.061

55225 0.117

55226 0.025

55227 0.042

55229 0.05

55230 0.123

55231 0.279

55234 0.001

55236 0.06

55237 0.159

55238 0.027

55239 0.106

55240 0.077

55243 0.018

55244 0.136

55245 0.077

55246 0.021

55247 0.161

55248 0.053

55249 0.175

55250 0.124

55253 0.111

55254 0.034

55257 0.02

55258 0.095

55259 0.226

55260 0.09

55265 0.161

55266 0.073

55268 0.048

55269 0.011

55270 0.066

55273 0.079

55274 0.016

55276 0.047

55277 0.12

55278 0.074

55280 0.058

55281 0.242

55282 0.126

55283 0.047

55284 0.003

55285 0.065

55286 0.276

55288 0.014

55289 0.122

55291 0.021

55296 0.017

55297 0.127

55298 0.025

55299 0.049

55300 0.086

55301 0.282

55303 0.396

55304 0.272

55311 0.077

55312 0.041

55314 0.078

55315 0.178

55316 0.089

55317 0.084

55320 0.292

55321 0.072

55322 0.059

55323 0.073

55324 0.02

55325 0.076

55326 0.109

55327 0

55328 0.17

55329 0.142

55331 0.061

55332 0.026

55334 0.034

55335 0.134

55337 0.026

55339 0.018

55341 0.099

55342 0.006

55343 0.076

55344 0.194

55347 0.13

55349 0.104

55350 0.111

55352 0.103

55353 0.15

55355 0.428

55357 0.107

55359 0.124

55361 0.023

55363 0.356

55364 0.104

55365 0.361

55366 0.045

55367 0.114

55374 0.098

55379 0.025

55380 0.464

55388 0.138

55421 0.034

55422 0.392

55423 0.339

55425 0.043

55432 0.049

55435 0.028

55437 0.053

55450 0.109

55454 0.045

55466 0.223

55471 0.086

55486 0.05

55500 0.061

55501 0.088

55502 0.085

55503 0.057

55504 0.191

55507 0.112

55508 0.141

55509 0.082

55510 0.124

55512 0.05

55515 0.02

55520 0.043

55521 0.041

55526 0.067

55527 0.055

55530 0.04

55531 0.026

55532 0.052

55536 0.119

55540 0.155

55544 0.033

55553 0.018

55554 0.147

55561 0.088

55565 0.016

55567 0.078

55568 0.037

55571 0.02

55572 0.126

55573 0.157

55576 0.132

55577 0.046

55578 0.066

55582 0.126

55584 0.054

55585 0.008

55586 0.062

55588 0.04

55589 0.034

55591 0.06

55592 0.303

55593 0.008

55596 0.11

55600 0.144

55601 0.165

55603 0.02

55604 0.044

55604 0.098

55605 0.031

55607 0.03

55608 0.122

55609 0.215

55610 0.021

55611 0.004

55612 0.063

55613 0.33

55614 0.099

55616 0.11

55617 0.026

55619 0.042

55620 0.211

55621 0.098

55622 0.094

55623 0.086

55624 0.031

55625 0.024

55626 0.023

55627 0.068

55629 0.092

55630 0.185

55631 0.109

55632 0.124

55633 0.006

55635 0.072

55636 0.022

55638 0.082

55640 0.069

55643 0.007

55647 0.119

55651 0.061

55652 0.162

55654 0.006

55656 0.023

55657 0.141

55658 0.045

55659 0.382

55660 0.036

55661 0.039

55662 0.018

55663 0.316

55665 0.298

55666 0.016

55667 0.084

55668 0.076

55669 0.049

55670 0.151

55676 0.047

55677 0.063

55679 0.048

55680 0.019

55681 0.039

55683 0.037

55684 0.125

55686 0.083

55687 0.064

55689 0.071

55690 0.013

55691 0.019

55693 0.197

55696 0.001

55697 0.03

55698 2.473

55699 0.094

55700 0.078

55701 0.081

55702 0.108

55703 0.004

55704 0.024

55705 0.006

55706 0.068

55707 0.051

55709 0.005

55711 0.074

55713 0.217

55714 0.007

55714 0.02

55715 0.008

55716 0.042

55717 0.031

55718 0.045

55720 0.081

55721 0.324

55722 0.231

55723 0.049

55726 0.03

55727 0.113

55728 0.189

55731 0.017

55733 0.096

55734 0.071

55735 0.019

55737 0.003

55738 0.071

55740 0.045

55742 0.012

55743 0.106

55745 0.087

55746 0.102

55748 0.049

55749 0.027

55750 0.044

55751 0.137

55752 0.006

55753 0.037

55754 0.063

55755 0.18

55756 0.015

55757 0.161

55758 0.01

55759 0.035

55760 0.067

55761 0.028

55763 0.016

55764 0.046

55765 0.084

55766 0.022

55770 0.036

55771 0.162

55773 0.025

55775 0.114

55777 0.024

55779 0.181

55780 0.175

55781 0.115

55783 0.126

55784 0.054

55785 0.149

55786 0.456

55787 0.054

55788 0.042

55789 0.037

55790 0.011

55791 0.194

55793 0.084

55794 0.126

55795 0.022

55796 0.104

55798 0.118

55799 0.011

55800 0.013

55802 0.059

55806 0.126

55808 0.253

55809 0.049

55810 0.043

55811 0.141

55813 0.083

55814 0.298

55815 0.141

55818 0.042

55819 0.01

55821 0.119

55823 0.013

55824 0.117

55825 0.196

55827 0.066

55831 0.005

55832 0.004

55833 0.101

55836 0.096

55837 0.392

55840 0.107

55841 0.337

55843 0.075

55844 0.018

55846 0.058

55847 0.078

55848 0.093

55849 0.11

55850 0.072

55852 0.053

55854 0.053

55856 0.101

55857 0.208

55858 0.03

55859 0.132

55860 0.024

55861 0.012

55862 0.106

55863 0.313

55869 0.02

55870 0.044

55871 0.093

55872 0.068

55879 0.136

55884 0.019

55885 0.003

55888 0.211

55889 0.409

55892 0.044

55897 0.222

55898 0.03

55900 0.623

55902 0.035

55904 0.082

55905 0.057

55906 0

55907 0.036

55909 0.03

55911 0.384

55914 0.054

55915 0.059

55916 0.16

55917 0.056

55920 0.02

55922 0.027

55924 0.141

55929 0.011

55930 0.062

55954 0.036

55958 0.006

55959 0.03

55966 0.149

55967 0.225

55968 0.018

55971 0.061

55973 0.115

55975 0.014

55997 0.369

55998 0.322

55999 0.318

56000 0.365

56001 0.351

56005 0.069

56006 0.015

56034 0.08

56052 0.123

56061 0.023

56062 0.065

56063 0.103

56121 0.145

56122 0.127

56123 0.156

56124 0.242

56125 0.235

56126 0.253

56127 0.254

56128 0.236

56129 0.254

56130 0.217

56131 0.117

56132 0.122

56133 0.138

56147 0.165

56154 0.358

56156 0.503

56158 0.092

56159 0.313

56164 0.122

56165 0.185

56169 0.404

56171 0.05

56172 0.009

56180 0.014

56181 0.029

56203 0.114

56204 0.173

56241 0.158

56243 0.081

56244 0.211

56245 0.181

56246 0.211

56252 0.752

56253 0.174

56254 0.013

56255 0.11

56256 0.094

56257 0.041

56259 0.026

56261 0.038

56262 0.338

56265 0.084

56267 0.082

56269 0.059

56271 0.243

56287 0.237

56288 0.046

56300 0.296

56301 0.04

56302 0.117

56311 0.297

56341 0.001

56342 0.151

56344 0.199

56413 0.089

56474 0.061

56475 0.013

56478 0.051

56479 0.02

56521 0.149

56548 0.077

56603 0.022

56605 0.053

56606 0.082

56624 0.111

56648 0

56649 0.148

56650 0.052

56655 0.021

56658 0.013

56659 0.087

56660 0.006

56666 0.041

56667 0.531

56672 0.172

56673 0.266

56674 0.031

56675 0.039

56681 0.011

56683 0.039

56729 0.365

56731 0.345

56751 0.011

56829 0.424

56833 0.162

56834 0.062

56848 0.105

56849 0.13

56850 0.036

56852 0.139

56853 0.003

56884 0.052

56886 0.045

56888 0.014

56889 0.004

56890 0.178

56893 0.036

56894 0.079

56895 0.087

56896 0.011

56897 0.021

56898 0.053

56899 0.058

56901 0.028

56902 0.042

56905 0.127

56907 0.04

56910 0.045

56912 0.037

56913 0.058

56914 0.084

56915 0.088

56916 0.037

56919 0.036

56920 0.08

56922 0.091

56923 0.144

56924 0.04

56926 0.041

56927 0.038

56929 0.002

56930 0.817

56931 0.105

56934 0

56937 0.045

56938 0.291

56940 0.034

56941 0.182

56942 0.105

56943 0.009

56945 0.126

56946 0.021

56947 0.026

56950 0.039

56951 0.266

56953 0.073

56954 0.058

56955 0.337

56956 0.003

56957 0.045

56961 0.132

56963 0.428

56964 0.249

56965 0

56970 0.003

56977 0.027

56979 0.173

56980 0.024

56981 0.054

56983 0.05

56984 0.082

56985 0.113

56986 0.163

56987 0.066

56992 0.075

56993 0.027

56994 0.068

56995 0.031

56996 0.036

56997 0.07

56998 0.011

56999 0.047

57001 0.102

57002 0.181

57003 0.011

57007 0.042

57010 0.058

57016 0.117

57017 0.04

57018 0.014

57019 0.104

57020 0.033

57030 0.01

57035 0.148

57037 0.029

57038 0.085

57045 0.013

57047 0.133

57053 0.046

57055 0.141

57057 0.002

57062 0.123

57082 0.134

57084 0.014

57085 0.128

57088 0.109

57089 0.042

57091 0.243

57092 1.765

57094 0.088

57095 0.014

57096 0.214

57097 0.053

57099 0.151

57101 0.043

57102 0.032

57103 0.172

57105 0.194

57107 0.067

57110 0.092

57113 0.01

57115 0.162

57117 0.049

57118 0.011

57119 0.255

57121 0.134

57122 0.048

57124 0.12

57125 0.114

57126 0.914

57127 0.106

57128 0.056

57129 0.137

57130 0.031

57134 0.059

57135 0.141

57136 0.062

57140 0.024

57142 0.128

57144 0.031

57146 0.155

57148 0.015

57149 0.064

57152 0.2

57153 0.055

57154 0.008

57156 0.1

57157 0.035

57158 0.007

57159 0.024

57161 0.026

57162 0.007

57165 0.083

57167 0.149

57172 0.045

57175 0.031

57176 0.1

57178 0.01

57179 0.107

57180 0.007

57180 0.041

57182 0.046

57184 0.097

57185 0.076

57186 0.071

57187 0.01

57188 0.247

57191 0.559

57192 0.36

57194 0.108

57198 0.017

57205 0.095

57210 0.122

57211 0.099

57213 0.01

57214 0.052

57216 0.006

57217 0.057

57219 0.223

57221 0.036

57222 0.007

57223 0.018

57224 0.139

57228 0.098

57231 0.014

57234 0.047

57282 0.012

57325 0.026

57326 0.191

57332 0.035

57333 0.039

57335 0.101

57336 0.483

57337 0.052

57338 0.041

57343 0.46

57369 0.009

57379 0.045

57380 0.097

57381 0.04

57393 0.08

57396 0.018

57403 0.012

57405 0.141

57406 0.041

57410 3.149

57415 0.178

57418 0.101

57419 0.011

57446 0.02

57448 0.023

57452 0.021

57453 0.018

57455 0.13

57456 0.06

57458 0.035

57459 0.007

57460 0.021

57462 0.046

57464 0.023

57465 0.059

57467 0.053

57468 0.009

57470 0.08

57471 0.245

57472 0.021

57473 0.064

57474 0.391

57475 0.091

57476 0.015

57477 0.129

57478 0.023

57479 0.052

57480 0.111

57482 0.218

57484 0.029

57485 0.042

57486 0.052

57488 0.039

57489 0.188

57492 0.031

57493 0.182

57494 0.014

57495 0.024

57496 0.036

57497 0.028

57498 0.032

57501 0.318

57504 0.029

57506 0.355

57507 0.036

57508 0.02

57509 0.079

57510 0.069

57511 0.039

57512 0.072

57513 0.061

57514 0.125

57515 0.022

57519 0.302

57520 0.025

57521 0.019

57522 0.017

57523 1.466

57529 0.188

57530 0.078

57532 0.026

57533 0.052

57534 0.005

57535 0.052

57536 0.169

57537 0.061

57539 0.041

57540 0.059

57541 0.057

57542 0.047

57545 0.07

57546 0.084

57547 0.416

57549 0.072

57551 0.005

57552 0.092

57554 0.024

57555 0.009

57556 0.025

57558 0.044

57559 0.057

57561 0.492

57562 0.249

57563 0.037

57565 0.005

57567 0.019

57568 0.041

57569 0.122

57571 0.164

57572 0.038

57573 0.312

57574 0.057

57575 0.015

57576 0.116

57577 0.175

57578 0.012

57580 0.047

57582 0.192

57584 0.079

57586 0.06

57587 0.164

57589 0.04

57590 0.014

57591 0.083

57592 0.074

57593 0.028

57594 0.132

57595 0.04

57596 0.07

57597 0.095

57599 0.006

57600 0.143

57602 0.175

57604 0.184

57605 0.051

57606 0.028

57609 0.008

57611 0.057

57613 0.074

57614 0.04

57615 0.375

57616 0.029

57617 0.023

57619 0.166

57621 0.03

57622 0.012

57623 0.076

57624 0.05

57626 0.024

57628 0.057

57630 0.051

57631 0.031

57634 0.075

57636 0.019

57639 0.121

57642 0.166

57643 0.019

57644 0.021

57645 0.047

57646 0.05

57647 0.089

57648 0.114

57649 0.048

57650 0.071

57653 0.193

57654 0.176

57655 0.03

57657 0.044

57658 0.064

57659 0.067

57661 0.194

57663 0.433

57664 0.228

57666 0.15

57669 0.096

57671 0.016

57673 0.035

57674 0.179

57677 0.445

57678 0.046

57679 0.045

57680 0.027

57683 0.325

57684 0.438

57685 0.028

57687 0.009

57688 0.011

57689 0.003

57690 0.038

57692 0.253

57693 0.096

57695 0.051

57696 0.055

57697 0.288

57699 0.01

57700 0.044

57701 0.19

57703 0.063

57704 0.072

57705 0.165

57706 0.022

57708 1.787

57709 0.018

57713 0.108

57714 0.426

57715 0.049

57716 0.111

57718 0.05

57719 0.066

57720 0.651

57721 0.008

57722 0.068

57724 0.089

57727 0.037

57728 0.059

57729 0.143

57730 0.452

57731 0.054

57732 0.097

57758 0.058

57761 0.176

57763 0.041

57787 0.272

57794 0.058

57795 0.03

57798 0.038

57801 0.334

57805 0.048

57817 0.393

57819 0

57820 0.062

57821 0.154

57822 0.049

57823 0.379

57826 0

57827 0.167

57828 0.384

57834 0.19

57863 0.025

57864 0.133

58155 0.003

58157 0.033

58158 0.051

58190 0.007

58191 0.39

58472 0.073

58473 0.037

58475 0.343

58476 0.079

58477 0.05

58478 0.037

58480 0.042

58484 0.161

58488 0.112

58489 0.019

58490 0.005

58491 0.369

58492 0.505

58493 0.018

58494 0.117

58495 0.064

58497 0.091

58498 0.039

58499 0.035

58500 0.391

58504 0.138

58505 0.003

58506 0.065

58508 0.084

58509 0.313

58511 0.244

58512 0.013

58513 0.443

58515 0.042

58516 0.028

58517 0.004

58524 0.054

58525 0.04

58526 0.052

58527 0.041

58529 0.043

58533 0.003

58985 0.174

59067 0.243

59084 0.099

59269 0.108

59271 0.084

59272 0.105

59274 0.01

59277 0.053

59283 0.293

59284 0.216

59307 0.119

59335 0.006

59336 0.086

59338 0.049

59339 0.042

59340 0.216

59341 0.026

59342 0.105

59343 0.066

59344 0.073

59345 0.019

59349 0.005

59350 0.086

59352 0.054

59353 0.015

60312 0.047

60314 0.073

60370 1.348

60385 0.254

60386 0.144

60401 0.108

60412 0.022

60436 0.035

60437 0.211

60468 0.446

60481 0.035

60482 0.041

60484 0.047

60485 0.028

60487 0.05

60488 0.162

60490 0.065

60492 0.103

60494 0.13

60495 0.037

60496 0.044

60506 0.1

60509 0.068

60528 0.104

60529 0.038

60558 0.057

60559 0.005

60560 0.08

60561 0.073

60592 0.071

60598 0.131

60625 0.027

60626 0.071

60672 0.287

60673 0.011

60675 0.06

60676 0.128

60678 0.068

60680 0.026

60681 0.061

60682 0.034

60684 0.054

60685 0.023

63027 0.025

63035 0.05

63036 0.196

63826 0.055

63827 0.099

63874 0.021

63876 0.005

63877 0.115

63891 0.034

63892 0.109

63893 0.035

63894 0.027

63895 0.048

63897 0.096

63898 0.142

63899 0.104

63901 0.336

63904 0.229

63905 0.059

63908 0

63910 0.133

63916 0.007

63917 0.091

63923 0.106

63925 0.068

63928 0.101

63929 0.048

63931 0.077

63932 0.003

63933 0.179

63935 0.026

63939 0.233

63941 0.075

63943 0.163

63946 0.112

63947 0.495

63948 0.304

63951 0.196

63967 0.133

63971 0.042

63974 0.009

63976 0.065

63978 0.199

63979 0.137

63982 0.035

64005 0.065

64061 0.151

64062 0.015

64063 0.152

64065 0.072

64066 0.141

64067 0.027

64072 0.031

64073 0.295

64078 0.129

64080 0.085

64081 0.272

64083 0.008

64084 0.031

64087 0.079

64089 0.088

64090 0.214

64091 0.11

64092 0.1

64093 0.017

64094 0.064

64096 0.144

64097 0.043

64098 0.129

64102 0.019

64105 0.146

64106 0.09

64108 0.374

64111 0.285

64115 0.143

64116 0.061

64121 0.005

64122 0.075

64123 0.137

64127 0.133

64129 0.065

64130 0.013

64131 0.043

64132 0.048

64135 0.123

64137 0.02

64147 0.063

64149 0.053

64151 0.092

64167 0.348

64168 0.016

64172 0.093

64173 0.159

64174 0.2

64175 0.069

64180 0.181

64184 0.314

64207 2.026

64210 0.059

64211 0.007

64215 0.09

64216 0.344

64218 0.103

64219 0.469

64220 0.167

64221 0.082

64222 0.213

64224 0.057

64225 0.049

64232 0.219

64240 0.119

64241 0.116

64283 0.113

64284 0.165

64285 0.026

64318 0.055

64319 0.021

64321 0.079

64324 0.018

64326 0.012

64327 0.02

64328 0.01

64332 0.105

64333 0.107

64342 0.243

64343 0.097

64344 0.547

64375 0.018

64388 0.028

64393 0.065

64395 0.05

64396 0.089

64397 0.128

64398 0.02

64399 0.056

64400 0.02

64403 0.148

64405 0.027

64407 0.082

64409 0.302

64410 0.191

64411 0.066

64412 0.103

64418 0.026

64419 0.053

64420 0.16

64421 0.231

64423 0.174

64425 0.13

64426 0.012

64428 0.073

64429 0.04

64430 0.15

64431 0.01

64446 0.07

64478 0.557

64499 0.156

64499 0.179

64518 0.089

64577 0.056

64579 0.022

64581 0.272

64582 0.064

64599 0.041

64600 0.164

64641 0.003

64641 0.005

64645 0.003

64651 0.156

64682 0.042

64689 0.095

64708 0.011

64710 0.022

64743 0.005

64744 0.021

64745 0.103

64746 0.058

64747 0.056

64748 0.023

64750 0.005

64753 0.16

64754 0.032

64755 0.104

64756 0.048

64760 0.093

64761 0.109

64762 0.049

64763 0.052

64766 0.166

64768 0.053

64769 0.049

64770 0.216

64771 0.171

64772 0.131

64773 0.093

64776 0.129

64777 0.011

64778 0.036

64779 0.087

64780 0.111

64781 0.103

64782 0.164

64783 2.308

64784 0.068

64785 0.055

64786 0.047

64787 0.081

64788 0.124

64789 0.095

64792 0.063

64793 0.083

64794 0.153

64795 0.001

64798 0.018

64799 0.168

64800 0.211

64801 0.105

64802 0.106

64805 0.069

64806 0.11

64816 0.215

64837 0.036

64838 0.038

64840 0.014

64841 0.009

64843 0.007

64844 0.071

64848 0.014

64849 0.07

64850 0.095

64853 0.013

64854 0

64855 0.055

64856 0.138

64858 0.123

64859 0.058

64860 0.261

64863 0.109

64864 0.04

64866 0.097

64895 0.029

64901 0.035

64902 0.087

64919 0.028

64921 0.03

64924 0.026

64925 0.211

64926 0.096

64927 0.165

64943 0.029

64946 0.23

64949 0.184

64951 0.1

64960 0.221

64963 0.185

64965 0.138

64968 0.084

64969 0.123

64978 0.079

64979 0.259

64981 0.156

64983 0.162

65005 0.093

65008 0.12

65009 0.073

65012 0.145

65018 0.131

65055 0.01

65056 0.022

65057 0.263

65059 0.062

65061 0.042

65065 0.048

65078 0.068

65080 0.085

65082 0.015

65083 0.085

65084 0.046

65095 0.114

65109 0.068

65110 0.192

65117 0.011

65123 0.008

65125 0.606

65217 0.096

65220 0.044

65244 0.096

65249 0.039

65250 0.281

65251 0.461

65258 0.123

65260 0.059

65264 0.009

65265 0.238

65266 0.069

65267 0.034

65268 0.116

65975 0.18

65977 0.044

65981 0.082

65982 0.344

65983 0.078

65985 0.058

65986 0.055

65987 0.3

65988 0.361

65989 0.039

65991 0.039

65992 0.097

65997 0.042

65999 0.075

66000 0.155

66002 0.165

66004 0.366

66005 0.054

66008 0.068

66036 0.024

66037 0.034

78986 0.016

78987 0.071

78988 0.114

78989 0.051

78990 0.027

78991 0.035

78992 0.118

78994 0.142

78995 0.229

79000 0.269

79001 0.094

79003 0.12

79004 0.073

79006 0.114

79007 0.255

79009 0.021

79012 0.015

79014 0.263

79016 0.016

79017 0.091

79018 0.004

79019 0.104

79020 0.045

79023 0.049

79025 0.1

79026 0.017

79027 0.099

79029 0.13

79031 0.056

79034 0.02

79035 0.033

79038 0.084

79039 0.082

79047 0.021

79048 0.667

79050 0.139

79053 0.067

79054 0.035

79056 0.154

79057 0.031

79058 0.113

79065 0.012

79066 0.061

79068 0.069

79070 0.033

79071 0.018

79072 0.224

79073 0.162

79075 0.08

79077 0.157

79080 0.129

79081 0.099

79083 0.253

79084 0.059

79085 0.035

79087 0.133

79088 0.359

79092 0.142

79094 0.071

79096 0.034

79098 0.25

79102 0.071

79109 0.014

79132 0.138

79133 0.055

79134 0.022

79135 0.104

79137 0.061

79139 0.012

79142 0.01

79144 0.209

79148 0.085

79149 0.763

79152 0.1

79153 0.134

79155 0.133

79156 0.062

79157 0.038

79158 0.062

79161 0.012

79169 0.06

79170 0.054

79172 0.211

79174 0.145

79175 0.333

79178 0.162

79180 0.032

79183 0.03

79184 0.017

79187 0.043

79190 0.138

79191 0.04

79230 0.46

79258 0.125

79269 0.019

79290 0.095

79315 0.278

79364 0.095

79365 0.08

79366 0.356

79368 0.721

79369 0.15

79370 0.3

79411 0.109

79414 0.021

79415 0.064

79442 0.09

79443 0.142

79444 0.204

79465 0.703

79567 0.118

79568 0.149

79571 0.035

79572 0.049

79573 0.03

79574 0.169

79575 0.062

79576 0.053

79577 1.622

79581 0.105

79582 0.149

79585 0.087

79586 0.029

79587 0.123

79589 0.164

79590 0.066

79591 0.008

79594 0.059

79595 0.039

79598 0.14

79602 0.224

79603 0.203

79605 0.029

79608 0.074

79609 0.063

79611 0.111

79612 0.052

79613 0.099

79616 0.056

79618 0.005

79622 0.08

79623 0.101

79625 0.067

79626 0.037

79627 0.118

79628 0.111

79629 1.177

79631 0.057

79632 0.066

79633 0.053

79634 0.114

79635 0.539

79637 0.091

79640 0.452

79642 0.053

79643 0.038

79644 0.123

79645 0.047

79646 0.004

79647 0.045

79648 0.305

79649 0.546

79650 0.107

79651 0.046

79654 0.006

79657 0.122

79658 0.063

79659 0.036

79659 0.043

79659 1.045

79660 0.06

79661 0.158

79663 0.181

79665 0.03

79666 0.014

79668 0.021

79669 0.215

79670 0.08

79671 0.057

79673 0.131

79674 0.086

79675 0.222

79677 0.047

79679 0.083

79682 0.298

79683 0.027

79684 0.018

79685 0.013

79689 0.151

79691 0.071

79693 0.079

79694 0.088

79695 0.086

79696 0.7

79698 0.028

79699 0.012

79701 0.101

79705 0.051

79706 0.046

79707 0.278

79709 0.041

79710 0.118

79711 0.106

79712 0.133

79713 0.333

79714 0.087

79716 0.034

79718 0.007

79719 0.091

79720 0.094

79722 0.083

79723 0.043

79724 0.05

79727 0.021

79728 0.182

79729 0.132

79730 0.414

79731 0.086

79733 0.07

79734 0.072

79738 0.223

79739 0.036

79740 0.279

79742 0.126

79744 0.447

79745 0.07

79746 0.071

79748 0.203

79750 0.336

79751 0.023

79752 0.095

79753 0.082

79754 0.008

79759 0.045

79762 0.242

79763 0.148

79767 0.046

79770 0.125

79772 0.032

79776 0.041

79777 0.172

79778 0.28

79781 0.162

79782 0.2

79783 0.09

79784 0.108

79785 0.074

79786 0.078

79788 0.272

79789 0.183

79791 0.056

79791 0.548

79792 0.33

79794 0.047

79796 0.03

79798 0.073

79799 0.318

79800 0.154

79801 0.132

79803 0.108

79805 0.015

79807 0.104

79809 0.077

79810 0.169

79811 0.045

79812 0.215

79814 0.093

79815 0.074

79816 0.41

79817 0.009

79819 0.164

79820 0.329

79822 0.085

79823 0.098

79825 0.209

79827 0.041

79829 0.03

79831 0.134

79832 0.126

79833 0.069

79834 0.079

79836 0.082

79837 0.023

79841 0.048

79842 0.093

79843 0.34

79845 0.022

79846 0.144

79847 0.053

79848 0.127

79849 0.113

79852 0.116

79853 0.146

79856 0.092

79857 0.47

79858 0.147

79861 0.101

79863 0.267

79864 0.215

79865 0.326

79866 0.169

79867 0.208

79868 0.21

79869 0.021

79870 0.126

79871 0.197

79872 0.014

79875 0.043

79877 0.033

79879 0.056

79882 0.056

79883 0.142

79884 0.28

79886 0.068

79887 0.137

79888 0.073

79890 0.125

79892 0.177

79893 0.019

79894 0.142

79895 0.083

79896 0.101

79898 0.462

79899 0.046

79901 0.165

79903 0.014

79906 0.195

79912 0.09

79913 0.06

79918 0.106

79919 0.154

79923 0.264

79924 0.24

79925 0.136

79927 0.763

79929 0.142

79930 0.228

79931 0.243

79932 0.093

79933 0.09

79934 0.076

79937 0.151

79943 0.5

79944 0.132

79947 0.043

79948 0.069

79949 0.257

79953 0.036

79954 0.025

79955 0.134

79956 0.081

79957 0.225

79958 0.192

79959 0.011

79960 0.035

79961 0.044

79962 0.097

79964 0.067

79968 0.175

79971 0.051

79973 0.531

79974 0.31

79977 0.035

79982 0.022

79983 0.059

79984 0.234

79987 0.11

79989 0.013

79990 0.038

79993 0.062

79998 0.342

80000 0.088

80003 0.106

80004 0.038

80006 0.022

80007 0.177

80008 0.337

80011 0.037

80012 0.03

80013 0.021

80014 0.097

80017 0.165

80018 0.043

80019 0.009

80020 0.096

80021 0.099

80022 0.201

80023 0.31

80024 0.116

80025 0.007

80028 0.051

80031 0.027

80032 0.641

80034 0.032

80036 0.008

80045 0.068

80055 0.072

80059 0.03

80063 0.217

80067 0.046

80070 0.188

80071 0.266

80086 0.114

80095 0.054

80097 0.212

80108 0.083

80114 0.043

80115 0.039

80117 0.146

80119 0.071

80119 0.102

80122 0.03

80124 0.024

80125 0.19

80127 0.15

80128 0.029

80131 0.064

80133 0.32

80142 0.095

80144 0.059

80145 0.022

80146 0.01

80148 0.06

80149 0.108

80150 0.132

80152 0.272

80153 0.024

80155 0.01

80157 0.101

80162 0.159

80167 0.013

80168 0.117

80169 0.209

80176 0.012

80177 0.118

80178 0.25

80179 0.139

80184 0.075

80185 0.17

80194 0.024

80196 0.06

80198 0.115

80199 0.109

80201 0.048

80204 0.002

80205 0.047

80206 0.056

80208 0.054

80209 0.111

80210 0.045

80216 0.182

80218 0.009

80219 0.083

80221 0.136

80222 0.09

80223 0.17

80224 0.058

80228 0.022

80230 0.028

80231 0.09

80232 0.002

80233 0.168

80235 0.128

80237 0.131

80243 0.026

80254 0.105

80255 0.037

80256 0.015

80258 3.453

80262 0.016

80263 0.083

80264 0.394

80267 0.029

80270 0.08

80271 0.146

80273 0.065

80274 0.046

80298 0.151

80304 0.242

80305 0.032

80306 0.026

80308 0.061

80310 0.079

80311 0.075

80312 0.309

80313 0.382

80314 0.043

80315 0.009

80316 0.344

80317 0.178

80318 0.061

80320 0.02

80321 0.123

80323 0.178

80324 0.09

80325 0.038

80326 0.025

80329 0.645

80331 0.009

80332 0.181

80333 0.002

80335 0.323

80339 0.218

80341 0.215

80342 0.133

80344 0.038

80346 0.067

80347 0.069

80351 0.014

80352 0.109

80380 0.22

80381 0.036

80700 0.122

80704 0.216

80705 0.025

80709 0.275

80714 0.123

80723 0.041

80724 0.125

80725 0.035

80726 0.476

80728 0.062

80731 0.079

80737 0.117

80740 0.083

80741 0.149

80745 0.237

80746 0.203

80757 0.014

80758 0.017

80759 0.446

80760 0.139

80761 0.3

80762 0.015

80764 0.034

80765 0.092

80774 0.09

80777 0.158

80781 0.133

80789 0.027

80790 0.008

80816 0.069

80817 0.216

80818 0.031

80820 0.058

80821 0.046

80824 0.049

80830 0.494

80833 0.456

80851 0.02

80853 0.092

80854 0.022

80862 0.285

80863 0.071

80895 0.029

80975 0.131

81025 0.504

81027 0.049

81029 0.129

81030 0.401

81031 0.156

81033 0.053

81034 0.054

81035 0.041

81037 0.041

81285 0.033

81490 0.046

81491 0.043

81492 0.411

81494 0.561

81501 0.179

81502 0.017

81532 0.038

81533 0.057

81537 0.117

81539 0.038

81542 0.14

81543 0.125

81544 0.053

81545 0.033

81550 0.09

81551 0.012

81552 0.307

81553 0.003

81554 0.06

81555 0.041

81558 0.066

81559 0.056

81562 0.051

81563 0.02

81565 2.282

81566 0.042

81567 0.064

81570 0.068

81572 0.049

81573 0.042

81577 0.023

81579 0.044

81602 0.112

81603 0.014

81606 0.323

81607 0.045

81609 0.008

81610 0.184

81611 0.025

81614 0.023

81616 0.445

81618 0.509

81619 0.012

81620 0.157

81622 0.341

81623 0.453

81624 0.088

81626 0.067

81627 0.065

81628 0.077

81631 0.028

81669 0.035

81671 0.03

81688 0

81689 0.016

81691 0.179

81693 0.226

81696 0.11

81704 0.04

81706 0.038

81786 0.05

81788 0.074

81792 0.115

81794 0.032

81796 0.088

81831 0.014

81832 0.021

81833 0.195

81839 0.023

81844 0.105

81847 0.035

81848 0.058

81849 0.052

81850 0.145

81855 0.034

81856 0.511

81857 0.065

81858 0.269

81870 0.273

81872 0.081

81873 0.019

81875 0.155

81876 0.007

81887 0.179

81890 0.164

81892 0.185

81894 0.005

81926 0.04

81928 0.037

81929 0.017

81930 0.147

81931 0.483

81932 0.146

83259 0.086

83394 0.03

83401 0.183

83416 0.515

83417 0.501

83439 0.023

83440 0.022

83445 0.161

83446 0.108

83447 0.068

83448 0.18

83449 0.196

83450 0.123

83451 0.201

83452 0.028

83461 0.162

83464 0.115

83468 0.038

83473 0.082

83475 0.096

83478 0.043

83479 0.116

83482 0.039

83483 0.273

83538 0.133

83539 0.134

83540 0.166

83543 0.02

83544 0.038

83546 0.338

83547 0.154

83548 0.023

83549 0.04

83550 0.204

83551 0.237

83590 0.055

83591 0.081

83593 0.089

83594 0.072

83595 0.06

83596 0.119

83597 0.269

83604 0.008

83606 0.024

83607 0.008

83636 0.085

83637 0.048

83639 0.341

83641 0.091

83642 0.115

83650 0.123

83657 0.028

83658 0.014

83659 0.114

83660 0.008

83661 0.277

83666 0.273

83667 0.043

83690 0.029

83692 0.167

83693 0.074

83694 0.138

83695 0.206

83696 0.046

83697 0.114

83698 0.002

83699 0.033

83700 0.089

83706 0.034

83707 0.101

83714 0.167

83715 0.091

83716 0.143

83719 0

83723 0.027

83732 0.126

83733 0.07

83734 0.107

83737 0.023

83740 0.432

83741 0.005

83742 0.056

83743 0.045

83744 0.454

83746 0.025

83752 0.025

83755 0.175

83759 0.017

83786 0.07

83795 0.098

83844 0.538

83850 0.072

83851 0.121

83852 0.196

83853 0.142

83854 0.146

83855 0.056

83856 0.056

83857 0.066

83858 0.096

83860 0.092

83861 0.217

83862 0.033

83869 0.312

83871 0.03

83872 0.074

83873 0.022

83874 0.047

83875 0.114

83876 0.185

83878 0.318

83879 0.089

83881 0.206

83882 0.269

83884 0.119

83886 0.114

83889 0.281

83890 0.127

83891 0.07

83892 0.008

83893 0.129

83894 0.164

83895 0.175

83896 0.091

83897 0.098

83902 0.123

83931 0.02

83932 0.197

83933 0.154

83937 0.087

83940 0.07

83941 0.091

83942 0.086

83943 0.05

83953 0.405

83954 0.805

83955 0.207

83957 0.101

83959 0.082

83982 0.351

83983 0.193

83988 0

83989 0.038

83990 0.16

83992 0.085

83998 0.213

83999 0.036

84000 0.084

84033 0.124

84034 0.162

84054 0.182

84056 0.027

84057 0.06

84058 0.063

84059 0.53

84060 0.185

84062 0.16

84063 0.211

84064 0.066

84065 0.148

84066 0.327

84067 0.036

84070 0.282

84071 0.193

84072 0.13

84073 0.193

84074 0.31

84075 0.353

84076 0.115

84079 0.104

84081 0.157

84085 0.06

84100 0.179

84101 0.173

84102 0.054

84103 0.361

84105 0.079

84108 0.053

84109 0.103

84124 0.246

84125 0.338

84128 0.086

84131 0.114

84132 0.194

84133 0.076

84134 0.012

84135 0.059

84138 0.153

84140 0.328

84141 0.069

84142 0.222

84144 0.153

84146 0.084

84148 0.016

84152 0.069

84154 0.039

84159 0.04

84162 0.017

84162 0.02

84163 0.121

84164 0.067

84166 0.265

84168 0.022

84171 0.077

84172 0.055

84173 0.135

84174 0.114

84176 0.387

84179 0.184

84181 0.061

84182 0.198

84186 0.248

84188 0.01

84189 0.062

84190 0.155

84191 0.029

84193 0.048

84196 0.031

84203 0.406

84206 0.02

84210 0.446

84216 0.022

84217 0.105

84220 0.069

84220 0.536

84221 0.05

84223 0.219

84225 0.098

84229 0.084

84230 0.017

84231 0.021

84232 0.032

84233 0.19

84236 0.119

84239 0.076

84240 0.094

84243 0.022

84245 0.085

84246 0.004

84248 0.041

84249 0.065

84251 0.054

84253 0.035

84254 0.035

84255 0.069

84256 0.15

84260 0.143

84261 0.137

84262 0.04

84263 0.074

84264 0.1

84265 0.023

84266 0.112

84267 0.103

84268 0.176

84269 0.061

84270 0.049

84271 0.045

84272 0.013

84273 0.136

84276 0.067

84277 0.193

84279 0.013

84280 0.014

84283 0.22

84285 0.043

84286 0.13

84287 0.006

84289 0.029

84292 0.048

84293 0.072

84295 0.013

84298 0.088

84299 0.055

84300 0.054

84301 0.044

84302 0.009

84303 0.208

84304 0.11

84305 0.053

84306 0.179

84307 0.227

84310 0.242

84313 0.008

84314 0.65

84315 0.03

84319 0.093

84324 0.082

84326 0.067

84328 0.034

84331 0.081

84332 0.293

84333 0.018

84336 0.01

84337 0.011

84340 0.096

84342 0.068

84343 0.107

84364 0.038

84365 0.305

84376 0.35

84417 0.095

84419 0.179

84432 0.091

84433 0.044

84435 0.105

84437 0.027

84439 0.099

84440 0.039

84441 0.096

84443 0.06

84444 0.089

84445 0.039

84446 0.007

84447 0.024

84448 0.048

84450 0.069

84451 0.199

84456 0.057

84458 0.01

84460 0.245

84462 0.125

84464 0.307

84466 0.03

84467 0.237

84498 0.116

84501 0.087

84503 0.561

84514 0.17

84515 0.056

84516 0.004

84517 0.13

84518 0.015

84519 0.149

84522 0.031

84524 0.137

84529 0.026

84530 0.101

84532 0.068

84540 0.008

84541 0.012

84542 0.063

84545 0.091

84549 0.06

84552 0.123

84553 0.011

84570 0.041

84612 0.024

84614 0.026

84616 0.203

84618 0.026

84620 0.157

84623 0.011

84626 0.27

84627 0.354

84628 0.028

84629 0.027

84629 0.139

84630 0.06

84631 0.012

84632 0.1

84634 0.099

84636 0.059

84639 0.101

84640 0.059

84643 0.13

84645 0.115

84647 0.059

84649 0.031

84650 0.159

84654 0.412

84656 0.008

84660 0.13

84661 0.01

84662 0.036

84665 0.07

84666 0.287

84668 0.132

84669 0.024

84671 0.267

84674 0.301

84675 0.076

84676 0.03

84678 0.028

84679 0.024

84681 0.079

84684 0.164

84687 0.001

84688 0.136

84690 0.197

84691 0.814

84694 0.117

84695 0.043

84696 0.117

84698 0.156

84699 0.189

84700 0.171

84701 0.16

84705 0.122

84706 0.037

84708 0.067

84709 0.403

84717 0.094

84720 0.064

84722 0.211

84725 0.042

84727 0.051

84733 0.031

84734 0.071

84735 0.128

84749 0.053

84750 0.175

84752 0.092

84769 0.144

84775 0.098

84787 0.11

84792 0.441

84795 0.091

84798 0.466

84800 0.044

84803 0.028

84804 0.291

84807 0.103

84811 0.104

84812 0.159

84814 0.03

84818 0.184

84820 0.057

84824 0.177

84826 0.148

84830 0.239

84836 0.068

84844 0

84851 0.325

84859 0.044

84861 0.029

84864 0.154

84866 0.049

84867 0.059

84868 0.244

84869 0.066

84870 0.059

84872 0.014

84873 0.207

84874 0.546

84876 0.056

84878 0.284

84879 0.087

84881 0.139

84883 0.056

84888 0.12

84889 0.116

84891 0.141

84893 0.055

84894 0.006

84896 0

84898 0.052

84899 0.058

84902 0.217

84904 0.085

84905 0.049

84909 0.226

84910 0.073

84912 0.05

84914 0.608

84915 0.053

84916 0.053

84918 0.171

84919 0.08

84920 0.084

84922 0.08

84923 0.107

84924 0.333

84925 0.061

84926 0.003

84928 0.026

84929 0.056

84930 0.148

84932 0.03

84934 0.221

84936 0.153

84937 0.004

84938 0.055

84940 0.017

84944 0.052

84945 0.02

84947 0.06

84948 0.077

84950 0.009

84952 0.106

84953 0.224

84955 0.072

84957 0.087

84958 0.097

84959 0.017

84961 0.005

84962 0.033

84966 0.033

84967 0.048

84968 0.419

84969 0.034

84970 0.164

84971 0.08

84975 0.098

84984 0.101

84985 0.063

84991 0.004

84993 0.013

85002 0.206

85004 0.011

85007 0.071

85012 0.143

85013 0.067

85014 0.196

85015 0.135

85016 0.096

85019 0.101

85021 0.027

85025 0.007

85027 0.426

85285 0.176

85290 0.373

85291 0.178

85293 0.086

85300 0.051

85301 0.117

85313 0.018

85315 0.034

85319 0.135

85329 0.137

85352 0.205

85358 0.017

85358 0.153

85359 0.104

85360 0.077

85363 0.34

85365 0.127

85366 0.138

85369 0.007

85377 0.134

85378 0.067

85379 0.121

85395 0.157

85397 0.011

85403 0.014

85407 0.068

85409 0.186

85413 0.337

85414 0.052

85415 0.084

85416 0.057

85417 0.559

85437 0.028

85438 0.264

85439 0.061

85440 0.012

85441 0.197

85442 0.149

85443 0.154

85444 0.122

85445 0.077

85446 0.062

85449 0.262

85451 0.024

85452 0.245

85455 0.045

85455 0.188

85456 0.195

85457 0.078

85458 0.048

85459 0.282

85460 0.185

85461 0.056

85462 0.179

85463 0.048

85464 0.095

85465 0.047

85476 0.055

85477 0.056

85478 0.115

85479 0.067

85480 0.537

85481 0.226

85508 0.026

85509 0.235

85569 0.205

85865 0.116

86614 0.257

87178 0.051

87769 0.229

88455 0.053

88745 0.085

89766 0.249

89777 0.17

89778 0.226

89780 0.022

89782 1.229

89790 0.296

89792 0.032

89795 0.022

89796 0.019

89797 0.014

89801 0.098

89845 0.093

89846 0.157

89848 0.049

89849 0.087

89853 0.019

89857 0.033

89858 0.501

89866 0.165

89869 0.153

89872 0.117

89874 0.115

89876 0.149

89882 0.194

89883 0.487

89884 0.007

89885 0.378

89886 0.267

89887 0.238

89890 0.107

89894 0.169

89910 0.068

89927 0.024

89932 2.969

89944 0.128

89953 0.033

89958 0.107

89970 0.019

89978 0.067

90007 0.036

90019 0.253

90025 0.132

90050 0.138

90075 0.446

90110 0.447

90113 0.085

90134 0.035

90139 0.053

90141 0.137

90161 0.029

90167 0.085

90187 0.087

90199 0.342

90204 0.102

90231 0.046

90249 0.014

90268 0.052

90288 0.273

90293 0.023

90316 0.552

90321 0.53

90324 0.096

90326 0.129

90342 0.168

90353 0.088

90355 0.026

90378 0.054

90379 0.053

90381 0.201

90390 0.023

90407 0.071

90410 0.074

90411 0.106

90416 0.077

90417 0.138

90427 0.069

90441 0.098

90459 0.088

90462 0.253

90480 0.176

90488 0.129

90506 0.194

90522 0.109

90523 0.152

90525 0.02

90527 0.119

90529 0.167

90550 0.022

90576 0.388

90592 0.353

90624 0.094

90627 0.056

90634 0.094

90637 0.355

90639 0.116

90655 0.707

90665 0.034

90668 3.376

90678 0.067

90693 0.067

90701 0.01

90780 0.016

90799 0.173

90806 1.684

90809 0.021

90826 0.074

90827 0.462

90835 0.186

90843 0.163

90850 0.109

90853 0.366

90864 0.078

90865 0.326

90871 0.247

90952 0.232

90956 1.636

90957 0.149

90990 0.133

90993 0.042

91010 0.016

91012 0.138

91039 0.063

91057 0.157

91074 0.424

91107 0.065

91120 0.451

91147 0.08

91179 0.072

91181 0.136

91252 0.051

91272 0.073

91289 0.11

91298 0.086

91304 0.038

91319 0.055

91373 0.099

91392 0.342

91404 0.011

91408 0.006

91409 0.198

91419 0.129

91431 0.12

91442 0.101

91445 0.008

91452 0.098

91522 0.064

91523 0.309

91526 0.076

91531 0.125

91543 0.104

91544 0.12

91574 0.135

91582 0.431

91584 0.045

91608 0.007

91614 0.071

91624 0.1

91646 0.119

91647 0.073

91653 0.079

91683 0.032

91687 0.074

91689 0.115

91694 0.015

91695 0.154

91703 0.197

91734 0.252

91746 0.011

91749 0.145

91750 0.045

91752 0.242

91754 0.024

91768 0.033

91775 0.054

91782 0.029

91807 0.197

91828 0.165

91833 0.01

91851 0.044

91860 0.161

91862 0.179

91869 0.075

91875 0.058

91893 0.178

91894 0.254

91937 0.347

91942 0.109

91947 0.037

91949 0.04

91977 0.155

91978 0.088

92014 0.078

92086 0.132

92092 0.138

92105 0.025

92126 0.048

92140 0.043

92154 0.021

92196 0.105

92211 0.069

92235 0.093

92241 0.253

92255 0.053

92270 0.168

92283 0.514

92291 0.274

92304 0.327

92305 0.072

92312 0.024

92344 0.162

92345 0.164

92359 0.425

92369 0.015

92370 0.067

92379 0.11

92399 0.12

92400 0.005

92421 0.079

92454 0.114

92482 0.188

92521 0.604

92558 0.06

92565 0.062

92591 0.084

92595 0.265

92597 0.002

92610 0.12

92667 0.186

92675 0.027

92689 0.128

92691 0.064

92703 0.037

92736 0.064

92737 0.05

92745 0.075

92747 0.296

92749 0.159

92797 0.232

92799 0.038

92822 0.133

92840 0.122

92856 0.012

92906 0.021

92912 0.022

92922 0.037

92935 0.077

92949 0.029

92979 0.109

92999 0.114

93010 0.086

93035 0.105

93082 0.187

93099 0.338

93100 0.101

93107 0.111

93109 0.218

93129 0.062

93134 0.47

93145 0.015

93164 0.109

93166 0.101

93185 0.074

93190 0.16

93210 0.089

93233 0.2

93273 0.312

93323 0.385

93343 0.144

93349 0.202

93377 0.145

93380 0.023

93408 0.006

93426 0.18

93432 0.316

93436 0.097

93487 0.101

93492 0.225

93517 0.133

93550 0.162

93587 0.13

93589 0.335

93594 0.096

93611 0.024

93621 0.092

93624 0.011

93627 0.024

93649 0.086

93661 0.049

93663 0.092

93664 0.021

93973 0.015

93974 0.189

93978 0.207

93979 0.102

94005 0.045

94015 0.104

94025 0.295

94025 0.574

94030 0.017

94031 0.05

94032 0.006

94033 0.14

94039 0.524

94056 0.077

94059 0.294

94081 0.034

94097 0.014

94101 0.006

94104 0.023

94120 0.103

94121 0.052

94122 0.146

94134 0.047

94137 0.15

94160 0.088

94234 0.12

94239 0

94240 0.247

95681 0.077

96764 0.153

103910 2.207

112398 0.046

112399 0.014

112401 0.128

112487 0.094

112495 0.165

112574 0.019

112609 0.071

112611 0.042

112616 0.099

112714 0.005

112724 0.085

112744 0.284

112752 0.223

112755 0

112770 0.124

112812 0.063

112817 0.057

112840 0.113

112849 0.069

112869 0.01

112885 0.063

112936 0.006

112939 0.056

112942 0.047

112950 0.011

112970 0.147

113026 0.066

113115 0.241

113130 0.188

113174 0.116

113177 0.115

113178 0.094

113189 0.039

113201 0.056

113220 0.125

113230 0.328

113251 0.081

113263 0.021

113277 0.223

113278 0.172

113419 0.002

113451 0.086

113457 0.025

113510 0.141

113612 0.144

113622 0.09

113675 0.119

113730 0.085

113746 0.033

113791 0.119

113802 0.205

113828 0.12

113829 0.08

113835 0.401

113878 0.085

114026 0.494

114034 0.07

114049 0.126

114088 0.012

114112 0.063

114134 0.06

114294 0.074

114327 0.086

114548 0.109

114569 0.042

114571 0.33

114609 0.143

114758 0.313

114771 0.153

114780 0.162

114781 0.022

114782 0.298

114783 0.017

114784 0.024

114786 0.021

114788 0.013

114789 0.013

114790 0.158

114791 0.066

114792 0.011

114793 0.036

114795 0.062

114798 0.012

114799 0.003

114800 0.04

114801 0.055

114803 0.159

114804 0.063

114818 0.01

114822 0.165

114823 0.038

114826 0.177

114836 0.452

114876 0.047

114879 0.083

114880 0.011

114881 0.038

114882 0.021

114883 0.014

114884 0.053

114885 0.043

114897 0.131

114898 0.031

114899 0.014

114900 0.028

114904 0.231

114905 0.026

114907 0.016

114926 0.08

114928 0.745

114932 0.159

114960 0.268

114987 0.1

114991 0.03

115004 0.298

115019 0.06

115098 0.057

115106 0.093

115111 0.07

115123 0.043

115196 0.614

115201 0.039

115207 0.004

115265 0.036

115273 0.136

115290 0.086

115350 0.146

115352 0.461

115353 0.071

115361 0.216

115362 0.23

115399 0.198

115426 0.05

115509 0.041

115548 0.029

115572 0.046

115584 0.084

115650 0.293

115677 0.097

115701 0.3

115703 0.057

115704 0.014

115708 0.103

115727 0.083

115752 0.044

115761 0.084

115795 0.041

115817 0.106

115825 0.01

115827 0.013

115861 0.125

115908 0.033

115948 0.192

115950 0.041

115992 0.006

116039 0.012

116064 0.04

116068 0.121

116071 0.274

116085 0.198

116092 0.019

116113 0.038

116115 0.072

116123 0.09

116135 0.002

116138 0.018

116150 0.049

116151 0.14

116154 0.082

116159 0.11

116173 0.075

116179 0.139

116225 0

116236 0.068

116254 0.16

116255 0.183

116328 0.136

116337 0.066

116362 0.054

116369 0.262

116372 0.206

116379 0.196

116412 0.61

116442 0.002

116443 0.035

116444 0.137

116447 0.171

116448 0.047

116449 0.254

116461 0.036

116496 0.169

116511 0.704

116512 0.32

116534 0.13

116540 0.079

116541 0.222

116729 0.028

116835 0.035

116840 0.087

116843 0.171

116844 0.298

116931 0.017

116966 0.063

116969 0.116

116983 0.035

116984 0.095

116985 0.044

116987 0.013

116988 0.016

117143 0.022

117144 0.391

117153 0.276

117154 0.036

117155 0.235

117156 0.125

117157 0.233

117166 0.076

117177 0.064

117194 0.354

117195 0.398

117245 0.2

117246 0.099

117247 0.091

117248 0.141

117283 0.135

117285 0.483

117289 0.297

117531 0.021

117532 0.093

117583 0.03

117608 0.178

118426 0.022

118427 0.007

118429 0.098

118432 0.183

118433 0.122

118442 0.227

118471 0.333

118490 0.132

118491 0.117

118611 0.168

118663 0.235

118738 0.29

118788 0.09

118812 0.012

118813 0.067

118856 0.125

118881 0.084

118924 0.079

118932 0.076

118980 0.056

118987 0.07

119016 0.445

119391 0.101

119392 0.311

119395 0.085

119467 0.169

119587 0.059

120065 0.177

120066 0.282

120071 0.225

120103 0.069

120114 0.021

120224 0.121

120329 0.262

120376 0.223

120406 0.257

120425 0.327

120534 0.04

120892 0.08

120935 0.197

121053 0.164

121227 0.082

121256 0.096

121260 0.089

121268 0.061

121274 0.105

121278 0.047

121340 0.025

121355 0.046

121391 0.158

121441 0.089

121457 0.142

121504 0.005

121506 0.23

121536 0.009

121549 0.164

121551 0.008

121599 0.195

121601 0.008

121642 0.099

121643 0.113

121793 0.45

121951 0.327

121951 0.593

121952 0.027

122011 0.044

122042 0.112

122046 0.258

122060 0.036

122402 0.073

122416 0.091

122481 0.073

122509 0.187

122525 0.02

122553 0.012

122616 0.321

122618 0.163

122622 0.018

122664 0.091

122704 0.083

122706 0.114

122769 0.196

122773 0.051

122786 0.039

122809 0.069

122830 0.058

122876 0.073

122945 0.272

122961 0.093

122970 0.172

123016 0.024

123036 0.067

123041 0.031

123096 0.069

123099 0.087

123169 0.029

123263 0.11

123283 0.058

123355 0.065

123591 0.1

123624 0.194

123775 0.342

123811 0.132

123872 0.25

123876 0.146

123879 0.012

123920 0.066

123970 0.393

124044 0.04

124045 0.295

124093 0.274

124152 0.168

124220 0.863

124221 0.243

124245 0.061

124274 0.02

124359 0.048

124401 0.105

124402 0.89

124404 0.075

124446 0.1

124454 0.089

124460 0.143

124535 0.068

124540 0.003

124565 0.155

124583 0.072

124590 0.02

124599 0.303

124626 0.159

124739 0.122

124773 0.171

124783 0.511

124790 0.133

124817 0.112

124842 0.036

124857 0.039

124872 0.179

124912 0.136

124923 0.083

124925 0.055

124930 0.019

124961 0.075

124975 0.188

124976 0.04

124989 0.411

125058 0.066

125061 0.176

125115 0.181

125206 0.066

125228 0.137

125336 0.056

125476 0.035

125488 0.032

125919 0.528

125931 0.566

125950 0.072

125972 0.419

125981 0.131

125988 0.065

126003 0.008

126017 0.393

126068 0.359

126069 0.496

126070 0.485

126074 0.243

126075 0.208

126119 0.034

126123 0.308

126129 0.101

126133 0.109

126206 0.314

126231 0.447

126248 0.3

126272 0.38

126282 0.119

126295 0.486

126298 0.104

126299 0.03

126306 0.299

126308 0.04

126321 0.097

126326 0.07

126328 0.252

126353 0.236

126374 0.023

126402 0.131

126410 0.081

126432 0.193

126433 0.127

126520 0.071

126549 0.345

126567 0.102

126626 0.087

126637 0.348

126638 0.312

126661 0.196

126731 0.165

126789 0.13

126820 0.277

126823 0.13

126859 0.297

126868 0.149

126917 0.015

126961 0.003

126969 0.112

127124 0.088

127247 0.068

127253 0.177

127254 0.094

127255 0.1

127281 0.119

127294 0.098

127343 0.029

127396 0.51

127428 0.052

127435 0.062

127544 0.003

127557 0.619

127579 0.204

127665 0.194

127700 0.099

127707 0.117

127731 0.139

127733 0.185

127795 0.231

127829 0.003

127833 0.011

127845 0.094

127933 0.003

127943 0.096

128025 0.053

128061 0.22

128077 0.014

128102 0.252

128153 0.16

128209 0.416

128229 0.25

128239 0.089

128272 0.086

128344 0.204

128346 0.304

128387 0.081

128414 0.104

128434 0.101

128486 0.067

128497 0.111

128553 0.052

128602 0.205

128637 0.038

128653 0.092

128674 0.053

128817 0.179

128822 0.606

128859 0.175

128869 0.018

128876 0.2

128954 0.251

128989 0.08

129049 0.036

129138 0.05

129285 0.045

129303 0.042

129401 0.033

129446 0.276

129450 0.065

129521 0.221

129530 0.199

129531 0.098

129563 0.053

129607 0.18

129642 0.077

129684 0.096

129787 0.131

129804 0.086

129831 0.051

129852 0.25

129868 0.788

129880 0.018

130013 0.109

130026 0.117

130029 0.038

130074 0.006

130120 0.246

130132 0.121

130162 0.186

130271 0.083

130340 0.023

130355 0.089

130367 0.055

130399 0.015

130497 0.013

130507 0.01

130535 0.111

130557 0.025

130560 0.316

130574 0.029

130576 0.131

130589 0.069

130612 0.014

130617 0.049

130733 0.031

130749 0.638

130752 0.212

130814 0.091

130827 0.062

130940 0.178

130951 0.19

131034 0.004

131118 0.01

131177 0.18

131368 0.048

131375 0.204

131377 0.044

131408 0.036

131450 0.417

131474 0.17

131544 0.108

131566 0.084

131578 0.109

131616 0.093

131669 0.048

131831 0.266

131873 0.929

131920 0.231

132001 0.169

132014 0.187

132112 0.058

132158 0.095

132160 0.049

132200 0.187

132204 0.022

132228 0.168

132243 0.412

132299 0.085

132320 0.144

132321 0.109

132604 0.016

132612 0.067

132625 0.355

132660 0.021

132671 0.166

132720 0.102

132789 0.015

132851 0.178

132864 0.015

132884 0.187

132949 0.178

132954 0.071

133015 0.066

133121 0.075

133308 0.114

133396 0.28

133418 0.231

133482 0.41

133491 0.381

133522 0.12

133558 0.112

133584 0.071

133619 0.073

133686 0.024

133688 0.33

133690 0.028

133746 0.056

133923 0.203

133957 0.104

134121 0.182

134145 0.084

134147 0.106

134218 0.078

134265 0.063

134266 0.079

134285 0.242

134288 0.114

134353 0.067

134391 0.132

134429 0.081

134430 0.069

134466 0.432

134492 0.003

134510 0.014

134526 0.107

134548 0.056

134549 0.26

134553 0.042

134829 0.012

134860 0.051

134864 0.156

134957 0.012

135112 0.104

135114 0.227

135138 0.042

135152 0.051

135228 0.172

135293 0.134

135295 0.083

135398 0.516

135644 0.223

135886 0.316

135892 0.069

135927 0.458

136051 0.231

136227 0.07

136259 0.499

136263 0.233

136288 0.146

136306 0.047

136319 0.004

136332 0.172

136371 0.097

136541 0.233

136853 0.062

136895 0.163

136991 0.083

137075 0.195

137362 0.163

137392 0.067

137492 0.087

137695 0.025

137735 0.119

137797 0.204

137835 0.216

137868 0.022

137872 0.077

137886 0.082

137902 0.308

137964 0.015

137970 0.017

137994 0.101

138009 0.194

138046 0.022

138151 0.595

138199 0.017

138307 0.202

138429 0.189

138474 0.047

138639 0.116

138649 0.561

138724 0.253

139065 0.015

139081 0.552

139170 0.168

139189 0.14

139201 0.045

139212 0.283

139221 0.212

139231 0.012

139285 0.15

139322 0.135

139341 0.018

139378 0.089

139411 0.011

139420 0.346

139422 0.468

139425 0.228

139562 0.269

139599 0.091

139628 0.278

139716 0.13

139760 0.111

139818 0.02

139886 0.024

140032 0.054

140258 0.23

140432 0.174

140453 0.667

140456 0.058

140458 0.036

140460 0.005

140461 0.019

140462 0.17

140465 0.043

140469 0.064

140545 0.135

140578 0.044

140612 0.156

140628 0.132

140679 0.006

140680 0.375

140683 0.575

140685 0.05

140686 0.214

140689 0.014

140690 0.269

140691 0.138

140699 0.238

140700 0.039

140701 0.081

140706 0.08

140707 0.09

140710 0.04

140711 0.067

140730 0.004

140732 0.118

140738 0.168

140766 0.116

140767 0.046

140801 0.033

140803 0.13

140807 0.095

140823 0

140825 0.053

140831 0.11

140836 0.065

140838 0.028

140856 0.156

140862 0.013

140870 0.265

140873 0.472

140876 0.146

140880 0.311

140881 0.398

140885 0.228

140886 0.023

140890 0.053

140893 0.24

140894 0.235

140901 0.023

142679 0.087

142680 0.135

142684 0.099

142685 0.063

142686 0.055

142689 0.07

142891 0.022

142913 0.178

142940 0.095

143098 0.01

143153 0.19

143187 0.013

143241 0.185

143282 0.286

143379 0.21

143384 0.052

143425 0.015

143458 0.023

143471 0.034

143503 0.035

143570 0.182

143630 0.366

143684 0.015

143686 0.031

143689 0.178

143872 0.046

143879 0.05

143884 0.152

143888 0.08

143903 0.102

144100 0.062

144108 0.035

144110 0.056

144124 0.047

144132 0.203

144165 0.037

144193 0.059

144195 0.125

144203 0.297

144233 0.128

144347 0.162

144402 0.008

144406 0.11

144423 0.207

144453 0.026

144455 0.112

144577 0.031

144608 0.335

144717 0.179

144809 0.189

144811 0.139

144983 0.018

145173 0.07

145226 0.08

145258 0.021

145264 0.285

145270 0.047

145376 0.176

145389 0.159

145407 0.288

145447 0.166

145483 0.152

145497 0.171

145501 0.413

145508 0.13

145567 0.015

145581 0.017

145645 0.119

145741 0.347

145748 0.206

145773 0.038

145781 0.059

145788 0.219

145814 0.241

145864 0.118

145873 0.199

145942 0.211

145957 0.136

146057 1.684

146059 0.083

146174 0.054

146177 0.159

146198 0.09

146206 0.082

146212 0.074

146223 0.022

146225 0.485

146227 0.118

146279 0.142

146310 0.206

146330 0.02

146395 0.036

146433 0.189

146434 0.235

146439 0.112

146456 0.136

146542 0.205

146547 0.14

146556 0.258

146562 0.344

146664 0.05

146691 1.089

146705 0.243

146722 0.437

146754 0.021

146754 0.03

146760 0.065

146771 0.196

146779 0.192

146845 0.045

146849 0.043

146850 0.101

146852 0.346

146853 0.261

146857 0.265

146862 0.026

146894 0.274

146923 0.058

146956 0.203

147007 0.062

147011 0.352

147015 0.092

147111 0.052

147138 0.129

147179 0.019

147183 0.051

147199 0.169

147323 0.158

147339 0.024

147341 0.007

147372 0.056

147381 0.033

147409 0.099

147463 0.024

147495 0.055

147645 0.17

147657 0.34

147685 0.377

147686 0.58

147687 0.687

147694 0.676

147700 0.307

147719 0.301

147744 0.245

147746 0.081

147807 0.112

147841 0.168

147872 0.185

147945 0.385

147948 0.428

147949 0.447

147965 0.185

147968 0.083

147991 0.076

148022 0.303

148109 0.444

148113 0.13

148137 0.254

148213 0.341

148229 0.218

148252 0.036

148254 0.416

148268 0.358

148281 0.016

148327 0.179

148362 0.045

148423 0.11

148479 0.036

148523 0.188

148581 0.319

148641 0.048

148646 0.157

148713 0.246

148738 0.061

148741 0.104

148753 0.078

148789 0.069

148808 0.065

148811 0.171

148867 0.025

148870 0.364

148979 0.099

149041 0.036

149069 0.2

149076 0.009

149095 0.195

149175 0.026

149233 0.192

149345 0.04

149371 0.036

149420 0.003

149428 0.084

149466 0.114

149469 0.015

149473 0.231

149499 0.159

149563 0.412

149603 0.022

149620 0.19

149628 0.429

149643 0.299

149647 0.347

149685 0.608

149699 0.187

149708 0.24

149840 0.302

149951 0.067

149954 0.083

149986 0.048

149998 0.252

150082 0.23

150084 0.368

150094 0.111

150159 0.655

150160 0.257

150244 0.258

150248 0.25

150275 0.13

150280 0.134

150350 0.303

150365 0.112

150368 0.129

150372 0.417

150379 0.212

150383 0.569

150465 0.03

150468 0.168

150572 0.032

150684 0.077

150696 0.171

150709 0.168

150726 0.018

150737 0.045

150763 0.105

150864 0.036

150946 0.064

150962 0.076

151011 0.075

151050 0.108

151112 0.209

151126 0.048

151176 0.102

151188 0.099

151194 0.08

151195 0.103

151242 0.075

151246 0.327

151254 0.291

151258 0.222

151306 0.104

151313 0.097

151393 0.111

151531 0.07

151556 0.059

151613 0.067

151636 0.305

151647 0.034

151648 0.313

151649 0.375

151651 0.144

151658 0.603

151742 0.003

151790 0.127

151835 0.006

151871 0.485

151887 0.092

151888 0.344

151903 0.048

151963 0.011

152002 0.039

152006 0.009

152015 0.083

152024 0.12

152028 0.136

152100 0.054

152137 0.089

152185 0.134

152189 0.043

152206 0.148

152273 0.654

152330 0.028

152404 0.069

152405 0.535

152485 0.033

152503 0.121

152518 0.053

152559 0.011

152579 0.096

152687 0.398

152789 0.027

152815 0.091

152831 0.128

152926 0.057

152940 0.293

153020 0.015

153090 0.014

153129 0.049

153201 0.11

153222 0.031

153241 0.045

153328 0.135

153364 0.016

153396 0.029

153443 0.246

153527 0

153561 0.24

153572 0.04

153579 0.206

153642 0.149

153643 0.122

153657 0.177

153733 0.109

153743 0.115

153745 0.266

153768 0.056

153769 0.11

153830 0.04

154007 0.071

154043 0.043

154075 0.106

154091 0.096

154141 0.09

154150 0.57

154197 0.083

154214 0.015

154467 0.111

154661 0.03

154664 0.23

154743 0.017

154791 0.031

154796 0.057

154807 0.023

154810 0.082

154865 0.22

155006 0.281

155036 0.676

155038 0.364

155054 0.805

155060 0.187

155066 0.103

155184 0.153

155185 0.176

155368 0.18

155382 0.023

155400 0.173

155465 0.059

157285 0.127

157310 0.353

157313 0.351

157378 0.021

157506 0.006

157570 0.121

157574 0.087

157657 0.125

157680 0.054

157697 0.293

157724 0.203

157739 0.154

157753 0.136

157769 0.029

157773 0.281

157777 0.093

157807 0.019

157855 0.213

157869 0.066

157922 0.115

158038 0.013

158056 0.187

158062 0.222

158067 0.147

158135 0.127

158158 0.106

158160 0.14

158177 0.435

158219 0.07

158234 0.131

158248 0.332

158297 0.235

158326 0.131

158358 0.156

158376 0.271

158381 0.141

158399 0.217

158401 0.301

158405 0.014

158431 0.386

158471 0.075

158506 0.311

158521 0.401

158747 0.052

158763 0.103

158787 0.245

158798 0.402

158809 0.605

158835 0.1

158866 0.01

158931 0.201

158983 0.368

159013 0.083

159090 0.096

159091 0.512

159163 0.255

159296 0.052

159686 0.101

159963 0.095

159989 0.13

160140 0.204

160287 0.181

160298 0.076

160313 0.214

160335 0.025

160364 0.401

160419 0.105

160428 0.047

160492 0.257

160518 0.033

160728 0.077

160760 0.006

160762 0.172

160777 0.144

160851 0.043

160857 0.185

160897 0.079

161003 0.062

161142 0.08

161145 0.023

161176 0.143

161198 0.218

161247 0.025

161253 0.047

161291 0.057

161357 0.007

161394 0.402

161436 0.027

161497 0.071

161502 0.228

161514 0.066

161582 0.128

161635 0.058

161742 0.033

161753 0.171

161823 0.113

161829 0.179

161835 0.27

161882 0.139

161931 0.137

162137 0.414

162239 0.082

162282 0.033

162333 0.253

162387 0.21

162394 0.293

162417 0.073

162427 0.045

162461 0.452

162466 0.042

162494 0.024

162514 0.039

162515 0.092

162517 0.094

162605 0.102

162963 0.524

162966 0.468

162998 0.299

163033 0.054

163071 0.796

163081 0.553

163087 0.515

163115 0.5

163131 0.408

163154 0.275

163175 0.07

163223 0.433

163227 0.219

163227 0.35

163255 0.502

163259 0

163351 0.215

163479 0.077

163589 0.13

163590 0.195

163702 0.262

163720 0.441

163742 0.423

163747 0.198

163782 0.164

163786 0.084

163859 0.219

163882 0.197

163933 0.019

164022 0.086

164045 0.274

164091 0.099

164127 0.303

164284 0.191

164312 0.236

164380 0.227

164395 0.066

164592 0.215

164633 0

164656 0.089

164668 0.457

164781 0.061

164832 0.129

165055 0.129

165057 0.256

165100 0.421

165186 0.174

165215 0.095

165324 0.087

165530 0.347

165545 0.092

165721 0.104

165829 0.187

165918 0.25

166012 0.124

166336 0.025

166348 0.025

166378 0.094

166379 0.189

166614 0.018

166647 0.033

166785 0.105

166793 0.165

166863 0.013

166929 0.047

166979 0.202

167127 0.231

167153 0.042

167227 0.041

167359 0.606

167410 0.028

167465 0.075

167555 0.081

167681 0.143

167691 0.149

167826 0.008

167838 0.181

168002 0.309

168090 0.32

168391 0.226

168400 0.655

168417 0.475

168474 0.052

168507 0.282

168667 0.042

168850 0.051

168975 0.311

169026 0.12

169044 0.109

169166 0.16

169200 0.036

169270 0.551

169522 0.103

169611 0.083

169675 0.591

169714 0.642

169792 0.079

169841 0.126

169966 0.138

170062 0.615

170082 0.172

170261 0.144

170302 0.018

170392 0.056

170463 0.068

170482 0.393

170506 0.043

170685 0.03

170690 0.098

170691 0.054

170692 0.065

170712 0.354

170825 0.057

170850 0.018

170954 0.136

171017 0.023

171019 0.066

171023 0.157

171024 0.078

171389 0.193

171425 0.08

171558 0.367

171568 0.009

171586 0.036

192111 0.014

192134 0.171

192666 0.153

192669 0.006

192670 0.006

192683 0.006

195814 0.264

195828 0.053

195977 0.445

196051 0.006

196294 0.032

196374 0.224

196383 0.127

196385 0.033

196385 0.04

196385 0.12

196403 0.084

196441 0.045

196446 0.173

196463 0.112

196477 0.239

196483 0.184

196500 0.033

196513 0.734

196515 0.017

196527 0.055

196528 0.056

196549 0.191

196740 0.068

196792 0.452

196883 0.043

196951 0.255

196996 0.142

197021 0.099

197131 0.042

197257 0.097

197258 0.091

197259 0.274

197320 0.393

197322 0.097

197335 0.241

197350 0.205

197358 0.141

197370 0.051

197407 0.09

198437 0.25

199221 0.138

199223 0.145

199675 0.42

199692 0.352

199699 0.395

199720 0.171

199731 0.024

199786 0.239

199857 0.149

199870 0.011

199920 0.286

199964 0.387

199974 0.431

200010 0.078

200014 0.669

200081 0.048

200095 0.38

200132 0.143

200150 0.039

200159 0.191

200162 0.163

200186 0.039

200205 0.25

200350 0.246

200373 0.201

200383 0.042

200407 0.092

200424 0.053

200504 0.198

200523 0.2

200539 0.098

200576 0.035

200634 0.09

200728 0.106

200734 0.032

200810 0.194

200844 0.157

200879 0.126

200916 0.021

200931 0.099

200933 0.007

200942 0.013

200958 0.622

201134 0.357

201158 0.056

201163 0.04

201164 0.095

201175 0.159

201176 0.068

201181 0.08

201191 0.03

201243 0.251

201254 0.119

201255 0.098

201266 0.052

201283 0.185

201292 0.261

201294 0.074

201299 0.18

201305 0.128

201456 0.323

201516 0.461

201562 0.032

201595 0.013

201625 0.063

201626 0.064

201627 0.031

201633 0.307

201725 0.09

201780 0.058

201798 0.086

201799 0.247

201895 0.068

201931 0.123

201965 0.052

201973 0.158

202018 0.029

202051 0.073

202151 0.177

202243 0.167

202309 0.311

202333 0.206

202915 0.095

203054 0.121

203068 0

203102 0.348

203107 0.03

203111 0.34

203190 0.019

203197 0.074

203228 0.01

203259 0.006

203260 0.195

203328 0.21

203427 0.072

203429 0.197

203430 0.262

203447 0.196

203522 0.016

203859 0.118

204219 0.129

204474 0.171

204801 0.749

204851 0.013

204962 0.196

205147 0.249

205327 0.125

205428 0.027

205564 0.097

205860 0.249

206338 0.28

206358 0.081

206938 0.061

219285 0.172

219333 0.008

219402 0.239

219409 0.009

219445 0.353

219527 0.037

219539 0.008

219541 0.014

219578 0.216

219595 0.071

219621 0.166

219623 0.192

219654 0

219670 0.069

219681 0.1

219699 0.041

219736 0.292

219738 0.034

219749 0.133

219770 0.368

219771 0.013

219790 0.165

219793 0.215

219844 0.105

219855 0.081

219899 0.013

219902 0.127

219927 0.096

219931 0.156

219938 0.172

219972 0.156

219988 0.02

219990 0.332

220001 0.117

220002 0.106

220004 0.176

220032 0.331

220042 0.259

220047 0.237

220064 0.139

220074 0.083

220081 0.666

220082 0.134

220107 0.104

220108 0.425

220115 0.199

220134 0.159

220136 0.199

220164 0.003

220213 0.106

220296 0.028

220323 0.073

220382 0.125

220388 0.141

220416 0.432

220441 0.013

220686 0.042

220766 0.041

220929 0.244

220963 0.068

220965 0.091

220972 0.038

220988 0

220992 0.391

221002 0.034

221035 0.04

221044 0.093

221078 0.114

221079 0.019

221091 0.235

221092 0.079

221120 0.078

221143 0.08

221150 0.248

221154 0.084

221184 0.033

221188 0.213

221191 0.292

221223 0.25

221264 0.189

221301 0.167

221303 0.212

221336 0.134

221391 0.022

221393 0.222

221395 0.182

221400 0.255

221409 0.136

221421 0.076

221443 0.019

221458 0.055

221468 0.293

221472 0.112

221477 0.107

221481 0.094

221496 0.082

221527 0.014

221545 0.122

221656 0.075

221662 0.075

221687 0.008

221692 0.021

221710 0.328

221711 0.383

221756 0.658

221785 0.133

221806 0.101

221806 0.251

221833 0.011

221895 0.004

221908 0.16

221935 0.052

221937 0.118

221960 0.025

222068 0.032

222166 0.311

222171 0.196

222183 0.859

222194 0.096

222223 0.03

222229 0.136

222234 0.234

222235 0.206

222236 0.065

222256 0.16

222389 0.115

222484 0.059

222487 0.3

222521 0.107

222545 0.119

222546 0.058

222553 0.019

222584 0.095

222643 0.096

222658 0.049

222659 0.286

222662 0.012

222663 0.024

222698 0.195

222699 0.149

222826 0.244

222865 0.187

222950 0.035

222967 0.177

223075 0.295

223082 0.064

223117 0.04

225689 0.19

245806 0.069

245932 0.228

245935 0.345

245972 0.04

245973 0.047

246126 0.503

246175 0.012

246176 0.264

246184 0.076

246213 0.037

246269 0.068

246329 0.032

246330 0.016

246778 0.145

252839 0.013

252969 0.162

252983 0.133

252995 0.011

253012 0.088

253017 0.099

253143 0.158

253152 0.06

253190 0.222

253430 0.121

253461 0.107

253558 0.061

253559 0.007

253635 0.059

253714 0.142

253724 0.395

253725 0.156

253738 0.003

253769 0.255

253782 0.022

253827 0.043

253832 0.046

253842 0.473

253943 0.007

253959 0.026

253980 0.033

253982 0.024

254013 0.135

254042 0.046

254048 0.037

254050 0.223

254065 0.035

254102 0.899

254122 0.048

254158 0.292

254170 0.033

254187 0.3

254225 0.128

254228 0.092

254240 0.185

254251 0.069

254268 0.388

254394 0.045

254427 0.204

254428 0.015

254439 0.343

254528 0.099

254531 0.036

254571 0.043

254773 0.147

254778 0.108

254827 0.137

254887 0.058

254956 0.133

255022 0.045

255027 0.233

255057 0.075

255061 0.326

255104 0.112

255119 0.092

255180 0.349

255189 0.172

255220 0.297

255231 0.088

255239 0.129

255326 0.044

255349 0.309

255394 0.121

255403 0.386

255426 0.077

255488 0.07

255631 0.265

255738 0.077

255743 0.065

255758 0.061

255762 0.202

255798 0.151

255812 0.082

255877 0.051

255926 0.687

255928 0.052

255967 0.013

256006 0.279

256051 0.671

256076 0.166

256112 0.353

256158 0.094

256158 0.128

256227 0.103

256281 0.106

256306 0.042

256309 0.204

256329 0.352

256355 0.131

256356 0.132

256364 0.033

256380 0.116

256394 0.158

256435 0.086

256471 0.113

256472 0.018

256536 0.142

256586 0.079

256643 0.227

256646 0.221

256691 0.073

256714 0.134

256764 0.181

256815 0.335

256933 0.26

256949 0.145

256957 0.241

256979 0.1

256987 0.113

257019 0.051

257044 0.304

257062 0.389

257101 0.329

257144 0.291

257160 0.014

257169 0.466

257194 0.025

257202 0.134

257218 0.081

257313 0.358

257364 0.018

257397 0.024

257407 0.257

257415 0.064

257629 0.123

258010 0.108

259173 0.115

259217 0.024

259230 0.01

259232 0.012

259236 0.036

259266 0.194

260293 0.181

260429 0.116

260436 0.432

261726 0.019

261729 0.025

261734 0.137

266629 0.018

266675 0.112

266722 0.016

266727 0.052

266971 0.064

280655 0.3

282616 0.273

282617 0.263

282618 0.357

282679 0.11

282966 0.16

282969 0.128

282974 0.048

282996 0.114

283008 0.329

283071 0.105

283078 0.056

283120 0.338

283129 0.319

283149 0.029

283150 0.191

283152 0.195

283176 0.24

283197 0.795

283208 0.055

283209 0.033

283212 0.031

283229 0.198

283232 0.269

283234 2.095

283237 0.023

283238 0.335

283248 0.025

283254 0.051

283294 0.219

283297 0.07

283298 0.088

283310 0.099

283316 0.592

283337 0.024

283349 0.031

283358 0.469

283373 0.005

283375 0.101

283377 0.412

283383 0.143

283385 0.103

283417 0.108

283431 0.102

283455 0.021

283459 0.152

283461 0.373

283463 1.118

283464 0.075

283471 0.263

283489 0.141

283514 0.02

283537 0.151

283576 0.065

283578 0.077

283600 0.077

283629 0.082

283635 0.086

283638 0.116

283651 0.153

283652 0.086

283659 0.049

283677 0.197

283726 0.092

283742 0.059

283755 0.054

283777 0.976

283807 0.142

283820 0.042

283847 0.178

283848 0.219

283849 0.139

283869 0.223

283870 0.229

283897 0.184

283933 0.649

283951 0.245

283970 0.052

283987 0.027

283989 0.127

283999 0.172

284013 0.219

284021 0.41

284040 0.41

284047 0.308

284058 0.041

284067 0.08

284076 0.248

284086 0.034

284098 0.144

284099 0.23

284106 0.037

284110 0.063

284111 0.17

284116 0.274

284119 0.104

284129 0.115

284131 0.138

284158 0.134

284161 0.05

284176 0.1

284207 0.143

284208 0.079

284217 0.151

284221 0.041

284222 0.077

284252 0

284266 0.116

284297 0.13

284307 0.543

284325 0.141

284339 0.02

284345 0.046

284345 0.172

284346 0.108

284348 0.181

284349 0.382

284358 0.151

284361 0.267

284369 0.604

284370 0.389

284371 0.363

284382 0.16

284391 0.341

284418 0.314

284422 0.413

284427 0.081

284439 0.054

284443 0.35

284451 0.109

284459 0.498

284467 0.125

284485 0.083

284486 0.124

284521 0.097

284525 0.189

284541 0.167

284542 0.454

284546 0.315

284613 0.049

284654 0.071

284656 0.039

284695 0.052

284697 0.214

284716 0.049

284723 0.068

284729 0.113

284747 0.198

284904 0.1

284944 0.096

284948 0.229

284992 0.153

284996 0.117

285016 0.165

285025 0.232

285051 0.191

285074 0.014

285116 0.05

285126 0.213

285148 0.104

285172 0.024

285175 0.04

285180 0.335

285195 0.044

285203 0.763

285216 0.13

285231 0.547

285237 0.093

285282 0.045

285313 0.227

285315 0.149

285331 0.219

285335 0.198

285343 0.107

285346 0.249

285359 0.405

285367 0.171

285368 0.169

285381 0.017

285386 0.102

285429 0.188

285489 0.117

285513 0.288

285521 0.14

285525 0.16

285555 0.25

285590 0.021

285598 0.047

285601 0.16

285605 0.078

285613 0.09

285636 0.244

285641 0.146

285671 0.103

285672 0.052

285676 0.12

285704 0.062

285753 0.201

285755 0.18

285761 0.121

285782 0.295

285848 0.359

285855 0.093

285877 0.494

285888 0.198

285927 0.145

285955 0.28

285966 0.162

285971 0.123

285973 0.074

285989 0.696

286046 0.028

286053 0.087

286075 0.22

286077 0.837

286097 0.019

286128 0.783

286133 0.073

286151 0.222

286187 0.06

286204 0.13

286205 0.016

286207 0.31

286234 0.659

286256 0.394

286262 0.176

286319 0.161

286336 0.003

286343 0.048

286380 0.256

286410 0.027

286436 0.491

286451 0.11

286514 0.3

286554 0.263

286826 0.091

287015 0.074

317649 0.012

317662 0.106

317671 0.071

317702 0.525

317719 0.005

317754 0.457

317761 0.168

317781 0.143

319089 0.484

319101 0.094

326332 0.23

326340 0.253

326342 0.214

326624 0.14

326625 0.069

327657 0.196

333926 0.026

333929 0.094

337876 0.052

337880 0.101

337882 0.253

337970 0.136

338321 0.522

338323 0.328

338328 0.302

338339 0.255

338376 0.326

338440 0.134

338557 0.08

338567 0.193

338596 0.103

338599 0.118

338645 0.095

338657 0.086

338661 0.416

338692 0.064

338707 0.089

338761 0.017

338773 0.21

338785 0.1

338811 0.014

338879 0.192

338917 0.016

338949 0.41

339005 0.269

339010 0.449

339047 0.129

339123 0.098

339175 0.129

339210 0.096

339221 0.112

339229 0.222

339231 0.044

339324 0.078

339327 0.504

339366 0.122

339390 0.255

339398 0.035

339403 0.14

339416 0.156

339448 0.225

339451 0.537

339457 0.142

339479 0.011

339487 0.037

339488 0.035

339512 0.315

339541 0.114

339559 0.156

339669 0.221

339674 0.821

339745 0.018

339766 0.174

339778 0.231

339779 0.291

339809 0.269

339829 0.138

339834 0.286

339906 0.338

339976 0.113

339983 0.011

340024 0.08

340061 0.213

340069 0.201

340075 0.022

340205 0.194

340206 0.439

340252 0.426

340273 0.124

340277 0.065

340286 0.163

340348 0.018

340351 0.066

340371 0.03

340390 0.43

340419 0.018

340441 0.387

340481 0.014

340485 0.034

340526 0.197

340529 0.075

340533 0.075

340547 0.177

340554 0.052

340562 0.606

340595 0.255

340596 0.056

340602 0.932

340665 0.081

340706 0.116

340745 0.169

340811 0.35

340990 0.151

341019 0.459

341032 0.106

341116 0.337

341359 0.041

341405 0.155

341640 0.075

341676 0.18

341883 0.1

342035 0.07

342125 0.112

342132 0.392

342357 0.106

342372 0.288

342510 0.303

342527 0.09

342538 0.064

342574 0.062

342667 0.033

342892 0.537

342897 0.163

342898 0.159

342926 0.486

342945 0.158

343035 0.082

343070 0.537

343099 0.074

343263 0.094

343413 0.395

343450 0.051

343472 0.008

343637 0.089

343641 0.083

343702 0.032

343990 0.247

344018 0.226

344148 0.228

344387 0.075

344454 0.048

344658 0.253

344752 0.122

344758 0.146

344805 0.052

344807 0.496

344892 0.084

344901 0.138

344905 0.229

345193 0.237

345274 0.179

345275 0.106

345462 0.112

345557 0.01

345611 0.364

345651 0.017

345757 0.213

345778 0.065

346389 0.139

346653 0.212

346673 0.197

346689 0.273

347051 0.136

347088 0.284

347148 0.245

347240 0.256

347365 0.18

347404 0.027

347516 0.352

347734 0.049

347735 0.087

347853 0.103

347862 0.058

347902 0.069

348093 0.052

348094 0.089

348180 0.134

348235 0.113

348303 0.525

348327 0.436

348378 0.239

348487 0.118

348793 0.091

348807 0.245

348840 0.587

348926 0.173

348932 0.167

348980 0.025

348995 0.043

349035 0.24

349152 0.09

349565 0.1

349633 0.673

349667 0.026

350383 0.161

352909 0.143

352954 0.26

353088 0.375

353116 0.036

353132 0.129

353135 0.111

353149 0.101

353189 0.107

353238 0.228

353274 0.255

353299 0.266

353322 0.168

353376 0.163

353497 0.187

353500 0.075

353514 0.319

359710 0.134

359948 0.018

360023 0.033

360132 0.068

373156 0.177

373509 0.135

373856 0.184

373863 0.075

374286 0.251

374291 0.076

374308 0.236

374354 0.102

374355 0.171

374378 0.01

374403 0.07

374407 0.083

374443 0.342

374454 0.166

374462 0.032

374467 0.162

374569 0.112

374618 0.141

374650 0.883

374654 0.092

374655 0.029

374666 0.105

374739 0.133

374786 0.264

374860 0.465

374864 0.384

374868 0.04

374879 0.433

374897 0.381

374899 0.188

374907 0.149

374946 0.14

374955 0.507

374973 0.204

374986 0.094

374992 0.141

375033 0.09

375057 0.012

375189 0.09

375190 0.143

375248 0.629

375251 0.585

375287 0.291

375307 0.182

375316 0.321

375318 0.167

375323 0.01

375337 0.111

375347 0.138

375484 0.118

375519 0.324

375567 0.046

375593 0.118

375611 0.021

375612 0.005

375616 0.2

375686 0.175

375690 0.114

375743 0.058

375748 0.039

375757 0.304

375790 0.116

375940 0.149

376132 0.062

376267 0.041

376412 0.264

376497 0.059

376693 0.149

376844 0.203

376940 0.122

377007 0.086

377677 0.05

377711 0.132

378464 0.037

378708 0.127

378884 0.099

386617 0.027

387032 0.185

387103 0.17

387104 0.016

387129 0.06

387254 0.156

387263 0.152

387332 0.194

387357 0.111

387496 0.061

387509 0.068

387521 0.042

387597 0.046

387601 0.358

387628 0.029

387640 0.035

387646 0.209

387680 0.154

387693 0.232

387694 0.054

387712 0.175

387748 0.089

387750 0.218

387758 0.031

387856 0.046

387893 0.06

387914 0.042

387921 0.096

387990 0.264

388021 0.042

388112 0.266

388115 0.21

388121 0.099

388228 0.45

388272 0.006

388323 0.264

388324 0.233

388335 0.123

388336 0.016

388364 0.188

388403 0

388468 0.451

388507 0.441

388531 0.092

388536 0.499

388561 0.443

388564 0.193

388566 0.534

388567 0.494

388581 0.176

388585 0.051

388591 0.615

388610 0.068

388611 0.111

388650 0.008

388714 0.085

388722 0.204

388730 0.19

388762 0.33

388818 0.293

388960 0.44

388965 0.115

388969 0.173

389058 0.84

389064 0.002

389072 0.06

389073 0.199

389075 0.287

389084 0.131

389170 0.164

389177 0.121

389197 0.3

389203 0.184

389206 0.032

389208 0.072

389320 0.203

389336 0.203

389383 0.478

389384 0.385

389400 0.183

389421 0.081

389429 0.295

389434 0.092

389524 0.118

389549 0.029

389558 0.138

389634 0.527

389643 0.134

389668 0.152

389677 0.15

389690 0.207

389722 0.168

389756 0.417

389760 0.171

389763 0.647

389799 0.04

389835 0.087

389840 0.084

389852 0.179

389874 0.243

389932 0.341

390054 0.105

390212 0.15

390275 0.085

390443 0.667

390616 0.095

390637 0.147

390664 0.379

390792 0.159

390927 0.437

390928 0.085

391059 0.161

391253 0.306

391356 0.042

391622 0.368

392255 0.066

392307 0.586

392309 0.098

392490 0.104

392636 0.097

392780 0.053

399512 0.047

399664 0.089

399671 0.226

399694 0.121

399726 1.135

399815 0.247

399909 0.037

399949 0.212

399967 0.227

399968 0.558

400110 0.476

400120 0.023

400258 0.328

400451 0.098

400464 0.476

400508 0.405

400629 0.273

400668 0.16

400673 0.353

400720 0.452

400745 0.064

400831 0.21

400891 0.265

400916 0.067

400961 0.061

400986 1.029

401013 0.118

401024 0.273

401036 0.428

401081 0.197

401115 0.069

401124 0.131

401127 0.051

401136 0.211

401137 0.566

401138 0.234

401147 0.082

401190 0.033

401236 0.213

401258 0.27

401262 0.077

401263 0.023

401303 0.574

401331 0.083

401393 0.297

401428 0.179

401428 0.434

401474 0.035

401494 0.067

401498 0.044

401541 0.202

401546 0.231

401548 0.027

401612 0.061

401898 0.463

401944 0.229

401983 0.421

402055 0.209

402176 0.142

402415 0.047

402573 0.529

403113 0.498

403313 0.067

403314 0.26

403315 0.227

403341 0.036

404093 0.07

404203 0.128

404217 0.018

404220 0.197

404636 0.05

404734 0.023

404785 0.418

405752 0.023

405753 0.115

407738 0.004

407835 0.067

407977 0.116

408050 0.036

414149 0.094

414152 0.244

414328 0.18

414927 0.247

431705 0.207

431707 0.023

439921 0.216

439996 0.574

440026 0.02

440044 0.128

440093 0.025

440097 0.134

440145 0.043

440163 0.174

440184 0.131

440193 0.049

440193 0.119

440248 0.088

440275 0.055

440278 0.21

440279 0.028

440356 0.356

440434 0.038

440435 0.176

440574 0.055

440590 0.147

440695 0.235

440730 0.103

440738 0.406

440792 0.099

440822 0.418

440888 0.124

440993 0.404

441024 0.044

441140 0.333

441150 0.335

441168 0.22

441193 0.239

441194 0.042

441220 0.037

441234 0.483

441250 2.264

441253 0.18

441476 0.318

441601 0.326

442245 0.237

442247 0.618

442582 0.301

445372 0.253

445571 0.095

445582 0.431

445815 0.495

448831 0.624

449520 0.299

474343 0.28

492304 0.323

493829 0.036

493856 0.021

493860 0.292

493911 0.109

494127 0.113

494141 0.15

494188 0.058

497190 0.103

503497 0.268

541473 0.082

552889 0.045

553115 0.082

553158 0.14

554175 0.397

554208 0.579

554226 0.577

554234 0.075

554235 0.051

574029 0.371

595101 0.125

595135 0.185
