# Supplementary material for: microRNA evolution in a human transcription factor and microRNA regulatory network
Source: BMC Syst Biol. 2010 Jun 29;4:90. doi: 10.1186/1752-0509-4-90 (PMC2914650; doi:10.1186/1752-0509-4-90)
Supplement: Additional file 4 — Evolutionary rates of miRNA genes. This file contains the evolutionary rates of miRNA genes. [file 1752-0509-4-90-S4.DOC]

**Additional file 4.** This file contains the evolutionary rates of human microRNA genes.

microRNA Evolutionary rate

let-7a .062780269058296

let-7b .024390243902439

let-7c 0

let-7d 1.16279069767442E-02

let-7e 1.28205128205128E-02

let-7f 1.78571428571429E-02

let-7g 0

let-7i 2.40963855421687E-02

mir-1 .051948051948052

mir-100 .113924050632911

mir-101 7.89473684210526E-02

mir-103 3.24675324675325E-02

mir-105 .0375

mir-106a .0125

mir-106b .037037037037037

mir-107 .075

mir-10a 9.17431192660551E-02

mir-10b .036697247706422

mir-1178 1.11111111111111E-02

mir-1179 .255555555555556

mir-1180 0

mir-1181 .0625

mir-1182 4.16666666666667E-02

mir-1183 .545454545454545

mir-1184 1.02040816326531E-02

mir-1185 7.05882352941176E-02

mir-1197 4.59770114942529E-02

mir-1200 .106666666666667

mir-1201 0

mir-1202 7.31707317073171E-02

mir-1203 2.38095238095238E-02

mir-1204 3.03030303030303E-02

mir-1205 1.61290322580645E-02

mir-1206 5.17241379310345E-02

mir-1207 6.97674418604651E-02

mir-1208 0

mir-122 3.57142857142857E-02

mir-1224 7.14285714285714E-02

mir-1225 0

mir-1226 0

mir-1227 .655172413793103

mir-1228 5.55555555555556E-02

mir-1229 .573529411764706

mir-1231 0

mir-1233 .123456790123457

mir-1234 .313253012048193

mir-1236 5.94059405940594E-02

mir-1237 5.94059405940594E-02

mir-1238 .109756097560976

mir-124 .015625

mir-1243 2.17391304347826E-02

mir-1244 1.48809523809524E-02

mir-1245 .072463768115942

mir-1246 6.94444444444444E-02

mir-1247 7.40740740740741E-03

mir-1248 4.76190476190476E-02

mir-1249 6.15384615384615E-02

mir-1250 9.82142857142857E-02

mir-1251 0

mir-1252 .78125

mir-1254 8.33333333333333E-02

mir-1255b .0546875

mir-1256 4.23728813559322E-02

mir-1257 .301724137931034

mir-1258 5.55555555555556E-02

mir-1259 9.09090909090909E-03

mir-125a 7.05882352941176E-02

mir-125b 2.28571428571429E-02

mir-126 .25

mir-1260 2.77777777777778E-02

mir-1261 1.23456790123457E-02

mir-1262 7.60869565217391E-02

mir-1263 .4

mir-1264 5.88235294117647E-02

mir-1265 7.05882352941176E-02

mir-1266 .144578313253012

mir-1267 6.49350649350649E-02

mir-1269 .105769230769231

mir-127 2.08333333333333E-02

mir-1270 .182926829268293

mir-1271 4.70588235294118E-02

mir-1272 .0625

mir-1273 3.92156862745098E-02

mir-1274a .171428571428571

mir-1275 6.32911392405063E-02

mir-1276 4.87804878048781E-02

mir-1277 .220779220779221

mir-1278 .0375

mir-1279 3.27868852459016E-02

mir-128 1.23456790123457E-02

mir-1280 5.37634408602151E-02

mir-1282 0

mir-128-2 8.43373493975904E-02

mir-1283 .13953488372093

mir-1284 8.40336134453782E-02

mir-1285 .241379310344828

mir-1286 6.49350649350649E-02

mir-1287 4.49438202247191E-02

mir-1288 0

mir-1289 5.13833992094862E-02

mir-129 .01875

mir-1290 .272727272727273

mir-1291 2.32558139534884E-02

mir-1295 3.84615384615385E-02

mir-1296 .010989010989011

mir-1297 1.31578947368421E-02

mir-1298 .045045045045045

mir-1300 4.59770114942529E-02

mir-1302 6.86886708296164E-02

mir-1303 .105882352941176

mir-1304 2.22222222222222E-02

mir-1305 7.05882352941176E-02

mir-1306 0

mir-1307 0

mir-1308 .132075471698113

mir-130a .170454545454545

mir-130b .518518518518518

mir-132 .03

mir-1322 8.57142857142857E-02

mir-1323 2.77777777777778E-02

mir-1324 .136842105263158

mir-133a 9.57446808510638E-02

mir-133b 8.47457627118644E-02

mir-134 1.38888888888889E-02

mir-135a 2.65957446808511E-02

mir-135b 1.04166666666667E-02

mir-136 1

mir-137 0

mir-138 9.63855421686747E-02

mir-139 0

mir-140 2.02020202020202E-02

mir-141 4.25531914893617E-02

mir-142 5.81395348837209E-02

mir-143 .104761904761905

mir-144 1.17647058823529E-02

mir-145 2.29885057471264E-02

mir-1468 7.05882352941176E-02

mir-1469 2.17391304347826E-02

mir-146a 6.12244897959184E-02

mir-147 .366197183098592

mir-1471 3.57142857142857E-02

mir-147b 6.32911392405063E-02

mir-148a 2.98507462686567E-02

mir-148b 0

mir-149 .238636363636364

mir-150 .216867469879518

mir-151 .123595505617978

mir-152 0

mir-153-1 0

mir-153-2 0

mir-1537 6.66666666666667E-02

mir-1538 .133333333333333

mir-1539 .183673469387755

mir-154 8.43373493975904E-02

mir-155 .015625

mir-15a 1.21951219512195E-02

mir-15b 0

mir-16 5.95238095238095E-02

mir-17 .349397590361446

mir-181a 4.58715596330275E-02

mir-181b 2.53807106598985E-02

mir-181c 2.75229357798165E-02

mir-181d 2.20588235294118E-02

mir-182 8.25688073394495E-02

mir-1825 7.69230769230769E-02

mir-1826 .261904761904762

mir-1827 3.07692307692308E-02

mir-183 .018348623853211

mir-184 4.81927710843374E-02

mir-185 2.46913580246914E-02

mir-186 1.17647058823529E-02

mir-187 1.85185185185185E-02

mir-188 7.05882352941176E-02

mir-18a .428571428571429

mir-18b 0

mir-190 4.76190476190476E-02

mir-1909 7.59493670886076E-02

mir-190b 1.28205128205128E-02

mir-191 .021978021978022

mir-1910 .139240506329114

mir-1911 1.26582278481013E-02

mir-1912 3.79746835443038E-02

mir-1913 .126582278481013

mir-1914 7.59493670886076E-02

mir-1915 2.53164556962025E-02

mir-192 0

mir-193a 6.89655172413793E-02

mir-193b .024390243902439

mir-194 7.14285714285714E-02

mir-194-2 3.57142857142857E-02

mir-195 2.32558139534884E-02

mir-196a 3.37078651685393E-02

mir-197 0

mir-1972 3.94736842105263E-02

mir-1973 .302325581395349

mir-1974 .144927536231884

mir-1975 4.05405405405405E-02

mir-1976 .156862745098039

mir-1977 .243589743589744

mir-1979 .573333333333333

mir-198 8.19672131147541E-02

mir-199a 1.11731843575419E-02

mir-19b .116022099447514

mir-200a 1.12359550561798E-02

mir-200c 0

mir-203 .055045871559633

mir-204 0

mir-205 8.25688073394495E-02

mir-2052 0

mir-2053 8.88888888888889E-02

mir-2054 0

mir-206 8.23529411764706E-02

mir-208a 7.14285714285714E-02

mir-208b 0

mir-20a .242857142857143

mir-20b 1.47058823529412E-02

mir-21 0

mir-210 9.17431192660551E-03

mir-211 2.75229357798165E-02

mir-2113 0

mir-212 .055045871559633

mir-214 2.75229357798165E-02

mir-215 6.42201834862385E-02

mir-216a .036697247706422

mir-216b 1.23456790123457E-02

mir-217 .055045871559633

mir-218 4.58715596330275E-03

mir-219 4.39024390243902E-02

mir-220a .128440366972477

mir-220b 5.81395348837209E-02

mir-220c 9.75609756097561E-02

mir-221 8.25688073394495E-02

mir-222 .394495412844037

mir-223 4.58715596330275E-02

mir-224 .0375

mir-23a 1.38888888888889E-02

mir-23b 5.20833333333333E-02

mir-24 .079136690647482

mir-25 6.02409638554217E-02

mir-26a 6.28930817610063E-03

mir-26b .697368421052632

mir-27a .012987012987013

mir-27b 2.08333333333333E-02

mir-28 .105882352941176

mir-296 7.59493670886076E-02

mir-297 7.69230769230769E-02

mir-298 .218390804597701

mir-299 .032258064516129

mir-29a 0

mir-29b .03125

mir-29c 6.89655172413793E-02

mir-300 .109756097560976

mir-301a 0

mir-301b .012987012987013

mir-302a 4.41176470588235E-02

mir-302b .125

mir-302c 7.46268656716418E-02

mir-302d 4.47761194029851E-02

mir-302e 1.40845070422535E-02

mir-302f .04

mir-30a 1.42857142857143E-02

mir-30b 6.89655172413793E-02

mir-30c 1.25786163522013E-02

mir-30d 0

mir-30e .021978021978022

mir-31 0

mir-32 0

mir-320a 8.64197530864197E-02

mir-320b .111627906976744

mir-320c 8.08823529411765E-02

mir-320d 4.25531914893617E-02

mir-323 4.70588235294118E-02

mir-324 .024390243902439

mir-325 .154639175257732

mir-329 6.17283950617284E-02

mir-330 5.37634408602151E-02

mir-331 .032258064516129

mir-335 8.60215053763441E-02

mir-337 0

mir-338 .287878787878788

mir-339 9.67741935483871E-02

mir-33b 1.05263157894737E-02

mir-340 3.19148936170213E-02

mir-342 .102040816326531

mir-345 6.18556701030928E-02

mir-346 1.06382978723404E-02

mir-34a .036697247706422

mir-34b .156626506024096

mir-34c .118421052631579

mir-361 7.04225352112676E-02

mir-362 .015625

mir-363 1.35135135135135E-02

mir-365 6.12244897959184E-02

mir-367 4.47761194029851E-02

mir-369 1.44927536231884E-02

mir-370 .121621621621622

mir-371 4.54545454545455E-02

mir-372 6.06060606060606E-02

mir-373 .102941176470588

mir-374a .140845070422535

mir-374b 1.40845070422535E-02

mir-375 .285714285714286

mir-376a 0

mir-376b 0

mir-376c 0

mir-377 1.47058823529412E-02

mir-378 .123076923076923

mir-379 6.06060606060606E-02

mir-380 .1

mir-381 1.35135135135135E-02

mir-382 0

mir-383 1.38888888888889E-02

mir-384 3.44827586206897E-02

mir-409 0

mir-410 2.53164556962025E-02

mir-411 .2

mir-412 1.11111111111111E-02

mir-421 0

mir-422a 5.61797752808989E-02

mir-423 0

mir-424 4.12371134020619E-02

mir-425 1.16279069767442E-02

mir-429 4.87804878048781E-02

mir-431 7.07964601769911E-02

mir-432 6.45161290322581E-02

mir-433 4.34782608695652E-02

mir-448 5.45454545454545E-02

mir-449a 2.22222222222222E-02

mir-449b 1.04166666666667E-02

mir-450a .111111111111111

mir-450b .025974025974026

mir-451 .028169014084507

mir-452 0

mir-453 1.26582278481013E-02

mir-454 2.63157894736842E-02

mir-455 0

mir-484 0

mir-485 0

mir-486 2.98507462686567E-02

mir-487a 6.32911392405063E-02

mir-487b 0

mir-488 1.21951219512195E-02

mir-489 .481927710843373

mir-490 0

mir-491 0

mir-492 9.56521739130435E-02

mir-493 1.13636363636364E-02

mir-494 .0125

mir-495 .679012345679012

mir-496 8.91089108910891E-02

mir-497 0

mir-498 .040650406504065

mir-499 4.95867768595041E-02

mir-500 2.40963855421687E-02

mir-501 2.40963855421687E-02

mir-502 7.05882352941176E-02

mir-503 2.85714285714286E-02

mir-504 1.21951219512195E-02

mir-505 .072289156626506

mir-506 5.69105691056911E-02

mir-507 .559139784946237

mir-508 5.26315789473684E-02

mir-509 1

mir-510 2.73972602739726E-02

mir-511 .063953488372093

mir-512 7.22222222222222E-02

mir-513a .405511811023622

mir-513b 6.02409638554217E-02

mir-513c 9.63855421686747E-02

mir-514 2.29885057471264E-02

mir-515 .146341463414634

mir-516a .123595505617978

mir-516b .179190751445087

mir-517a 8.13953488372093E-02

mir-517b .136363636363636

mir-517c .276595744680851

mir-518a .1

mir-518b 7.31707317073171E-02

mir-518c .05

mir-518d .13953488372093

mir-518e .114942528735632

mir-518f 8.13953488372093E-02

mir-519a .141176470588235

mir-519b .225

mir-519c 5.81395348837209E-02

mir-519d 5.74712643678161E-02

mir-519e .180722891566265

mir-520a 3.57142857142857E-02

mir-520b .133333333333333

mir-520c .186046511627907

mir-520d 8.13953488372093E-02

mir-520e .151162790697674

mir-520f .255813953488372

mir-520g 8.98876404494382E-02

mir-521 .203488372093023

mir-522 8.13953488372093E-02

mir-523 5.81395348837209E-02

mir-524 5.81395348837209E-02

mir-525 .119047619047619

mir-526a .189189189189189

mir-526b .109756097560976

mir-527 .130952380952381

mir-532 4.44444444444444E-02

mir-539 .116883116883117

mir-541 2.40963855421687E-02

mir-542 .03125

mir-543 .012987012987013

mir-544 5.55555555555556E-02

mir-545 .238095238095238

mir-548a .0625

mir-548b .09375

mir-548c 5.20833333333333E-02

mir-548d 4.16666666666667E-02

mir-548f .438172043010753

mir-548g .227272727272727

mir-548h .210365853658537

mir-548i .973684210526316

mir-548j .243243243243243

mir-548k .426086956521739

mir-548l 9.41176470588235E-02

mir-548n .135135135135135

mir-548o .221238938053097

mir-548p .132530120481928

mir-549 .136842105263158

mir-550 3.64583333333333E-02

mir-551a 2.10526315789474E-02

mir-551b 3.15789473684211E-02

mir-552 .105263157894737

mir-553 5.97014925373134E-02

mir-554 8.42105263157895E-02

mir-555 6.31578947368421E-02

mir-556 6.38297872340425E-02

mir-557 6.18556701030928E-02

mir-558 .032258064516129

mir-561 .1875

mir-562 6.38297872340425E-02

mir-563 3.84615384615385E-02

mir-564 .473118279569893

mir-566 8.60215053763441E-02

mir-567 6.18556701030928E-02

mir-568 6.38297872340425E-02

mir-569 2.10526315789474E-02

mir-571 .136842105263158

mir-572 6.38297872340425E-02

mir-573 7.14285714285714E-02

mir-575 .150537634408602

mir-576 2.06185567010309E-02

mir-577 6.31578947368421E-02

mir-578 4.21052631578947E-02

mir-579 7.21649484536082E-02

mir-580 4.16666666666667E-02

mir-581 3.15789473684211E-02

mir-582 .257731958762887

mir-583 .27027027027027

mir-584 2.08333333333333E-02

mir-585 8.60215053763441E-02

mir-586 .145833333333333

mir-587 .726315789473684

mir-589 4.08163265306122E-02

mir-590 7.29166666666667E-02

mir-591 6.38297872340425E-02

mir-592 5.20833333333333E-02

mir-593 .101010101010101

mir-595 .105263157894737

mir-596 .210526315789474

mir-597 .0625

mir-599 3.19148936170213E-02

mir-600 .123711340206186

mir-601 7.69230769230769E-02

mir-602 .185567010309278

mir-603 8.33333333333333E-02

mir-604 .129032258064516

mir-606 .221052631578947

mir-609 .521276595744681

mir-611 3.03030303030303E-02

mir-612 .333333333333333

mir-613 .117021276595745

mir-614 .101123595505618

mir-615 2.10526315789474E-02

mir-616 7.29166666666667E-02

mir-617 .25

mir-618 .103092783505155

mir-619 .112244897959184

mir-620 .5

mir-621 8.42105263157895E-02

mir-622 .136842105263158

mir-623 5.15463917525773E-02

mir-624 4.16666666666667E-02

mir-625 8.33333333333333E-02

mir-626 .021505376344086

mir-628 1.06382978723404E-02

mir-630 .0625

mir-631 0

mir-632 5.37634408602151E-02

mir-633 5.15463917525773E-02

mir-635 .103092783505155

mir-636 4.08163265306122E-02

mir-637 9.18367346938776E-02

mir-638 3.03030303030303E-02

mir-639 .412371134020619

mir-640 2.10526315789474E-02

mir-642 8.33333333333333E-02

mir-643 8.33333333333333E-02

mir-644 .43010752688172

mir-645 .10752688172043

mir-647 3.15789473684211E-02

mir-648 .236559139784946

mir-649 .302083333333333

mir-650 4.21052631578947E-02

mir-651 4.16666666666667E-02

mir-652 4.12371134020619E-02

mir-653 3.15789473684211E-02

mir-654 0

mir-656 .025974025974026

mir-657 9.27835051546392E-02

mir-659 .822916666666667

mir-660 .322916666666667

mir-661 7.95454545454545E-02

mir-662 .074468085106383

mir-663 .108695652173913

mir-663b .149122807017544

mir-664 7.40740740740741E-02

mir-665 0

mir-668 1.53846153846154E-02

mir-671 2.56410256410256E-02

mir-675 2.77777777777778E-02

mir-708 0

mir-7-1 0

mir-7-2 .100917431192661

mir-7-3 2.75229357798165E-02

mir-744 1.03092783505155E-02

mir-758 3.44827586206897E-02

mir-760 2.53164556962025E-02

mir-765 8.84955752212389E-02

mir-766 4.54545454545455E-02

mir-767 6.48148148148148E-02

mir-770 4.12371134020619E-02

mir-802 .021505376344086

mir-873 0

mir-874 .025974025974026

mir-875 0

mir-876 0

mir-877 3.52941176470588E-02

mir-885 0

mir-886 .1

mir-887 5.12820512820513E-02

mir-888 6.57894736842105E-02

mir-889 0

mir-890 6.57894736842105E-02

mir-891a 6.41025641025641E-02

mir-891b .448717948717949

mir-892a 5.40540540540541E-02

mir-892b 1

mir-9-1 2.27272727272727E-02

mir-9-2 2.32558139534884E-02

mir-920 6.75675675675676E-02

mir-921 .290909090909091

mir-922 .075

mir-924 .153846153846154

mir-92a 0

mir-92b 1.05263157894737E-02

mir-93 6.32911392405063E-02

mir-9-3 6.74157303370787E-02

mir-933 2.63157894736842E-02

mir-934 6.09756097560976E-02

mir-935 .566666666666667

mir-936 0

mir-937 8.23529411764706E-02

mir-938 .621951219512195

mir-939 .037037037037037

mir-940 .010752688172043

mir-941 1

mir-942 5.88235294117647E-02

mir-943 .129032258064516

mir-944 1.14942528735632E-02

mir-95 0

mir-96 .012987012987013

mir-98 1.69491525423729E-02

mir-99a 0

mir-99b 1.44927536231884E-02
